# Extracellular matrix phenotyping by imaging mass cytometry defines distinct cellular matrix environments associated with allergic airway inflammation

**DOI:** 10.1038/s44320-026-00234-5

**Published:** 2026-07-20

**Authors:** James E Parkinson, Morgan Bryant, Mohamed Ghafoor, Rebecca J Dodd, Hannah E Tompkins, Martin Fergie, Matthew O Burgess, Magnus Rattray, Tara E Sutherland

**Affiliations:** 1 Lydia Becker Institute of Immunology and Inflammation, School of Biological Sciences, Faculty of Biology, Medicine and Health, University of Manchester, Manchester, M139PT UK; 2 Manchester Cell-Matrix Centre, School of Biological Sciences, Faculty of Biology, Medicine and Health, University of Manchester, Manchester, M139PT UK; 3 Institute of Medical Sciences, School of Medicine, Medical Sciences and Nutrition, University of Aberdeen, Aberdeen, AB252ZD UK; 4 Division of Informatics, Imaging & Data Sciences, Faculty of Biology Medicine and Health, University of Manchester, Manchester, M139PT UK

**Keywords:** Immunology

## Abstract

The extracellular matrix (ECM) forms the scaffold in which cells reside and interact. The composition of this scaffold guides the development of local immune responses and tissue function. With the advent of multiplexed spatial imaging methodologies, investigating the intricacies of cellular spatial organisation is more accessible than ever. However, the relationship between cellular organisation and ECM composition has been broadly overlooked. Using imaging mass cytometry, we investigated the association between cellular niches and their surrounding matrix environment during allergic airway inflammation in two commonly used in-bred mouse strains. We have integrated both intracellular and extracellular analysis by first classifying cells according to their canonical marker expression and then utilising a novel pipeline to independently characterise their ECM environment. Applying this methodology across three distinct lung tissue regions we reveal region-specific and spatially constrained allergic inflammatory responses. In our model of allergic airway inflammation, recruited neutrophils were dispersed within the alveolar parenchyma, alongside a loss of alveolar type-I cells and an expansion of alveolar type-II cells. This activated parenchyma was associated with increased proximity to hyaluronan and chondroitin sulphate. In contrast, infiltrating CD11b^+^ and MHCII^+^ cells accumulated in the adventitial cuff specifically in BALB/c mice. This region was around the airway-adjacent pulmonary artery and aligned with an expansion of the subepithelial airway region. This expanded subepithelial region was enriched for closely interacting stromal and CD11b^+^ immune cells which overlaid regions enriched for type-I and type-III collagen. The detailed map of cell and matrix interactions generated here provides a critical resource for understanding ECM contribution to specific lung niches during allergic disease. It reveals strain-dependent differences in inflammation and remodelling and generates novel hypotheses for targeting aspects of remodelling during allergic airway pathology.

## Synopsis

Spatial profiling has traditionally emphasised the cellular landscape within tissues. Here, a pipeline is presented to systematically map both the ECM and cellular environments across distinct lung regions, providing insight into how matrix-cell interactions shape lung health.

- The lung tissue architecture is characterised by distinct matrix environments which are altered during allergic airway pathology in mice.
- Immune cell accumulation around the adventitial cuff correlates with a shift from a collagen-rich to a hyaluronan- and laminin-rich matrix environment.
- Stromal cell populations reorganise around active remodelling regions, including the airway subepithelial space, during allergic pathology.
- Macrophages localise to ECM remodelling sites enriched in S100a4^+^ fibroblasts.

## Abstract (web summary)

Spatial profiling has traditionally emphasised the cellular landscape within tissues. Here, a pipeline is presented to systematically map both the ECM and cellular environments across distinct lung regions, providing insight into how matrix-cell interactions shape lung health.

## Graphical abstract

**Figure:**
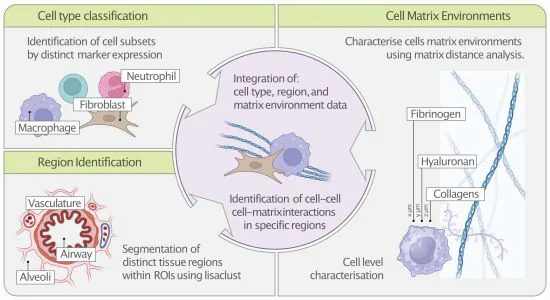

**Figure:**
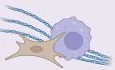

## Introduction

The extracellular matrix (ECM) is a highly heterogenous structure which comprises a vast array of molecules, including proteins, glycosaminoglycans, and proteoglycans (Naba et al, 2012). ECM molecules are organised into complex and diverse networks which provide distinct structural, mechanical and biological properties to an individual tissue (Lampi and Reinhart-King, 2018; Nakasaki et al, 2015; Zhuang et al, 2020; Hang et al, 2023; Guo et al, 2022). As such, the ECM is not only a framework within which cellular interactions occur (Valdoz et al, 2021), but is also an important transducer of cellular signals and responses (Hynes, 2009). Many of the immune system’s canonical signalling pathways are shaped by both direct and indirect interactions with the ECM (Sutherland et al, 2023). Therefore, it is vital to acknowledge the mutual role of the cellular ECM environment when studying a tissue’s response to challenges such as injury or disease.

Alterations in the composition and organisation of the ECM are present in almost all diseases, though to different extents (Tayebjee et al, 2005; Sun et al, 2021; Chuang et al, 2014; Derkacz et al, 2021; Gress et al, 1998; McQuitty et al, 2020; Imanaka-Yoshida et al, 2004). In respiratory diseases such as asthma, airway remodelling encapsulates a variety of processes including epithelial activation, smooth muscle hyperplasia, immune infiltration, and airway subepithelial ECM reorganisation (Burgess and Harmsen, 2022; Varricchi et al, 2024). Together, these features are a major driver of airway hyperresponsiveness (AHR) and therefore breathing difficulties in people with asthma (Pascual and Peters, 2005), yet the factors that contribute to airway remodelling are still poorly understood. Animal models of allergic airway inflammation and pathology can provide dynamic and mechanistic insight into the processes that drive airway remodelling. One such model, henceforth termed DRA, involves chronic biweekly exposure to three clinically relevant allergens: house dust mite, ragweed and Aspergillus (Goplen et al, 2009; Parkinson et al, 2025). DRA challenge induces the deposition of both type-I and III collagens and hyaluronan alongside the steroid-resistant pulmonary recruitment of effector inflammatory cells such as eosinophils and neutrophils (Parkinson et al, 2021).

We have previously described differences in both the steady state ECM and the kinetics of allergic remodelling and immune cell recruitment between in-bred BALB/c and C57BL/6 strains of mice (Parkinson et al, 2021). Furthermore, BALB/c and C57BL/6 mice have been shown to have markedly different cytokine responses and immune cell recruitment (Morokata et al, 1999), and respond differently to methacholine-induced AHR during models of AAI (Gueders et al, 2009). Regardless, it is unknown whether any of these strain differences could be explained by changing cell-cell or cell-matrix interactions within the healthy versus diseased lung environment.

Interrogating the spatial distribution of cells and molecules within tissues has traditionally relied on immunofluorescence (IF) staining, which is often limited to four or five targets due to spectral overlap between fluorophores (Hickey et al, 2022). The development of technologies such as imaging mass cytometry (IMC) (Giesen et al, 2014), cyclic immunofluorescence (IF) (Lin et al, 2016; Stack et al, 2014; Frei et al, 2016), and spatial transcriptomics (Asp et al, 2020), now allows characterisation of the tissue environment in much greater detail. IMC and other highly multiplexed tissue imaging techniques can simultaneously detect 40 or more markers on the same tissue section. Complementary techniques such as multiparameter flow cytometry and single-cell ribonucleic acid (RNA) sequencing have further deepened our understanding of cellular responses (Kolodziejczyk et al, 2015), albeit at the cost of spatial information. Multiplexed imaging has been applied to studying cell-cell interactions within the tumour microenvironment (Giesen et al, 2014; Sheng et al, 2022; Ravi et al, 2022; Veenstra et al, 2023; Haley et al, 2024), and recently, the cytokine milieu across different tumour regions (Hoch et al, 2022). Despite these advances, the ECM is often overlooked, relatively few studies have incorporated in-depth ECM classification, and fewer still have integrated ECM markers with cellular analysis within the same section.

We therefore aimed to generate an exploratory, spatially resolved atlas of the lung immunomatrix at a chronic timepoint of allergic inflammation in both BALB/c and C57BL/6 strains to also serve as a basis for future mechanistic studies. To encompass both cellular and ECM analysis within the same section, IMC was employed with a new approach to integrate ECM analysis with existing multiplex image analysis pipelines. During AAI, we were able to identify distinct matrix environments that associated with observable tissue structures, including airway-associated inflammatory foci. The immune cell recruitment observed in DRA-induced AAI was localised within distinct lung adventitial or alveolar niches, and these correlated with specific matrix environments in BALB/c and C57BL/6 mice. Furthermore, expansion of cells within the remodelled subepithelial space during AAI correlated with enhanced interactions between CD11b^+^ cells and stromal populations. Complementary analysis of 3D lung slices and tissue sections using immunofluorescent staining confirmed macrophages as a key immune population immediately surrounding remodelled airways. Together, these data suggest that inflammatory cell distribution is markedly different between BALB/c and C57BL/6 mice and immune-stromal cell networks in the subepithelial region may form an integral network that drives and maintains changes to the extracellular matrix environment during allergic airway pathology.

## Results

### Distinct epithelial, stromal, and immune niches exist in the naïve and allergic lung

**Figure 1:**
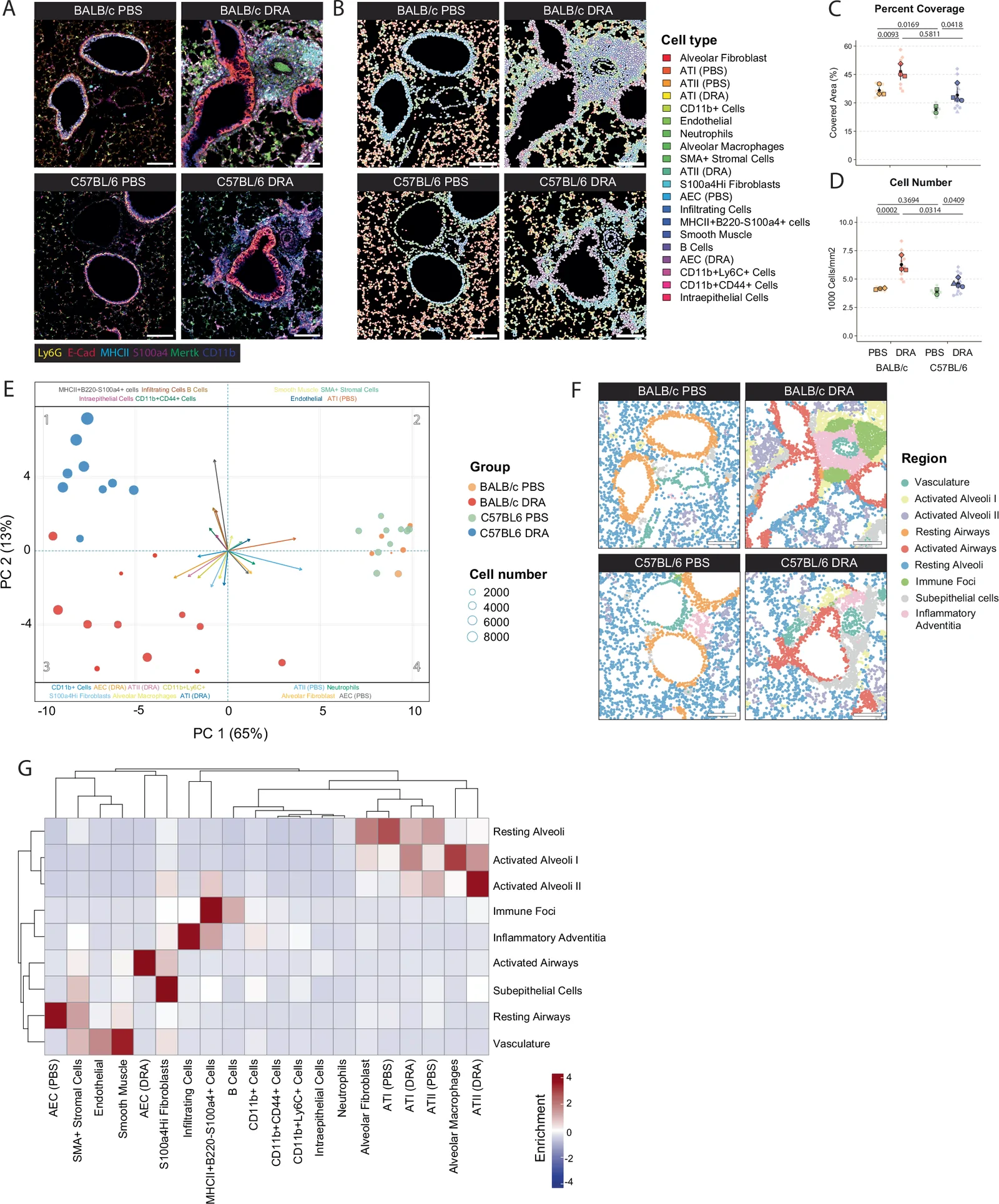
Distinct epithelial, stromal, and immune niches exist in the naïve and allergic lung. **A** Representative IMC staining across C57BL/6 or BALB/c mice treated for 8 weeks with PBS or DRA, showing DRA-driven inflammation and cell activation. **B** DeepCell was used to generate cell masks from nuclear and cellular staining. From this a single-cell expression profile for each masked cell was quantified. Cells were grouped using dimensionality reduction and SNN clustering followed by manual separation based on specific cellular expression data. **C** Percentage of each ROI covered by the cell mask and **D** total number of cells within each ROI. **E** Variation across ROIs was visualised using centred log-ratios (CLR) on cluster proportions across samples, projected onto a PCA. Arrows represent loadings and are colour-coded to cell type. **F** lisaClust-defined tissue regions projected onto DeepCell-generated cell masks. **G** Contribution of specific cell clusters to lisaClust regions. Scale represents proportions of cell types making up each tissue region, scaled and centred relative to the row. For **A**, **B**, **F** the same representative sample from each group is shown; scale bar 130 μm. For **E** each separate ROI is plotted. For **C** and **D** smaller background data points represent individual ROIs, larger foreground points show the average per animal, with black error bars showing the 95% confidence interval around the mean (black filled circle). *n* = 2–4 animals with three corresponding ROIs per sample; mean and error bars are calculated from all ROIs. Statistical significance was calculated with linear mixed-effects Models using PROC MIXED in SAS, with *P* values shown on the graph, see methods for full details. Source data are available online for this figure.

**Figure EV1:**
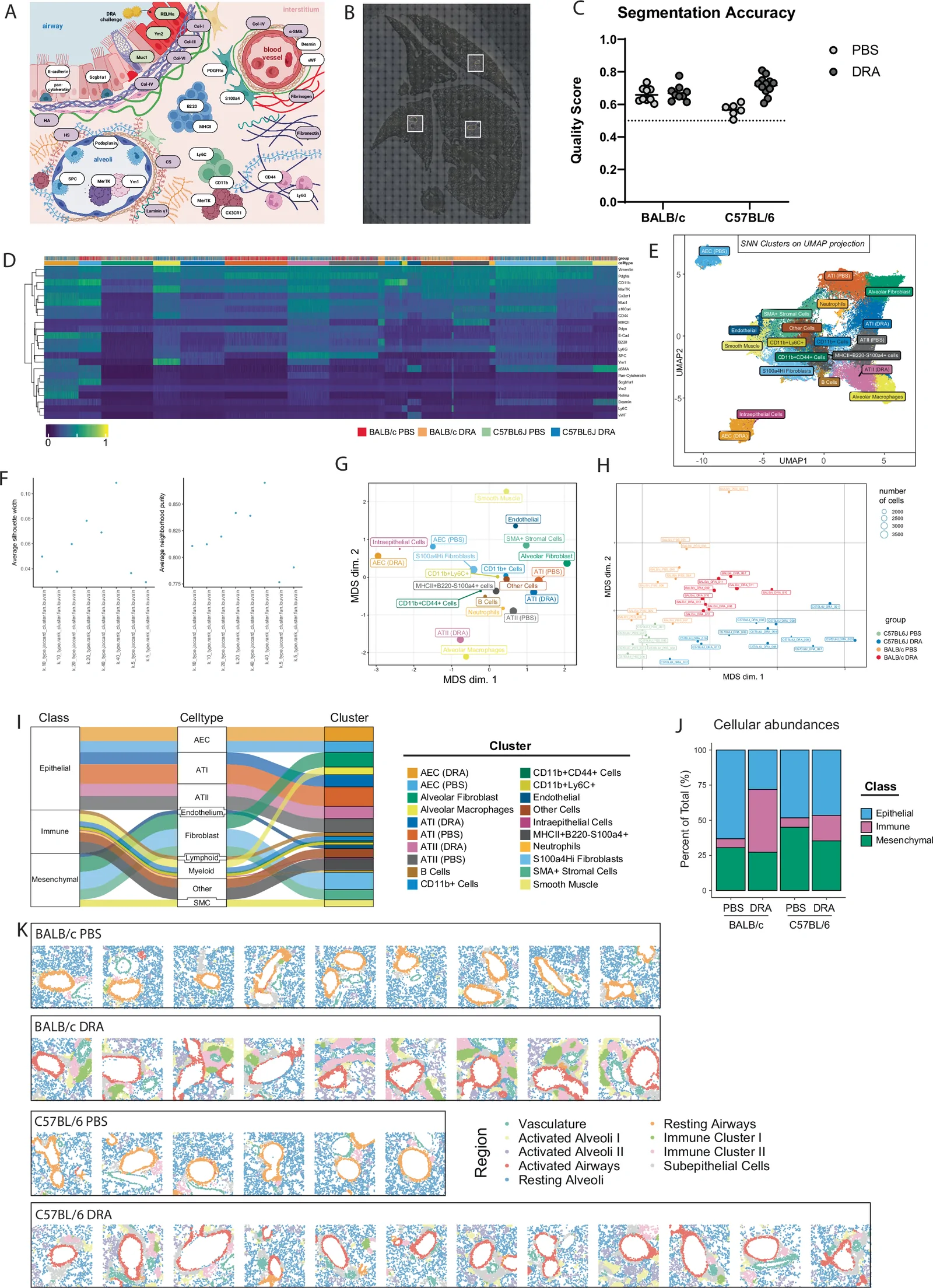
IMC allows identification of different cell populations in the lung. (**A**) IMC panel antibodies designed to cover a wide range of cell populations and extracellular matrix components found across sub-structures within the lung, such as airways, vessels and alveoli. (**B**) Representative image of an IMC-stained section from a BALB/c PBS-treated animal with the three separate ROIs highlighted (white boxes). Areas were chosen to include at least one small-medium airway and associated structures. Cell segmentation was performed with DeepCell and (**C**) segmentation accuracy was assessed with the CellSegmentationEvaluator pipeline (GitHub), dashed black line shows an equivalent “good” segmentation from a mixed dataset (Chen and Murphy, 2023) with circles representing individual ROIs. Cells were then clustered and annotated based on marker expression. (**D**) Heatmap of a subset of 20,000 cells showing the arcsin-transformed mean expression per cell of the different markers. (**E**) UMAP projection showing cells coloured by their SNN cluster and labelled with their manual annotation. (**F**) Optimal clustering parameters were determined using average silhouette width and neighbourhood purity generated within the Bluster package in R. Manual investigation of K = 40 clusters showed no identifiable improvement over K = 20, so K = 20 was used. (**G**) Multiple dimension scaling (MDS) of cell populations based on average marker expression. (**H**) MDS reduction using the proportions of different cell clusters per ROI for all mice. (**I**) Schematic showing the categorisation and relationship between different levels of the cell populations identified in all mice. (**J**) To broadly characterise the response across mice, the abundance of cells in defined classes from (**I**) were quantified. (**K**) LisaClust regions across all individual ROIs in the dataset. Points represent individual ROIs with the black horizontal bars showing the mean for all ROIs in that group; *n* = 2–4 animals with three corresponding ROIs per sample (**C**) and bars represent the average for that population across all ROIs of that group (**J**).

We have previously demonstrated that chronic intranasal administration of an allergen cocktail containing house dust mite, ragweed and aspergillus extracts to either BALB/c or C57BL/6 mice causes broad airway inflammation and remodelling (Parkinson et al, 2021). To better characterise how the spatial organisation of the lung environment was altered during allergic airway inflammation, we performed IMC on lung sections from PBS and DRA-challenged BALB/c and C57BL/6 mice. Whilst there is an extensive literature examining pulmonary cellular changes during AAI (Kay et al, 2004; Ray & Kolls, 2017; Moore et al, 2014; Ordoñez et al, 2000; Pham et al, 2017; Robinson et al, 1993; Filley et al, 1982; Broide et al, 1991), very little work has examined this cellular composition in relation to the ECM. With this in mind, we designed a panel of 32 antibodies, including 19 population markers, 3 activation markers, and 10 ECM markers (Figs. 1A and EV1A). To account for heterogeneity across the lung, three regions of interest (ROI) per mouse were imaged across different lung lobes (Fig. EV1B). All ROIs encompassed at least one small-medium airway, associated blood vessels, and the immediate alveolar parenchyma.

Processing of raw IMC data was performed using the Steinbock pipeline (Windhager et al, 2023). Cell masks were generated using DeepCell (Greenwald et al, 2022) (Fig. 1B) and validated against a heterogeneous example dataset using CellSegmentationEvaluator (Chen and Murphy, 2023), showing a high segmentation accuracy (Fig. EV1C). Relative cell density, measured as the percentage of ROI covered by the cell mask, was equivalently increased in both strains during AAI (Fig. 1C). Cell density was consistent with increased total cell numbers (Fig. 1D), aligning with previously published flow cytometry analysis of these samples (Parkinson et al, 2021). However, IMC analysis revealed that the allergen-induced cellular increase was significantly greater in BALB/c compared to C57BL/6 mice (Fig. 1D), suggesting strain-dependent differences in cellular recruitment to these spatial regions. To dissect the identity of recruited cells, shared nearest neighbour clustering (Ertöz et al, 2003) followed by manual separation based on canonical marker expression identified 20 distinct cell clusters (Fig. EV1D,E). To ensure accuracy, optimal clustering was assessed (Fig. EV1F) and harmony normalisation applied to account for batch variation (Korsunsky et al, 2019) (see Methods). Clusters were annotated by marker expression and spatial localisation (Figs. 1A and EV1D), with similar cell types (Fig. EV1G) or treatment groups (Fig. EV1H) localised closely together following multidimensional scaling. Analysis identified key cellular populations potentially driving allergic responses, including CD11b^+^ immune cells and S100a4^+^ fibroblast populations, among others (Fig. 1E). To simplify downstream analysis, clusters were grouped into broader cell types and classes based on lineage and function (Fig. EV1I). Quantification of classes revealed a shift from predominantly epithelial cell composition in PBS-treated animals to an increased proportion of immune cells following DRA administration, most pronounced in BALB/c mice (Fig. EV1J).

Given that we lacked markers for certain cellular populations (e.g., T cells) and observed variance in expression levels for others (e.g., PDGFRα expression in fibroblasts), we asked whether organised tissue regions could be defined based on their constituent cell types rather than individual marker expression alone. Applying the lisaClust algorithm (Patrick et al, 2023) to all merged ROI data identified nine spatially distinct tissue regions annotated based on their key constituent cell types, localisation and cell interaction patterns (Figs. 1F,G and EV1K). Regions corresponding to vasculature, airway epithelium, and immune foci were clearly distinguishable, and several regions, including airway and alveolar epithelium, were separated between PBS and DRA conditions, reflecting different activation states (Fig. 1G). Regions associated with inflammatory cell recruitment were expanded in DRA-challenged animals, with expansion again more pronounced in BALB/c mice (Fig. 1F).

### Lung regions are associated with distinct ECM environments that change during AAI

**Figure 2:**
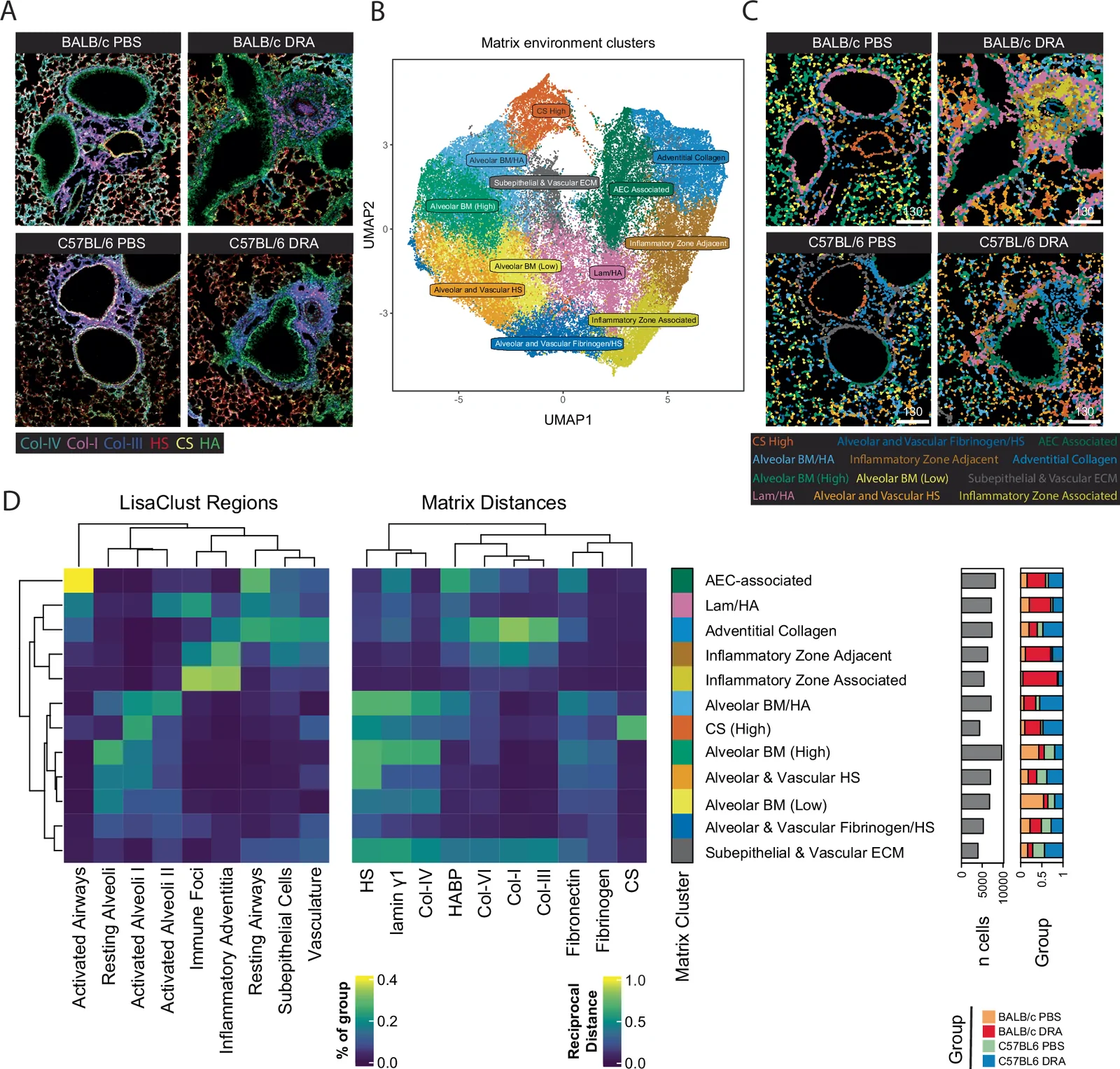
Lung regions are associated with distinct ECM environments, which change during AAI. In order to determine if specific matrix components were associated with specific populations or treatments, we aimed to characterise matrix environments of the separate cell clusters. (**A**) Staining of select ECM molecules on representative ROIs. Matrix distances were calculated as described in the methods and then (**B**) SNN clustering defined distinct matrix environments, which were projected on UMAP dimensionality reduction. (**C**) Projection of defined matrix environments onto DeepCell-generated cell masks for representative images. Scale bar 130 μm. (**D**) Heatmap visualising enrichment of specific matrix environments in contingency tables. The left panel shows the percentage of cells in LisaClust regions associated with the specific matrix environment. The right panel shows the average distance to labelled matrix components (higher is closer). Grey bars show the total number of cells assigned to different matrix environments, and stacked bars show the relative contribution to different genotypes and treatments. Values are plotted as scaled and centred reciprocals (**D**). (CS chondroitin sulphate, HS heparan sulphate, HA hyaluronan). Images from (**A**) have been reused in Fig. EV4A. Source data are available online for this figure.

**Figure EV2:**
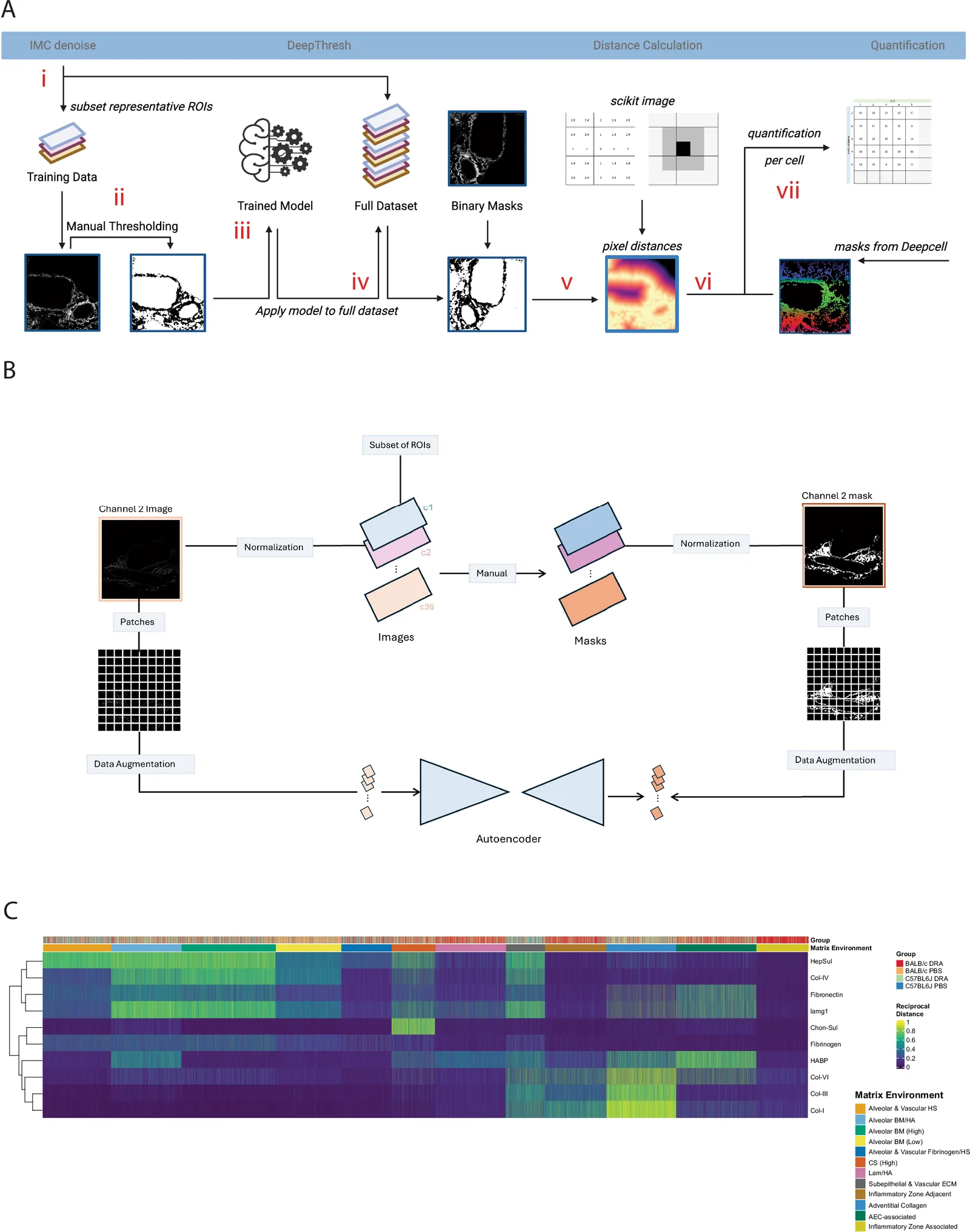
DeepThresh for analysis of matrix staining in IMC data. (**A**) Schematic showing the DeepThresh pipeline for matrix distance quantification. (**i**) Raw stacked tiff files were exported from the IMCdenoise pipeline and split into two groups. (**ii**) One group consisting of a representative sample from each biological group was manually thresholded to generate a binary image showing positive staining as 1 (foreground) and negative staining as 0 (background). (**iii**) A random forest classifier was trained using this data to identify positive regions. (**iv**) The trained classifier was applied to the full dataset to threshold positive staining. (**v**) Thresholded images were used to calculate pixel distances from positive staining using the scikit-image package and images were appended to the original stacked tiff files. (**vi**) Masks were generated with DeepCell using the IMCdenoise data and used to quantify the expression of cell markers, including distance values per cell mask. Data were imported into the normal Steinbock pipeline for further analysis. (**B**) Detailed description of the methodology for thresholding images using DeepThresh. All regions of interest (ROIs) were enhanced using IMCDenoise (Lu et al, 2023). Six ROIs were manually thresholded for use in a semi-automated thresholding approach we refer to as DeepThresh. Four images were used for training, one for validation and one for testing. DeepThresh was built using PyTorch2 (version 2.3) and Pytorch Segmentation Models3. The architecture mimics that of U-Net4 with a ResNet152 encoder5. Adam6 optimiser was applied with a learning rate of 0.0001. All models were trained for 50 epochs. (**C**) Heatmap showing matrix distances across classified matrix environments for all ECM markers evaluated. The scale shows a reciprocal transformation.

Having characterised distinct cell types and tissue regions within the lung, we next asked how these niches were related to the localisation of ECM components, which were broadly altered in expression and/or organisation during AAI (Fig. 2A). As ECM molecules are extracellular and therefore poorly represented by cell mask-based analysis, we developed a complementary approach to classify each cell’s matrix environment based on its proximity to specific ECM components. Briefly, ECM markers were segmented by applying an intensity threshold to distinguish stained from unstained regions. As traditional thresholding methods, such as Otsu’s (Otsu, 1979) proved sensitive to staining variation between ROIs, we developed a novel deep-learning thresholding algorithm, Deepthresh was trained on expert-annotated images to generate robust binary segmentation masks across all ROIs (Fig. EV2A,B and Methods). Euclidean distance calculation from each cell to segmented ECM components were then used as input for SNN clustering, generating twelve distinct lung matrix environments (Fig. 2B).

These matrix environments displayed clear spatial patterning that correlated with known structures including airways, vessels, or alveolar epithelium (Fig. 2C). Cellular matrix environments were annotated according to their nearest ECM components (Fig. EV2C) and spatial localisation within the lung (Fig. 2C). Correlating matrix environments with lisaClust tissue regions (Figs. 2D, 1G) revealed two major compartments within the lung (Fig. 2D). Firstly, the alveolar parenchyma was associated with heparan sulphate, laminin gamma-1, type-IV collagen, and fibrinogen. Secondly, the adventitial cuff surrounding the airways and blood vessels was enriched for type-I, III, VI collagens and hyaluronan (Fig. 2D). Beyond these structural associations, specific cellular matrix environments were differentially associated with health or allergic pathology across mouse strains; notably alveolar basement membrane (BM) clusters were prominent in PBS-treated mice whilst airway epithelial cell (AEC)-associated matrix environments expanded in DRA-treated mice (Fig. 2D). Together, this approach provides a spatially resolved classification of the lung matrix landscape and a framework for examining how cell-matrix interactions are altered during AAI and pathways that may contribute to long-term lung pathology.

### Infiltrating immune cells localise in patches around the adventitial cuff

**Figure EV3:**
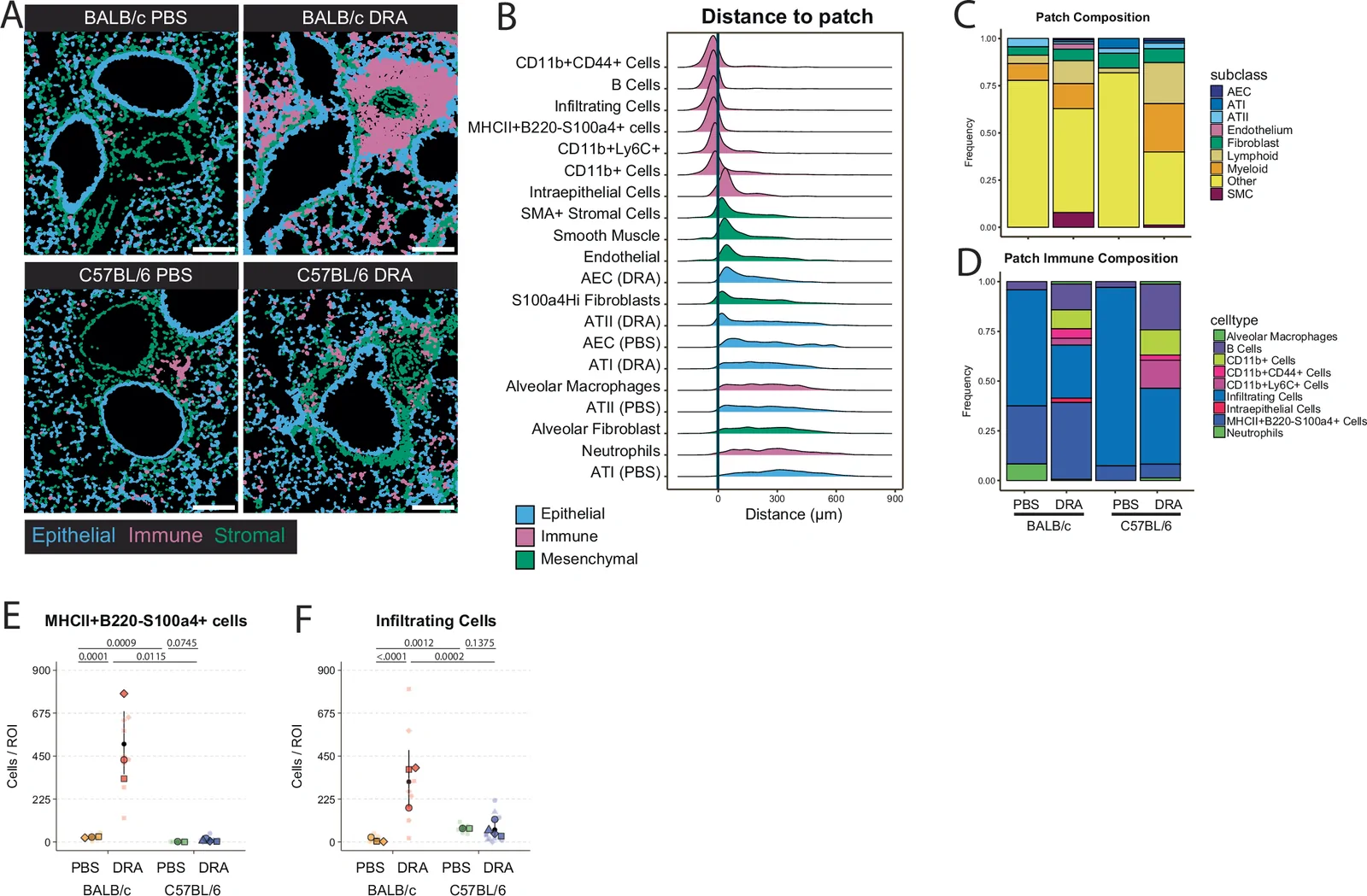
Spatial cellular composition of the lung during allergic airway pathology. (**A**) Representative images showing the distribution of different cell classes defined in Fig. 1. (**B**) The mean Euclidean distance (μm) of previously defined SNN cell clusters from their nearest patch. The vertical black line represents 0, the external border of the cell patch. Bars are coloured according to cell class. (**C**) Mean frequency of cell types found across all patches across treatment and mouse strain and (**D**) mean frequency of specific SNN cell clusters within the immune cell class. Quantification of mean cells per ROI of (**E**) MHCII^+^B220^-^S100a4^+^ cells and (**F**) infiltrating cells per ROI. (**A**) The same representative sample from each group are shown; scale bars 130 μm. For (**E**, **F**), data points represent individual ROIs with error bars showing the 95% confidence intervals around the mean (black filled circle). *n* = 2–4 animals per group corresponding to 6–12 ROIs per group; mean and error bars are calculated from all ROIs. Statistical significance was calculated with linear mixed-effects models using PROC MIXED in SAS, with *P* values shown on the graph; see Methods for full details.

**Figure 3:**
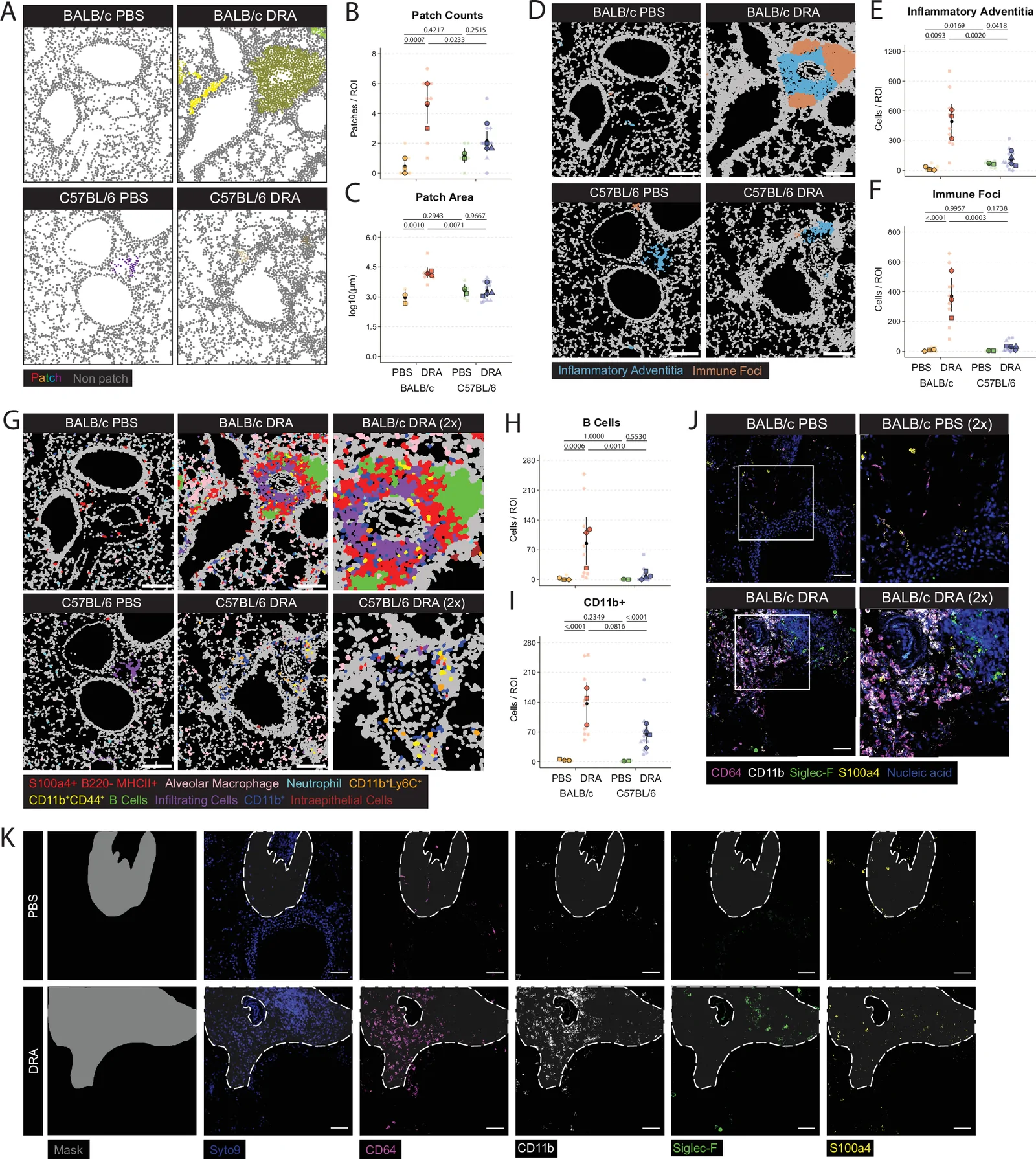
Allergic infiltrating immune cells localise in patches around the adventitial cuff. To quantify if cells were accumulating together, “patches” were calculated for the “immune” cell class using the patchdetection() algorithm. (**A**) Representative images of separate immune patches in unique colours for the four different groups. Quantification of (**B**) the mean number and (**C**) the mean area of the patches per ROI. To classify the adventitial immune response, (**D**) lisaClust regions associated with the adventitial cuff were projected onto DeepCell masks. Quantification of the number of cells per ROI within the (**E**) inflammatory adventitia and **F**) immune foci lisaClust regions. (**G**) Projection of previously characterised “immune” populations onto the DeepCell masks and quantification of numbers of (**H**) B Cells and (**I**) CD11b^+^ across ROIs. (**J**) To further investigate cell populations present in the adventitial cuff, 3D PCLS were stained with immune and stromal markers. (**K**) The arivis cloud was used to segment ROIs and create a mask (white dashed line) where cell populations were present in different regions of the adventitial cuff. For (**A**, **D**, **G**) the same representative sample from each group are shown; scale bars 130 μm. For (**J**, **K**), all stained samples are shown; scale bars 50 μm. For (**B**,** C**,** E**,** F**, **H**, **I**), smaller background data points represent individual ROIs, larger points show the average per animal, with black error bars showing the 95% confidence intervals around the mean (black filled circle). *n* = 2–4 animals with three corresponding ROIs per sample; mean and error bars are calculated from all ROIs. Statistical significance was calculated with linear mixed-effects models using PROC MIXED in SAS, with *P* values shown on the graph; see Methods for full details. Source data are available online for this figure.

Having identified tissue niches and cellular matrix environments in the lung, we next assessed whether these changed following AAI in both C57BL/6 and BALB/c mice. DRA-induced inflammation increased the overall proportion of immune cells (Fig. EV1J), and these cells were mostly enriched in discrete patches within the adventitial cuff, particularly in BALB/c DRA-treated animals (Fig. EV3A). To quantify the size and composition of these patches, we applied the patchDetection() function (Hoch et al, 2022), which identified contiguous immune cell patches around the adventitial cuff that surrounds the vasculature, in both PBS- and DRA-treated animals (Fig. 3A). Patches were more prevalent (Fig. 3B) and larger (Fig. 3C) in BALB/c DRA-treated mice compared to C57BL/6. Detected patches consisted predominantly of immune cells (excluding intraepithelial cells, alveolar macrophages, and neutrophils) (Fig. B–D) and spatially corresponded to lisaClust defined inflammatory adventitia and immune foci regions (Fig. 3D). Both tissue regions were also specifically increased in BALB/c DRA-treated animals (Fig. 3E,F). The immune foci lisaClust region was enriched for B cells (Fig. 1G), which formed clusters that resembled induced bronchoalveolar lymphoid tissue (iBALT) (Hwang et al, 2016) (Fig. 3F). Total B cell numbers were increased in allergic BALB/c mice (Fig. 3G,H). In contrast, the inflammatory adventitia was enriched for infiltrating cells and CD11b^+^ cells (Figs. 1G and 3G,I). CD11b^+^ cells increased in both strains following DRA-treatment (Fig. 3I), whereas MHCII^+^B220^-^S100a4^+^ cells increased only in BALB/c mice (Fig. EV3E).

Infiltrating cells were elevated (Fig. EV3F) following DRA-treatment in both the immune foci and inflammatory adventitia lisaClust regions (Fig. 1G). Due to limitations in IMC related to image resolution and marker availability, further cellular classification was not possible. Therefore, 3D precision-cut lung slices (PCLS) from BALB/c mice were stained with fluorescently conjugated antibodies and imaged by confocal microscopy. After four weeks of DRA administration, similar immune cell patches formed around the adventitial cuff (Fig. 3J), consistent with the IMC dataset (Fig. 3G). Semantic segmentation of the adventitial cuff (Fig. 3K) revealed a clear dichotomy within the adventitial cuff. The area adjacent to the blood vessel was enriched for CD11b^+^CD64^+^ monocyte-derived macrophages (Fig. 3J), resembling the lisaClust inflammatory adventitia region. In contrast, the more distal nuclei-dense area (Fig. 3J) resembled the immune foci region. Monocyte-derived macrophages were largely excluded from this dense iBALT-like region, although SiglecF^+^ cells, likely eosinophils, were present (Fig. 3J). Low numbers of S100a4^+^ cells were detected throughout the adventitial cuff, consistent with their enrichment in both regions from the IMC dataset (Fig. 1G). These data identify the adventitial cuff as a major site of inflammatory cell recruitment during AAI. The adventitial cuff itself also contains distinct locations with the iBALT-like tissue likely functioning as a reservoir for B cell activation, though the role of the myeloid-rich region is less clear.

### The adventitial ECM is differentially regulated across different mouse strains

**Figure 4:**
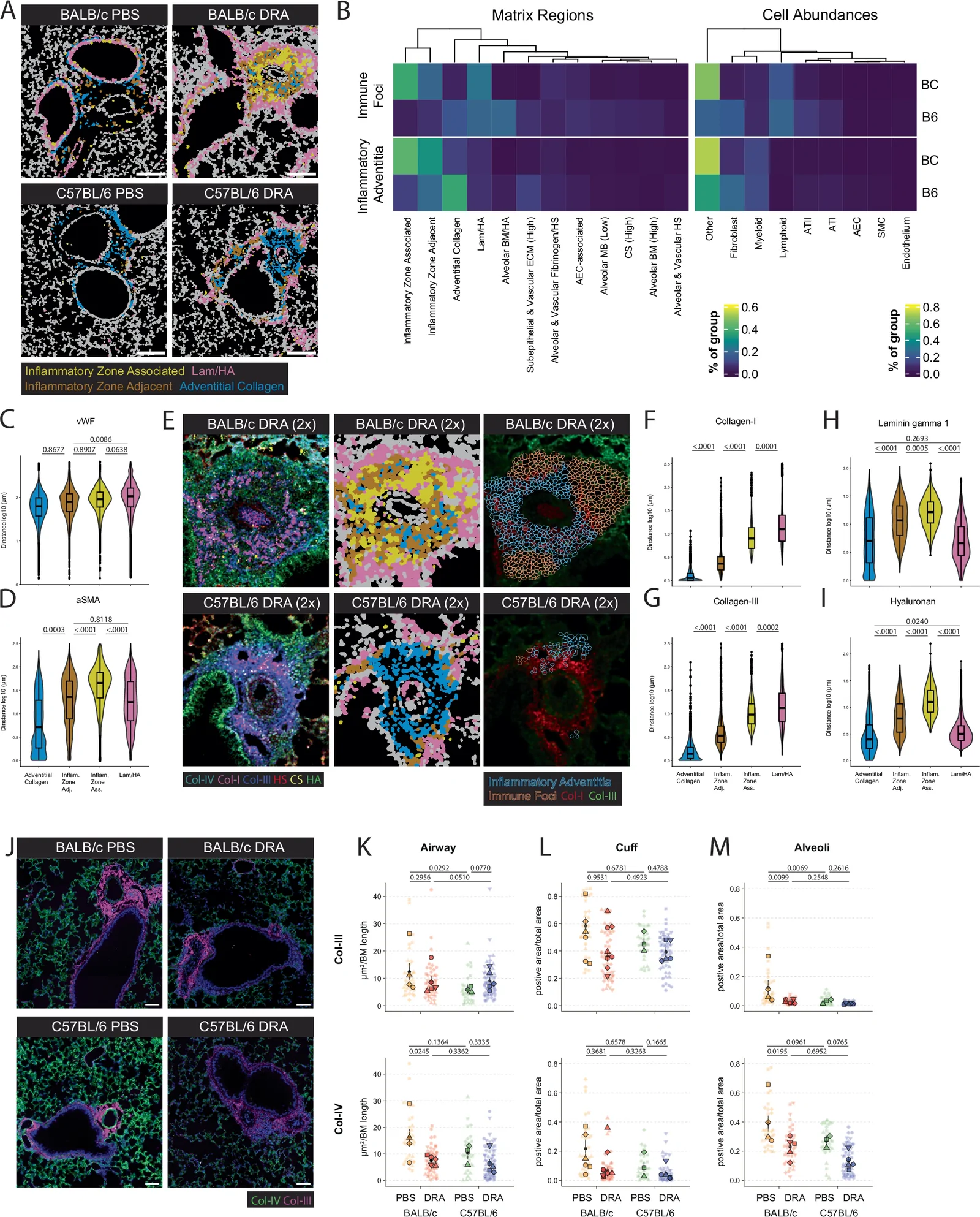
Spatial cellular profiles in the inflammatory adventitial cuff are associated with distinct matrix environments. (**A**) Matrix environments associated with inflammatory patches were projected onto DeepCell masks. (**B**) To visualise enrichment of specific matrix environments, contingency tables were generated and plotted via a heatmap. The left panel shows the percentage of cells in that region associated with the specific matrix environment. The right panel shows the percentage of cells in the region from previously defined cell classes. Scale represents the frequency of the region comprised of that matrix environment or cell type. Quantification of the distance of adventitial matrix environments from tissue structures, specifically (**C**) vWF, marking the vasculature and (**D**) αSMA, marking the vasculature and airways. (**E**) Correlation of specific ECM staining around the adventitial cuff (left panel), associated matrix environments from (**A**) (centre panel), and DeepCell masks coloured by their lisaClust region overlaid onto type-I and type-III collagen staining (right panel). Quantification of the distance of adventitial matrix environments to (**F**) type-I collagen, (**G**) type-III collagen, (**H**) laminin γ1, and (**I**) hyaluronan. (**J**) Representative images showing collagen type-III and type-IV in the lungs of BALB/c or C57BL/6 mice treated with PBS or DRA for 8 weeks. Quantification of area collagen type-III or type-IV in the (**K**) airway, (**L**) cuff or (**M**) alveoli. Airway measurements were normalised to the basement membrane length and cuff, and alveoli were normalised to the total ROI area. For (**A**, **E**), the same representative sample from each group are shown; scale bars 130 μm. For (**E**), images are 2x magnification of the normal ROIs focused on the adventitial cuff. For (**C**,** D**,** F**,** G**,** H**, **I**) graphs are violin plots with overlaid boxplots. The thick central line represents the median, with the box showing the interquartile range and lines representing Q1 and Q4; outliers are shown as individual points. *n* = 2–4 animals per group corresponding to 6–12 ROIs per group; mean and error bars are calculated from all ROIs. Statistical significance was calculated with general linear models using PROC GLM in SAS, with *P* values shown on the graph; see Methods for full details. Source data are available online for this figure.

To investigate differences in the ECM surrounding the adventitial cuff during AAI and iBALT-like tissue formation, we analysed cellular matrix environments located near these structures (Fig. 2). Adventitial collagen, inflammatory zone adjacent, inflammatory zone associated, and Lam/HA environments (Fig. 2D) were all localised near the adventitial cuff (Fig. 4A). The adventitial collagen matrix environment was closely associated with type-I, III, and VI collagens (Fig. 2D) and was more prevalent in C57BL/6 DRA-treated animals (Fig. 4B) that showed no evidence of iBALT-like structures. In contrast, allergic BALB/c animals showed enrichment of the inflammatory zone adjacent and inflammatory zone- associated matrix environments (Fig. 4B). These inflammatory zone matrix environments exhibited an overall similar localisation pattern as the adventitial collagen environment, but with increased distances to each matrix component (Fig. 2D), indicating sparser matrix organisation. Increased sparsity correlated with greater distance to the vasculature (Fig. 4C,D) and airways (Fig. 4D). The Lam/HA environment differed in terms of proximity to the vasculature, showing closer proximity to αSMA-positive regions consistent with its location adjacent to the airways (Fig. 4A).

**Figure EV4:**
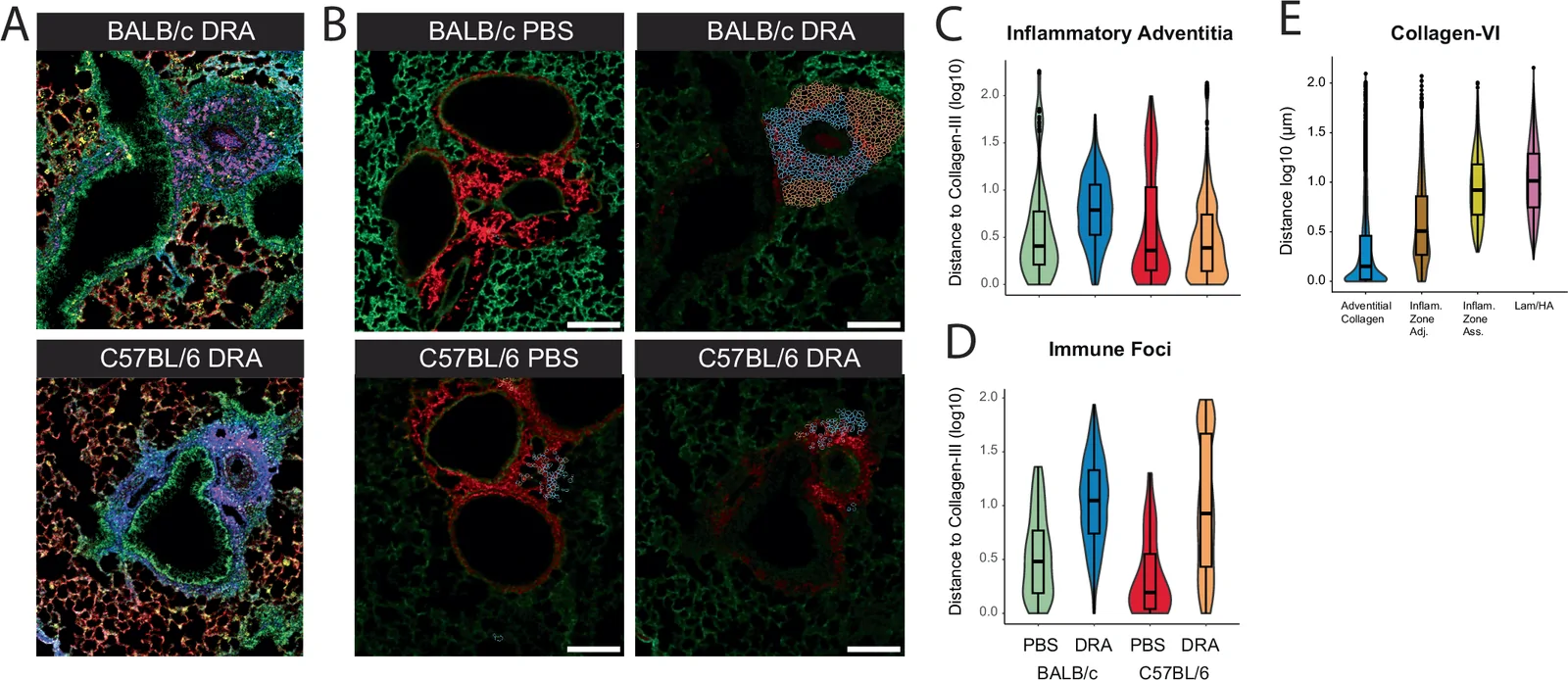
Immune cell localisation to ECM molecules within the adventitial cuff. (**A**) Representative images showing zoomed out ROIs shown in 4e (left). (**B**) Representative images showing zoomed out ROI from representative PBS-treated animals and DRA-treated animals, which are also shown in 4e (right). Quantification of the average Euclidean distance of each cell in the (**C**) inflammatory adventitia and (**D**) immune foci lisaClust region to type-III collagen. (**E**) Distance of adventitial matrix environments to collagen VI. For (**A**, **B**), the same representative sample from each group are shown and scale bars are equal to 130 μm. For (**C**–**E**), graphs are violin plots with overlaid boxplots. The thick central line represents the median with the box showing the interquartile range and lines representing Q1 and Q4, outliers are shown as individual points. *n* = 2–4 animals per group corresponding to 6–12 ROIs per group; mean and error bars are calculated from all ROIs. Statistical significance was calculated with general linear models using PROC GLM in SAS, with *P* values shown on the graph; see Methods for full details. HA hyaluronan. Images from (**A**) have been reused in Fig. 2A.

Sparser inflammatory zone matrix environments were associated with the more distal localisation of iBALT-like tissue, especially in DRA-treated animals (Figs. 4E and EV4C,D). Across these cellular matrix environments, there was a general increase in distance to type-I (Fig. 4F), type-III (Fig. 4G), and type-VI (Fig. EV4E) collagens. Conversely, a decreased distance to other ECM components, including laminin γ1 (Fig. 4H) and hyaluronan (Fig. 4I), was observed within the most distal Lam/HA environment, which was associated with iBALT-like structures (Fig. 4E). This suggests that a shift from a predominantly collagen matrix to a more laminin and hyaluronan-rich matrix is important for the function of this tissue.

To more broadly examine the changes in the collagen matrix in the lung during DRA, immunofluorescent staining of type-III and type-IV collagen was undertaken (Fig. 4J). Manual segmentation of the lung into the airway-associated, adventitial cuff, and alveoli allowed examination of changes in collagen subtypes across different lung regions. Airway type-III collagen increased only in C57BL/6 mice, whereas type-IV collagen was only decreased in BALB/c mice, as described previously (Parkinson et al, 2021) (Fig. 4K). No changes in collagen subtype were observed in the adventitial cuff, though this was likely due to being unable to disentangle the different sub-regions identified in our IMC data (Fig. 4L). The alveoli showed a decrease in type-III collagen in BALB/c mice specifically, but an equivalent decrease in type-IV collagen in both strains (Fig. 4M).

### DRA-induced allergy is associated with altered alveolar epithelial composition

**Figure 5:**
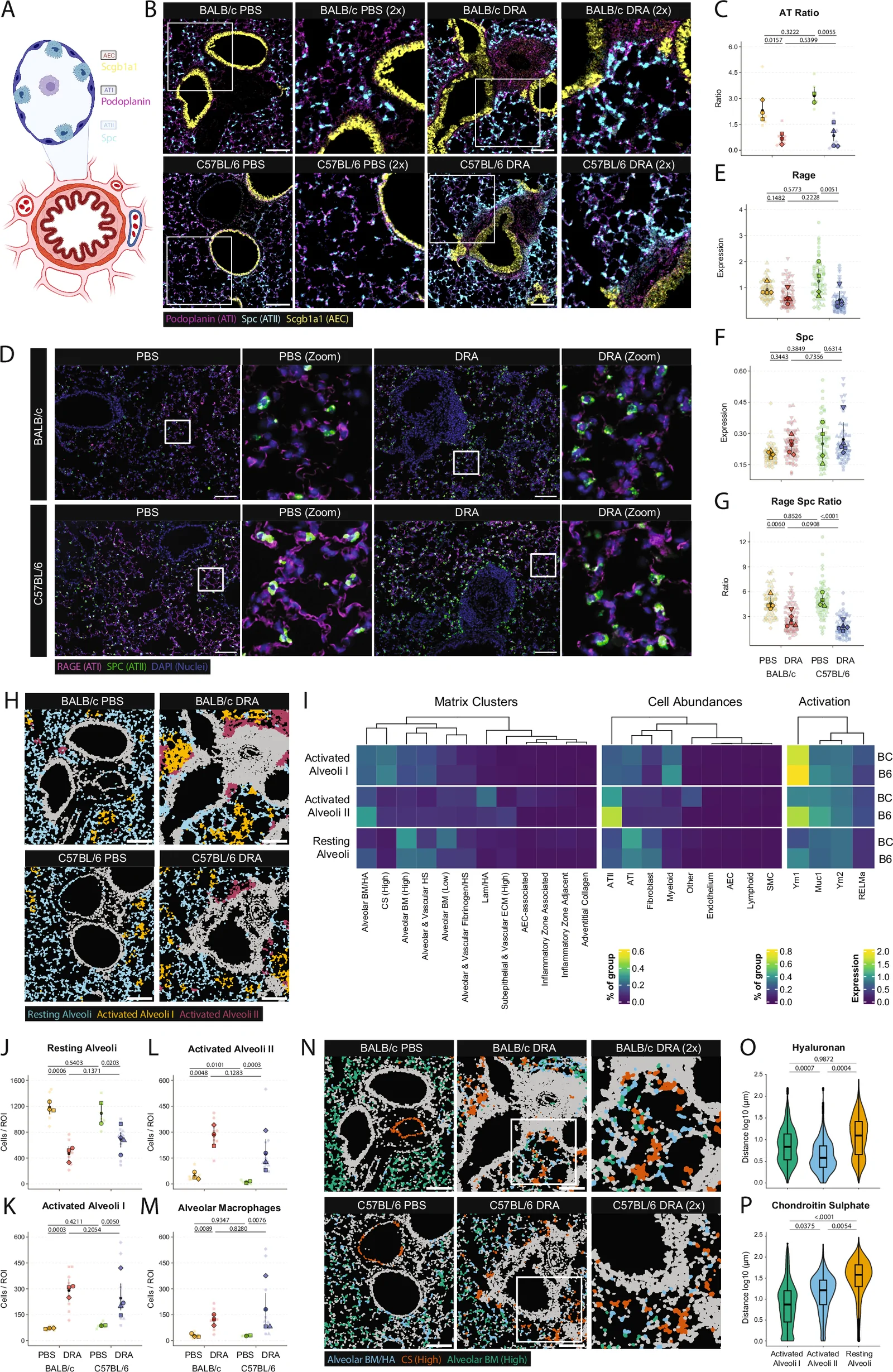
DRA-induced AAI is associated with a loss of ATI and expansion of ATII. (**A**) Representative epithelial cell populations and respective markers, in the lung. (**B**) Representative IMC images showing staining of epithelial cell markers across strains during DRA-challenge. (**C**) Mean ratio of ATI to ATII cells per ROI. (**D**) Representative images showing SPC and RAGE immunostaining to validate changes to the alveolar epithelium and associated quantification of the staining area of (**E**) RAGE, (**F**) SPC, and (**G**) the ratio of RAGE/SPC positive staining area. (**H**) Images of the alveolar lisaClust regions projected onto representative ROIs. (**I**) Contingency tables plotted via heatmap. The left panel shows the percentage of cells in that region associated with the specific matrix environment. The centre panel shows the percentage of cells in the region from previously defined cell classes. Both scales represent the frequency of the region comprised of that matrix environment or cell type. The right panel shows the expression of specific activation markers; the scale represents the scaled and centred mean expression. Associated quantification of the number of cells per ROI in the (**J**) resting alveoli, (**K**) activated alveoli I, and (**L**) activated alveoli II lisaClust regions. Quantification of the mean cell number per ROI of (**M**) alveolar macrophages across ROIs. (**N**) Visualisation of alveolar matrix environments projected onto DeepCell masks. Quantification of the distance to (**O**) hyaluronan and (**P**) chondroitin sulphate across the different alveolar lisaClust environments. For (**B**, **H**, **N**) the same representative sample from each group are shown; scale bars 130 μm. For (**D**), representative stained samples are shown, scale bars 100 μm. For (**C**,** E**–**G**,** J**–**M**), smaller background data points represent individual ROIs, larger points show the average per animal, with black error bars showing the 95% confidence intervals around the mean (black filled circle). (**B**, **C**, **K**–**N**) *n* = 2–4 animals with three corresponding ROIs per sample (**F**–**H**) *n* = 4–5 animals; mean and error bars are calculated from all ROIs. For (**O**, **P**) graphs are violin plots with overlaid boxplots. The thick central line represents the median, with the box showing the interquartile range and lines representing Q1 and Q4; outliers are shown as individual points. Statistical significance was calculated with linear mixed-effects models using PROC MIXED or general linear models using PROC GLM (for **O**, **P** only) in SAS, with *P* values shown on the graph; see Methods for full details. Figure (**A**) was made in BioRender. Source data are available online for this figure.

**Figure EV5:**
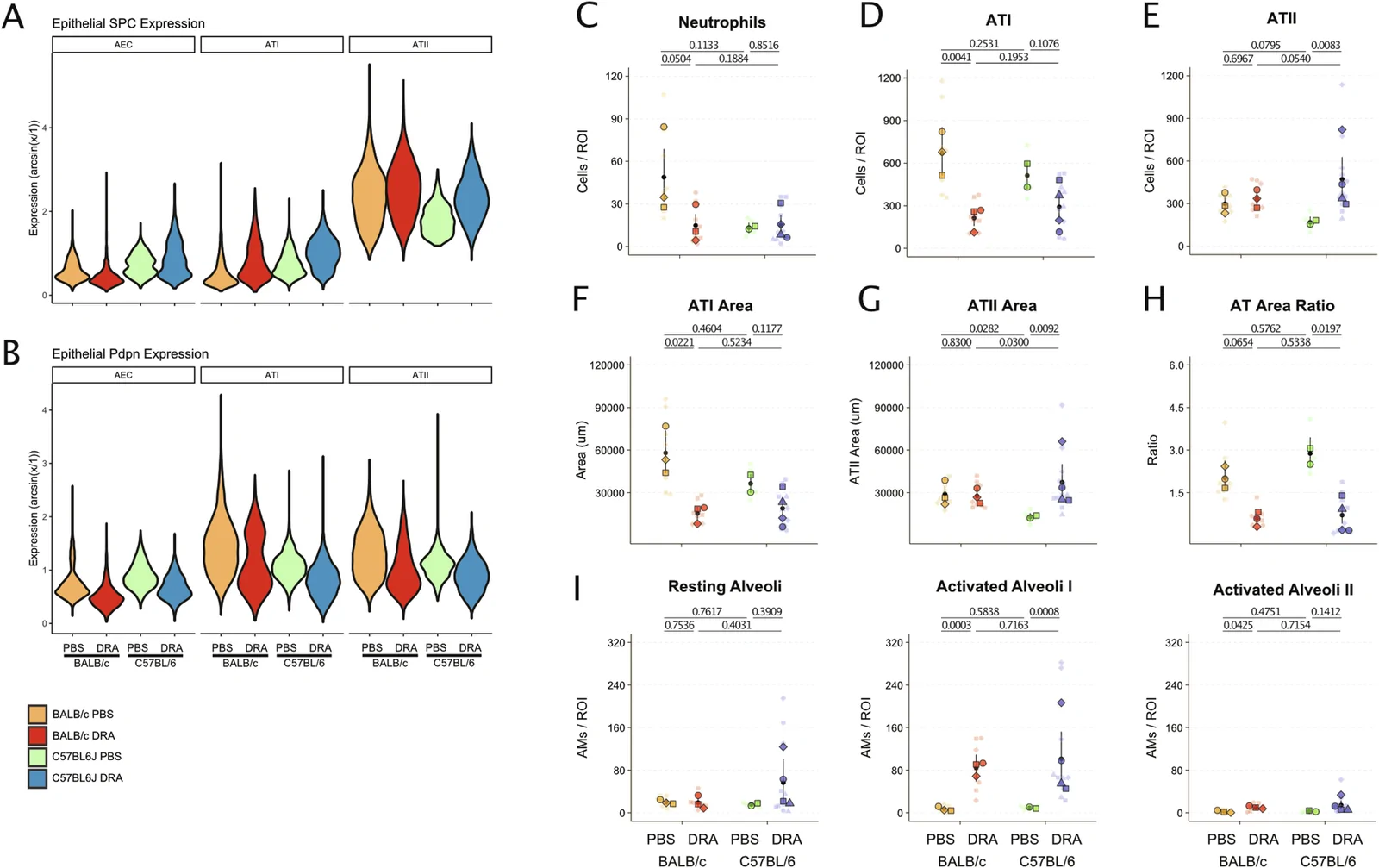
Phenotyping of the allergic lung epithelium. Lung epithelial cells express distinct markers, a violin plot showing (**A**) SPC expression and (**B**) Podoplanin (pdpn) expression in epithelial subsets. Quantification of the mean cell number per ROI of (**C**) Neutrophils, (**D**) alveolar type-I (ATI) cells, and (**E**) alveolar type-II (ATII) cells. Quantification of the mean area of (**F**) ATI and (**G**) ATII cells per ROI and (**H**) the ratio of this ATI and ATII area. (**I**) Quantification of the number of alveolar macrophages across different alveolar tissue regions. For (**A**, **B**), the expression of all cells in labelled populations per group are shown in a violin plot. For (**C**–**I**), smaller background data points represent individual ROIs, larger points show the average per animals, with black error bars showing the 95% confidence intervals around the mean (black filled circle). *n* = 2–4 animals with three corresponding ROIs per sample; mean and error bars are calculated from all ROIs. Statistical significance was calculated with linear mixed-effects models using PROC MIXED in SAS, with *P* values shown on the graph; see Methods for full details.

Distinct localisation of pulmonary immune cells was observed, with most infiltrating cells residing within the adventitial cuff. In contrast, alveolar macrophages and neutrophils are localised predominantly within the alveolar parenchyma, which is not traditionally considered a primary site of allergic remodelling. Three broad epithelial cell populations are present in the lung: AEC, alveolar type-1 cells (ATI), and alveolar type-II cells (ATII) (Fig. 5A), identified by expression of Uteroglobin (Scgb1a1), Surfactant protein C (Spc), and Podoplanin (Pdpn), respectively (Figs. 5A and EV5A,B).

Allergens drive tissue damage (Noureddine et al, 2022; Yuan et al, 2018), but no allergen-induced increase in neutrophils was observed spatially within the tissue regions examined for either strain (Fig. EV5C). However, injury-induced loss of ATIs followed by ATII proliferation to replace the ATI compartment, is a recognised feature of lung damage and repair (Jansing et al, 2017). Following DRA challenge, ATI and ATII populations were differentially altered between BALB/c and C57BL/6 mice. ATI cells were reduced in BALB/c but not C57BL/6 mice (Figs. 5B and EV5D), whereas ATII cells were increased only in C57BL/6 mice (Figs. 5B and EV5E). Despite these strain-specific differences, the ATI:ATII ratio was significantly decreased in both strains from ~3:1 in PBS-treated controls to ~1:1 in DRA-treated animals (Fig. 5C), indicating substantial reorganisation of the alveolar epithelium during AAI. ATII cells are required to markedly expand their surface area during injury and replacement of ATI cells (Jansing et al, 2017). However, the same pattern was observed for the total area covered by these cell populations in our IMC dataset (Fig. EV5F–H), though accurate quantification may be hindered by limitations of the technology.

To validate these findings, immunofluorescence staining was performed using SPC to label ATII cells and receptor for advanced glycation endproducts (RAGE) as a more specific marker of ATI cells (Fig. 5D), selected based on a single-cell RNA sequencing dataset (Curras-Alonso et al, 2023), RAGE^+^ area was significantly reduced in DRA compared to PBS-treated BALB/c but not C57BL/6 animals (Fig. 5E). SPC^+^ area was not significantly increased in either strain following DRA administration (Fig. 5F). However, the ratio of RAGE^+^ to SPC^+^ area was significantly reduced following DRA-treatment in both strains (Fig. 5G), supporting a relative decrease in ATI after allergen exposure.

Given the variability observed in single-marker staining, we returned to lisaClust defined tissue regions (Fig. 1). The alveolar parenchyma was subdivided into three distinct regions: resting alveoli, activated alveoli I and activated alveoli II (Fig. 5H,I). Resting alveoli were enriched for ATI cells (Fig. 5I) and predominated in PBS-treated animals of both strains (Fig. 5J). Activated alveoli I were enriched for ATII cells (Fig. 5I) and increased following allergen challenge in both strains (Fig. 5K). Activated alveoli II were also increased in DRA-treated animals in both strains (Fig. 5L) and displayed a more balanced ATI and ATII composition alongside the presence of myeloid cells (Fig. 5I). Alveolar macrophages, the dominant myeloid population in the alveoli, were increased in both strains following DRA treatment (Fig. 5M) and were particularly enriched within the activated alveoli I region (Figs. 5I and EV5I).

To assess whether ECM composition was associated with epithelial cell alterations, contingency analyses were performed across alveolar tissue regions. Heparan sulphate, laminin γ 1, and type-IV collagen were core components of the alveolar matrix environments (Figs. 2D and 5N). Resting alveoli were enriched for ‘alveolar BM high’ and ‘alveolar BM low’ cellular matrix environments (Fig. 5I) containing heparan sulphate, laminin and type-IV collagen, but lacking HA and CS (Fig. 2D). Following DRA administration, alveolar matrix environments shifted towards alveolar BM/HA and CS (high) regions (Fig. 5N), correlating with reduced distances between alveolar cells and both HA (Fig. 5O) and CS (Fig. 5P). These changes were observed in both mouse strains, although they were more widespread in BALB/c mice. Increased CS expression within the CS (High) matrix environments was associated with activated alveoli I regions and increased numbers of Ym1^+^ alveolar macrophages (Nio et al, 2004; Draijer et al, 2022) (Figs. 5I,M,N and EV5H). Collectively, these findings suggest that increased HA and CS in the alveolar parenchyma accompany alterations in alveolar epithelial cells during allergic inflammation, remodelling and tissue repair.

### Airway epithelial cell activation is equivalent between strains

**Figure 6:**
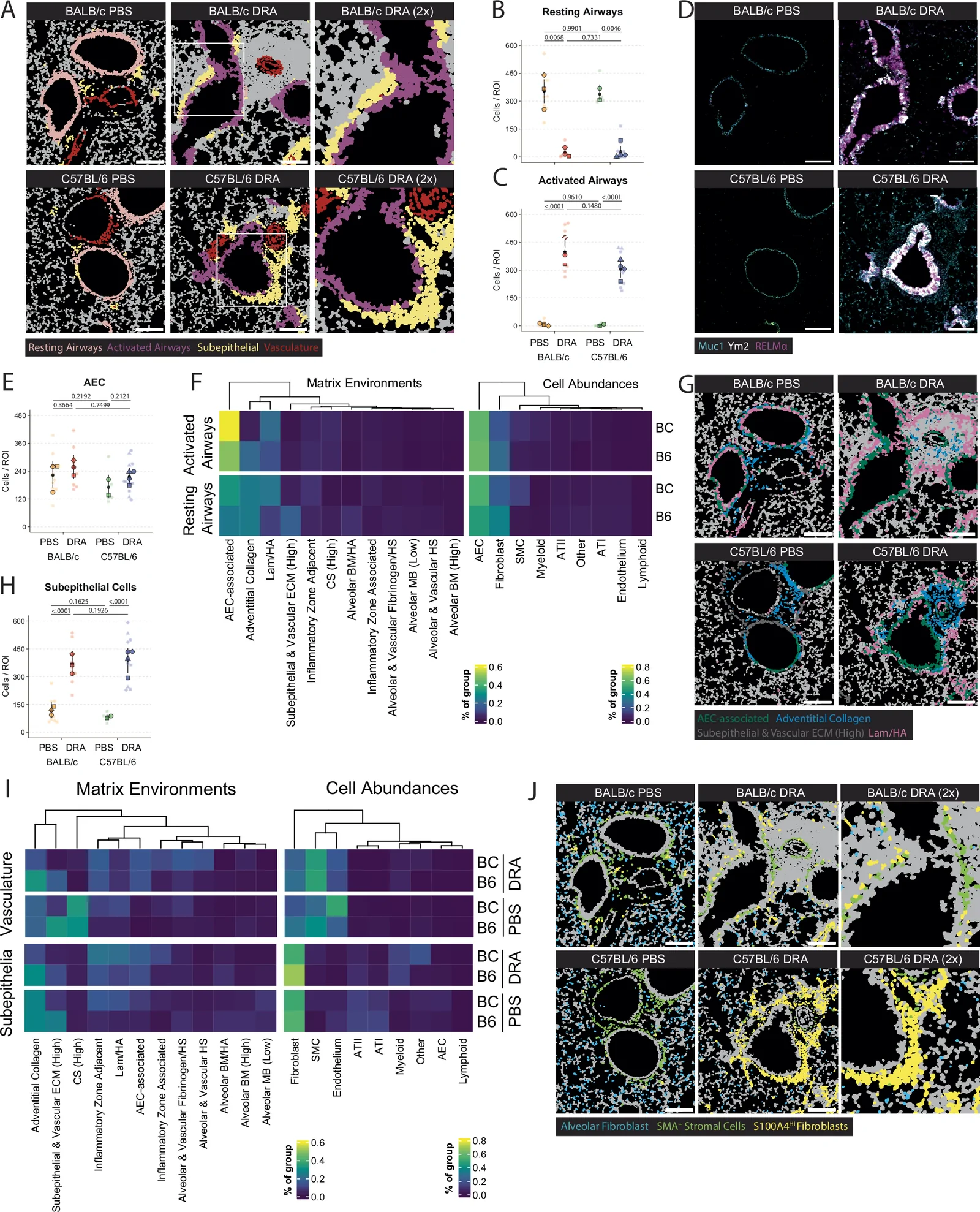
The subepithelial region is a site of immune-stromal interaction after DRA challenge. (**A**) Projection of bronchial epithelium-associated and vascular lisaClust regions onto DeepCell masks. Quantification of the number of cells per ROI in the (**B**) resting airways and (**C**) activated airways lisaClust regions. (**D**) Representative images showing the expression of specific activation markers. (**E**) Quantification of (**D**) the total number of airway epithelial cells (AEC). (**F**) To visualise the enrichment of specific matrix environments in the resting and activated airways lisaClust regions contingency tables were generated and plotted via heatmap. The left panel shows the percentage of cells in that region associated with the specific matrix environment. The right panel shows the percentage of cells in the region from previously defined cell classes. (**G**) Representative images showing the localisation of airway and airway-associated lisaClust regions in representative ROIs. Quantification of the mean cells per ROI in the (**H**) subepithelial lisaClust region. (**I**) Contingency tables visualising the enrichment of specific matrix environments in the subepithelial and vasculature lisaClust regions plotted via heatmap. The left panel shows the percentage of cells in that region associated with the specific matrix environment. The right panel shows the percentage of cells in the region from previously defined cell classes. (**J**) Identified fibroblast populations projected onto DeepCell masks of representative images. For (**A**, **D**, **G**, **J**) the same representative sample from each group are shown; scale bars 130 μm. For (**F**, **I**) both scales represent the frequency of the region comprised of that matrix environment or cell type in the specified region across strains. For panels (**B**,** C**, **E**, **H**), smaller background data points represent individual ROIs, larger points show the average per animals, with black error bars showing the 95% confidence intervals around the mean (black filled circle). *n* = 2–4 animals with three corresponding ROIs per sample; mean and error bars are calculated from all ROIs. Statistical significance was calculated with linear mixed-effects models using PROC MIXED in SAS, with *P* values shown on the graph; see Methods for full details. Source data are available online for this figure.

**Figure EV6:**
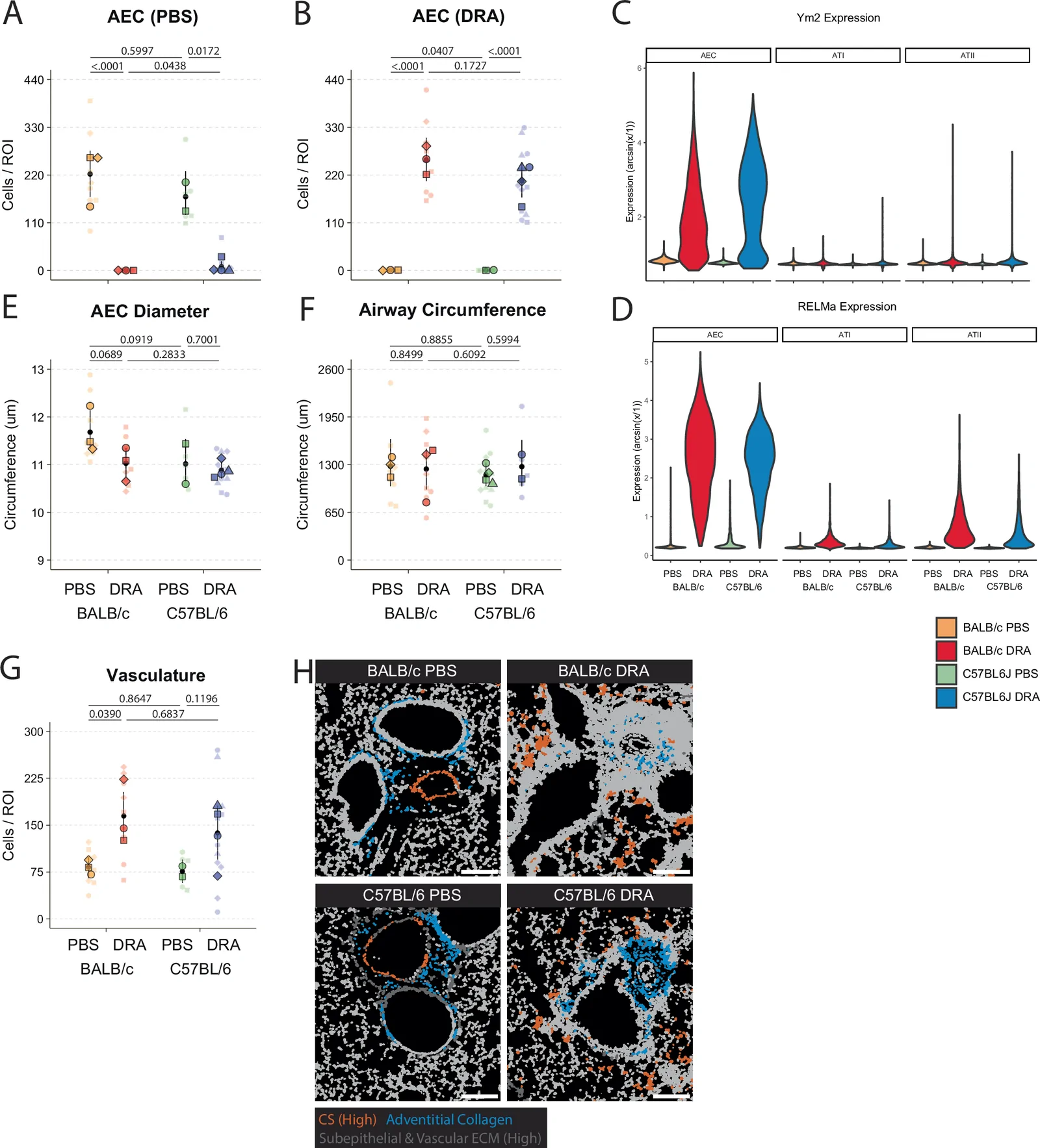
Airway-associated changes in cells and matrix during AAI. Quantification of the mean cells per ROI of (**A**) resting AEC (PBS) and (**B**) activated AEC (DRA) per ROI. Violin plots showing the mean expression per cell of canonical epithelial activation markers (**C**) Ym2, and (**D**) RELMα. Quantification of the mean diameter of AECs (**E**) and total airway circumference (**F**) per ROI. (**G**) Quantification of the number of cells in the vasculature lisaClust region per ROI. (**H**) Representative ROIs showing cells in select matrix environments. For panels (**A**,** B**,** E**–**G**) smaller background data points represent individual ROIs, larger points show the average per animals, with black error bars showing the 95% confidence intervals around the mean (black filled circle). *n* = 2–4 animals with three corresponding ROIs per sample. For panels (**C**, **D**), violin plots represent the mean expression calculated across the cell mask. *n* = 2–4 animals per group corresponding to 6–12 ROIs per group; mean and error bars are calculated from all ROIs. Statistical significance was calculated with linear mixed-effects models using PROC MIXED in SAS, with *P* values shown on the graph; see Methods for full details.

Activation of AEC and subepithelial matrix deposition are key features of airway remodelling. To identify cells contributing to ECM changes around the airways during AAI, we focussed on four spatially adjacent lisaClust regions: resting and activated airways, subepithelial cells (localising basally beneath the airway epithelium), and vasculature (blood vessels running parallel to the airways) (Fig. 6A). Following DRA administration, the prevalence of the resting airway region decreased (Figs. 6B and EV6A) whilst the activated airway region expanded (Figs. 6C and EV6B). This shift corresponded with increased Ym2 and RELMα expression within the AECs (Figs. 6D and EV6C,D), molecules associated with epithelial activation (Chenery et al, 2021; Parkinson et al, 2021). The total number of AECs (Fig. 6E), AEC diameter (Fig. EV6E) and total airway circumference (Fig. EV6F) were unchanged between the strains before or after DRA challenge.

To determine whether airway activation was associated with changes in the surrounding ECM composition or organisation, we examined airway-associated matrix environments alongside their cellular composition. Activated airways were more closely linked with the AEC-associated and Lam/HA matrix environments (Fig. 6F,G), characterised by proximity to HA, laminin γ1, and fibronectin (Fig. 2D). Notably, the adventitial collagen matrix environment, enriched for type-I, III, and VI collagens, was less prevalent in activated BALB/c airways but maintained in C57BL/6 counterparts (Fig. 6F,G), suggesting strain-specific differences in airway collagen organisation during allergic inflammation.

Despite this reduction in collagen-rich matrix environments around airways in BALB/c mice, total subepithelial collagen was unchanged as previously observed (Parkinson et al, 2021), prompting closer examination of the adjacent subepithelial region identified by lisaClust. Cell numbers within this region increased significantly following DRA-treatment in both strains (Fig. 6H), while expansion of the vascular region was observed only in BALB/c mice (Fig. EV6H). The vascular region comprised smooth muscle cells, endothelial cells and fewer fibroblasts (Fig. 6I). In PBS-treated animals, this region was characterised by a CS-high matrix environment, which was largely lost following DRA treatment (Figs. 6I and EV6I). This loss of vascular CS correlated with an increase in alveolar CS (Figs. 5O,Q and EV6I), pointing to spatially divergent remodelling of CS across lung compartments during allergic inflammation.

The subepithelial matrix environment was broadly similar across PBS and DRA-treated animals. There was a general enrichment for adventitial collagen and subepithelial and vascular ECM regions within the subepithelial cells (Fig. 6I), consistent with proximity to both type I and III collagens (Fig. 2D), two of the major isoforms deposited under the airways in this model (Parkinson et al, 2021). The subepithelial region was primarily composed of fibroblasts (Fig. 6I), and this could be split into two main populations, SMA^+^ stromal cells and S100a4^+^ fibroblasts (Figs. 6J and 2D). Together, spatial analysis of the airway epithelial regions of the lungs suggests reorganisation of the stromal compartment within the subepithelial space, a region of the lung that undergoes vast remodelling during AAI.

### CD11b^+^ immune cells localise close to S100a4^+^ subepithelial fibroblasts during AAI

**Figure 7:**
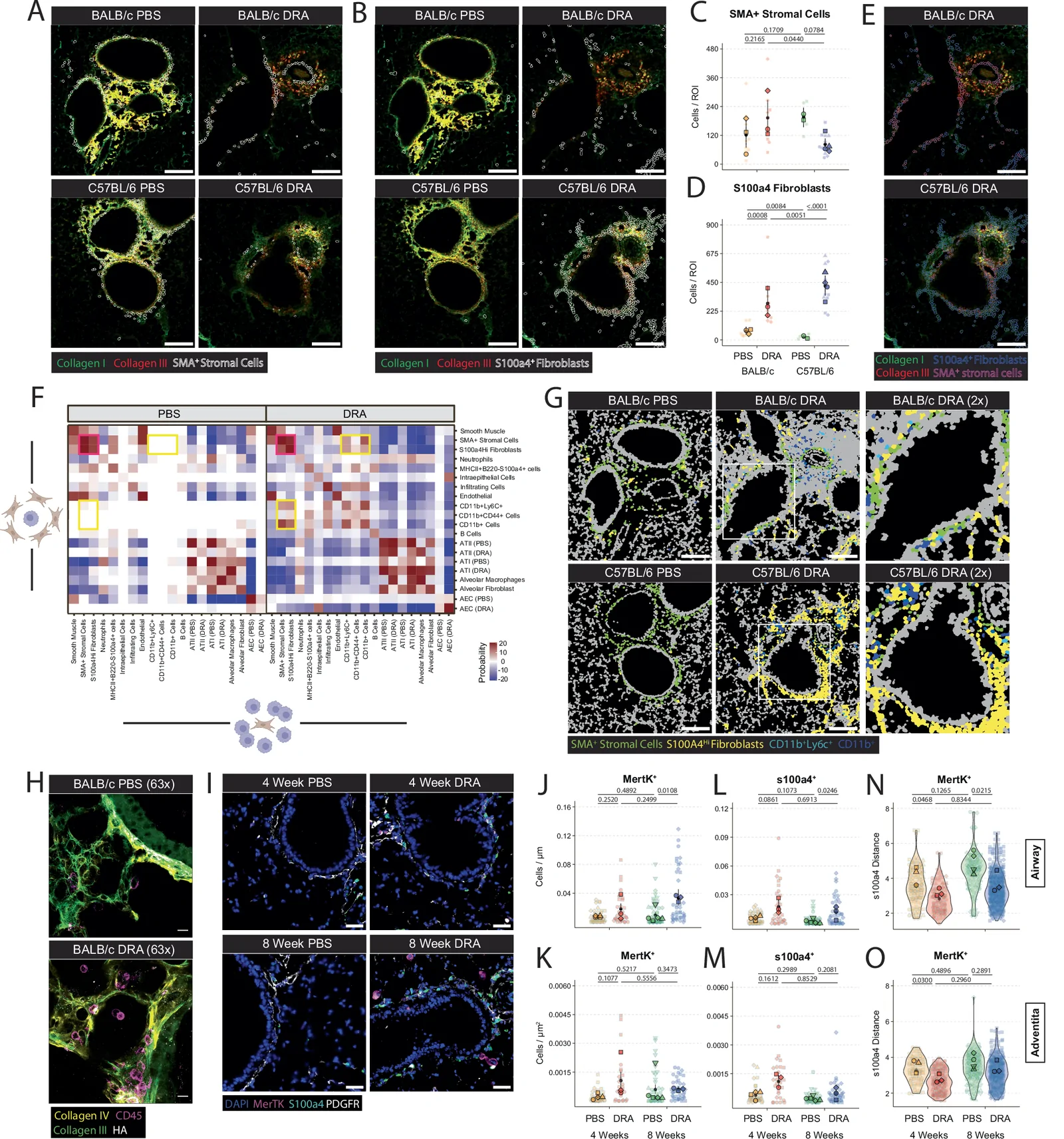
S100a4^+^ subepithelial fibroblasts localise with CD11b^+^ immune cells during AAI. Representative images showing the localisation of (**A**) SMA^+^ stromal cells and (**B**) S100a4^+^ fibroblasts in relation to type-I and type-III collagen. Quantification of the mean number per ROI of (**C**) SMA^+^ stromal cells and (**D**) S100a4^+^ fibroblasts per ROI. (**E**) Representative ROIs (DRA) showing type-I and type-III collagen expression and the localisation of both SMA^+^ stromal cells and S100a4^+^ fibroblasts. (**F**) Heatmap showing the probability that distinct cell populations are found in close proximity more than random chance in PBS and DRA animals, as calculated using neighbourhood analysis (Schapiro et al, 2017). Magenta box highlighting the interaction of SMA^+^ stromal cells and S100a4^+^ fibroblasts in PBS and DRA-treated animals. The yellow box shows the DRA-treatment-specific interaction of S100a4^+^ fibroblasts with CD11b^+^ populations. (**G**) Representative images showing the projection of key stromal and CD11b^+^ cell populations on DeepCell-generated masks. (**H**) PCLS immunofluorescent staining of 2-week DRA-challenged BALB/c mice showing the presence of CD45^+^ cells near the subepithelium, away from the adventitial cuff. To further characterise the influx of CD11b^+^ cells, IF staining was performed on both 4-week and 8-week PBS and DRA-treated C57BL/6 animals. (**I**) Representative images showing immunostaining of MertK (magenta), S100a4 (green), PDGFRα (white), and DAPI (blue). Quantification of MertK^+^ cells around the (**J**) airway and (**K**) adventitia. Quantification of S100a4^+^ cells around the (**L**) airway and (**M**) adventitia. To determine if MertK^+^ cells were interacting with S100a4^+^ cells, the distances of each marker from the respective positive cell was calculated. Distance to closest S100a4^+^ staining from MertK^+^ classified cells within the (**N**) airway and (**O**) adventitia. For (**A**, **B**, **E**, **G**) the same representative sample from each group are shown; scale bars 13 μm. For (**H**) all imaged animals are shown; scale bars 10 μm. For (**i**) images are representative of 3–5 animals; scale bars 70 μm. For (**C**,** D**, **J**–**O**), smaller background data points represent individual ROIs, larger points show the average per animals, with black error bars showing the 95% confidence intervals around the mean (black filled circle); mean and error bars are calculated from all ROIs. For (**C**, **D**), *n* = 2–4 animals with three corresponding ROIs per sample and for (**J**–**O**), *n* = 3–4 with at least six corresponding ROIs per sample; mean and error bars are calculated from all ROIs. Statistical significance was calculated with linear mixed-effects models using PROC MIXED in SAS, with *P* values shown on the graph; see Methods for full details. Source data are available online for this figure.

Both SMA^+^ stromal cells (Fig. 7A) and S100a4^+^ fibroblasts (Fig. 7B) localised closely to collagen-I and III within the subepithelial region during DRA-induced ECM remodelling. SMA^+^ stromal cells were present in substantial numbers in PBS-treated animals, and this was maintained following DRA administration (Fig. 7C). In contrast, S100a4^+^ fibroblasts were relatively sparse in PBS-treated mice but expanded significantly during allergic challenge in both strains (Fig. 7D), consistent with active ECM remodelling in these animals.

**Figure EV7:**
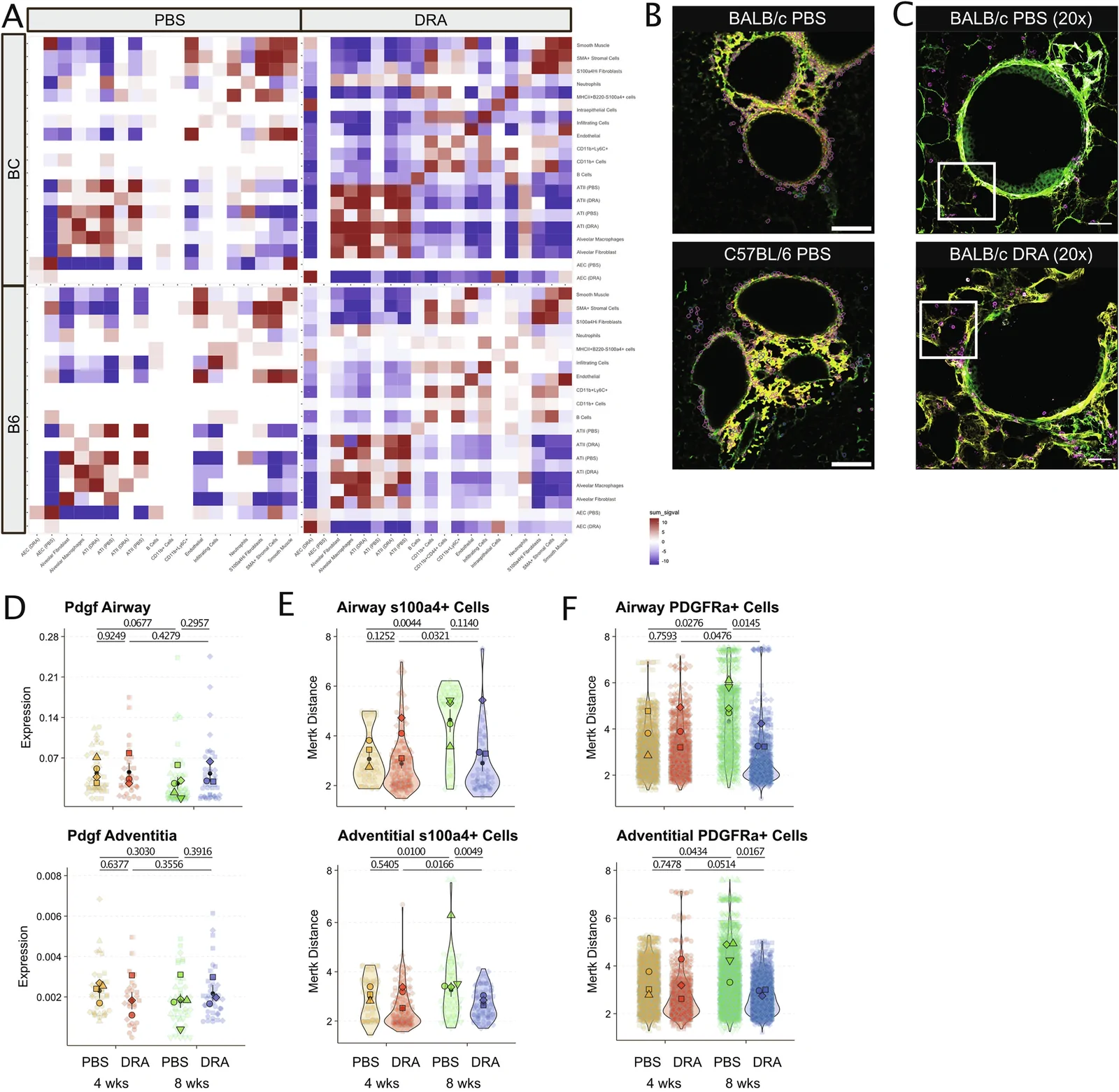
Macrophages and fibroblasts co-localise in the subepithelial niche during AAI. (**A**) Further characterisation of the neighbourhood analysis with data split between strains and treatments. (**B**) Representative ROIs (PBS-treated) showing type-I and type-III collagen expression and the localisation of both SMA^+^ stromal cells and S100a4^+^ fibroblasts. Scale bar: 130 µm. (**C**) PCLS immunofluorescent staining of 2-week PBS-challenged BALB/c mice. Scale bar: 50 µm (**D**) Quantification of the number of PDGFRα^+^ cells in the airway and adventitial space. (**E**) Quantification of the distance of S100a4^+^ cells from the closest MertK^+^ staining in the airway and adventitia. (**F**) Quantification of the distance of PDGFRα^+^ cells from the closest MertK^+^ staining in the airway and adventitia. For (**D**–**F**) graphs are violin plots with overlaid boxplots. The thick central line represents the median, with the box showing the interquartile range and lines representing Q1 and Q4; outliers are shown as individual points. Statistical significance was calculated using PROC MIXED or PROC GLM (for **D**–**F**) in SAS, with *P* values shown on the graph; see Methods for full details. For panels (**D**–**F**) data points represent individual ROIs with error bars showing the 95% confidence intervals around the mean (black filled circle). For panels (**E**,** F**), violin plots represent the mean expression calculated across the cell mask. *n* = 2–4 animals per group corresponding to 6–12 ROIs per group; mean and error bars are calculated from all ROIs. Statistical significance was calculated with linear mixed-effects models using PROC MIXED in SAS; see Methods for full details.

Given their shared anatomical localisation within the subepithelial region (Fig. 7E), neighbourhood analysis was used to determine whether these two stromal populations were interacting during AAI-induced airway remodelling. SMA^+^ stromal cells and S100a4^+^ fibroblasts were found in close proximity in both the steady state and during AAI (Figs. 7F—magenta box and EV7A), confirming the co-localisation we observed in the subepithelial region (Figs. 6J and 7E). Neighbourhood analysis further revealed that both these stromal cell populations interacted with CD11b^+^ and CD11b^+^Ly6C^+^ populations during AAI in both strains (Fig. 7F—yellow box and 7G). Immunofluorescence of PCLS from PBS and DRA-treated BALB/c animals after 4-weeks of allergen-treatment confirmed the presence of CD45^+^ cells in close proximity to subepithelial type-III collagen around the airways (Figs. 7H and EV7B). This observation was consistent with immune cell recruitment to this remodelling niche.

To better characterise the influx of CD11b^+^ cells into subepithelial region, higher resolution IF staining was performed on lung sections from C57BL/6 animals administered PBS or DRA for 4 or 8-weeks. C57BL/6 mice were selected for subsequent analysis to minimise the confounding influence of the pronounced adventitial cuff inflammation in BALB/c mice, allowing analysis to focus on precise macrophage and fibroblast interactions within the subepithelial compartment specifically. Staining for MerTK to identify macrophages, alongside S100a4 and PDGFRα to classify fibroblast populations, identified these key cell populations within the adventitia and around the airways (Fig. 7I). The adventitia and airways were manually segmented, and cell numbers normalised to the size of each region. MerTK^+^ cells significantly increased around the airways (Fig. 7J), but not the adventitia (Fig. 7K) at 8-weeks post DRA challenge. Similarly, S100a4^+^ cells were expanded around the airways (Fig. 7L) but not within the adventitia (Fig. 7M) at the same timepoint. PDGFRα^+^ cells were not increased in the airways or adventitia at either timepoint (Fig. EV7D).

To quantify the spatial relationship between MerTK^+^ and S100a4^+^ cells, the distance from each MerTK^+^ cell to the nearest S100a4+ cell was calculated, and vice versa. MertK^+^ cells consistently showed a consistent reduction in distance to S100a4^+^ staining around the airway after DRA-treatment (Fig. 7N). A similar reduction was observed in the adventitia but only at 4-weeks post challenge (Fig. 7O). S100a4^+^ cells showed no change in the distance to MertK^+^ staining around the airways after allergen administration but did exhibit a reduced distance at 8-weeks within the adventitia (Fig. EV7E), suggesting that only a subset of S100a4+ cells interact with the MerTK^+^ population. A similar pattern was observed for PDGFRα^+^ cells, which showed reduced distance to MerTK^+^ cells across both regions at 8-weeks (Fig. EV7F). Notably, both S100a4^+^ and PDGFRα^+^ cells showed increased distance to MerTK^+^ cells in PBS-treated animals between week 4 and 8 suggesting possible age-related or PBS related changes in the spatial organisation of this region independent of allergen exposure.

Together, these data indicate that a subset of macrophages accumulate within the subepithelial niche during allergen challenge, where they interact with SMA^+^ stromal cells and an expanding S100a4^+^ fibroblast population. Given that ECM remodelling is concentrated in this region, these cellular interactions may constitute an important regulatory network for controlling ECM deposition during allergic pathology.

## Discussion

We have characterised changes across three distinct lung compartments, the alveolar parenchyma, adventitial cuff, and airway epithelium during AAI. Integration of spatial cellular analysis with direct ECM characterisation allows mapping of these changes across the lung following allergic pathology. Studies in murine models of AAI have traditionally focused on small and medium airways as the primary sites of remodelling. However, our data reveal broader alterations across the lung including remodelling of the alveolar parenchyma, strain-specific differences in adventitial immune cell recruitment, and reorganisation of the subepithelial stromal compartment. By employing a cross-sectional design on a defined chronic disease state, with validation across additional timepoints, we have been able to place cellular changes within the context of the dynamic ECM environment. Albeit, future work will be needed to clarify the temporal sequence of events during AAI and establish causal relationships.

Distinct ECM environments were identified in the alveolar parenchyma of DRA-challenged mice. This aligns with human studies showing that matrix turnover in the alveolar compartment differs between controlled and uncontrolled asthma (Weitoft et al, 2014). Our matrix analysis specifically identified changes in hyaluronan and chondroitin sulphate, ECM components known to regulate cellular infiltration into the tissue (Pally and Naba, 2024; Yamada and Sixt, 2019; Yamada et al, 2019). These molecules may, therefore, play an important role in controlling the immune tissue niche and more specifically localisation of alveolar macrophages and neutrophils. In support of this, chondroitinase treatment during bleomycin-induced pulmonary fibrosis in mice reduced the number of alveolar macrophages (Kai et al, 2007), suggesting chondroitin sulphate might directly influence macrophage retention and spatial dynamics within the lung. Tissue-resident alveolar macrophages are generally considered tissue protective (Zasłona et al, 2014), in part through preventing neutrophil-mediated tissue damage (Uderhardt et al, 2019). During allergen sensitisation, these resident cells are replaced (Feo-Lucas et al, 2023), mainly through recruitment and differentiation of circulating monocytes (T’Jonck and Bain, 2023) a process associated with alterations in alveolar morphology and epithelial surfactant dysfunction (Feo-Lucas et al, 2023). Consistent with this, we identified shifts in the ratio of alveolar epithelial cells which are key regulators downstream immune responses (Feo-Lucas et al, 2023; Ma et al, 2024; Katzen and Beers, 2020). These structural changes may then have broader consequences for the progression of allergic pathology.

We have recently shown that Ym1, an ECM-binding molecule produced by alveolar macrophages, is required for subepithelial collagen deposition in this model. However, Ym1-deficient animals show increased alveolar elastance (Parkinson et al, 2025), indicating greater lung stiffness and suggesting that Ym1 may play a protective role in maintaining alveolar mechanical integrity. These findings point toward a potential communication axis between alveolar epithelial cells and Ym1^+^ alveolar macrophages that may be particularly active when there is damage to the alveolar environment, for example, during DRA-induced alveolar remodelling. Consistent with broader remodelling of the alveolar ECM, others have described a switch from heparan sulphate to chondroitin sulphate dominated matrices in the lung during fibrotic lung disease (Karlinsky, 1982). This is likely driven by alterations in fibroblasts and epithelial cells. Alveolar fibroblasts are known to adopt distinct phenotypic states during inflammatory and fibrotic responses (Tsukui et al, 2024). Better characterisation of these populations in naive and inflammatory settings will be vital for understanding how the alveolar niche responds to perturbations in the environment. Importantly, our findings reveal substantial remodelling of alveolar cell populations and ECM composition during allergic airway inflammation, laying the groundwork for future investigations into the functional consequences of these changes.

We identified distinct differences in adventitial immune cell recruitment between BALB/c and C57BL/6 mice, which is notable given our previous work showing comparable proportions of inflammatory cells by flow cytometry (Parkinson et al, 2021). This discrepancy likely reflects differences in the localisation of certain populations rather than their overall abundance and underscores the value of spatial analysis beyond its application to ECM characterisation. Resolution of individual cell populations within the adventitial cuff was limited in our IMC study, but subsequent IF analysis demonstrated eosinophils, interstitial macrophages, and monocytes were all present. Notably, iBALT-like tissue structures were only clearly identified in BALB/c animals, with a spatially organised cellular architecture suggesting functional zonation of different immune and stromal cell populations such within the cuff. The formation iBALT-like structures in BALB/c but not C57BL/6 mice may reflect strain-specific immune responses to DRA exposure. During chronic inflammation, B cell development and antigen presentation pathways are differentially regulated in the lungs at the transcript level between these strains at 8 weeks (Parkinson et al, 2021). The earlier type-2 skewing characteristic of BALB/c mice (Parkinson et al, 2021) may further drive B cell proliferation and provide the supportive signals required for initial seeding of lymphoid tissue. Fibroblast-immune cell networks are also likely to be important within this niche. Eosinophils and fibroblasts have been shown to cross-regulate one another through several soluble mediators (Zeng et al, 2022), and eosinophils isolated from people with asthma can induce a distinct fibroblast phenotype associated with ECM remodelling (Janulaityte et al, 2022). Therefore, it is possible that adventitial immune composition may directly influence local matrix deposition.

The airways are a well-established site of pathological remodelling in asthma. The subepithelial region adjacent to the airway smooth muscle layer expanded during DRA challenge and was enriched for fibroblasts and MerTK^+^ CD11b^+^ immune cells, likely representing interstitial macrophages. Interstitial macrophages are a heterogenous cell population whose functional diversity is only recently being explored (Moore et al, 2023; Ural et al, 2020; Bain and MacDonald, 2022). These cells accumulate in the lungs of mice during both fibrosis (Misharin et al, 2013) and DRA-induced AAI (Parkinson et al, 2021), yet their influence on disease progression remains context dependent. CpG-induced expansion of IL-10-producing interstitial macrophages was protective during HDM-induced AAI (Sabatel et al, 2017), whereas depletion of interstitial macrophages in radiation-induced lung fibrosis has protective effects (Meziani et al, 2018). This suggests that their roles may be determined by functional subtype rather than population size, and our data do not yet resolve which subset is driving the interactions we observe.

Macrophages are known to both receive and provide cues to fibroblasts (Buechler et al, 2021), consistent with our observation of CD11b^+^ and MerTK^+^ cells in close proximity to expanded S100a4^+^ fibroblasts and SMA^+^ stromal cells within the subepithelial region. These interactions may be particularly significant given that S100a4 expression in fibroblasts increases during inflammation and fibrosis (Tsukui et al, 2024). Recent work has identified a fibroblast population characterised by contractile genes interacting with macrophages via a Cxcl12/Cxcr4 pathway (Yu et al, 2024). Notably, the subepithelial ECM environment in our dataset was enriched for type-VI collagen, amongst other ECM molecules. During pulmonary fibrosis, macrophages within the interstitial space can influence fibroblast function through type-VI collagen secretion (Ucero et al, 2019) which in turn prevents fibroblast apoptosis (Rühl et al, 1999) and promotes fibroblast migration (Sardone et al, 2016). Together, our spatial studies support a model of bidirectional communication between fibroblasts and macrophages within the subepithelial niche, mediating at least in part remodelling of the surrounding ECM.

Despite rapid advances in the characterisation of pulmonary immune and stromal populations by single-cell RNA sequencing (Vieira Braga et al, 2019; Tang et al, 2022), translation of transcriptome-defined markers into validated antibody panels for imaging approaches remains challenging. Assessment of detailed cellular populations more broadly requires reliable, optimised, and validated markers suitable for multiplexed imaging approaches. A parallel challenge exists for ECM characterisation, with cellular transcriptome profiles of matrix-related genes often poor surrogates for bioactive post-translationally modified ECM molecules present in the tissue. Therefore, spatial and molecular analysis of ECM networks remains essential for understanding its true composition and organisation. Whilst computational approaches for integrating ECM pathway analysis with single-cell transcriptomic data are emerging (Kontio et al, 2022), molecular and spatial analysis will be critical to confirm and contextualise these findings.

ECM changes are increasingly recognised as drivers of pathology in asthma (Burgess et al, 2024). However, the relationship between airway inflammation and airway remodelling remains unclear, although studies have suggested these processes may be at least partially independent (O’Reilly et al, 2013; Saglani et al, 2007), though this remains difficult to validate in systematic review (Castro-Rodriguez et al, 2018), given the inherent heterogeneity of patient endotypes (Kaur and Chupp, 2019). Asthma encompasses a broad spectrum of disease presentations with distinct pathological and aetiological profiles. Whilst chronic allergic models using in-bred mice are a powerful tool for studying defined features of airway remodelling, an individual model cannot capture this complexity. Detailed spatial quantification of matrix composition, particularly when combined with cellular and clinical patient metadata, may offer a route to better stratifying patients and understanding how ECM changes contribute to functional decline in different endotypes.

To this extent, we developed this pipeline using formalin-fixed paraffin-embedded tissue specifically to facilitate future application to human biobanked samples, where this is the predominant preservation methodology. Integration of cellular signals with spatial ECM analysis in future studies will be an important step, given the key role the ECM plays in shaping cellular responses (Sutherland et al, 2023; Sorokin, 2010). By integrating cellular and matrix characterisation within a single histological section, this approach captures a richer understanding of the tissue microenvironment than either analysis in isolation. Ultimately, understanding how cell-cell and cell-ECM networks are dysregulated in disease will require moving beyond descriptive cataloguing toward spatially resolved, mechanistically informed analysis. The framework presented here provides a foundation for doing exactly that in both experimental and clinical settings.

## Methods

**Table:** Reagents and tools table

Reagent/resource	Reference or source	Identifier or catalogue number
**Experimental models**		
BALB/c^olaHsd^ (*M. musculus*)	Envigo (Inotiv)	Cat # 162
C57BL/6^olaHsd^ (*M. musculus*)	Envigo (Inotiv)	Cat # 057
**Antibodies**		
Ym2 (IMC)	PMID 33587776	n/a
αSMA (IMC)	Standard Biotools	3141017D
Collagen VI (α1) (IMC)	Abcam	Ab229450
Heparan Sulphate (IMC)	Amsbio	270255-1
Desmin (IMC)	Novus	NBP2-54503
S100a4 (IMC, IF, PCLS)	BioLegend	810101
SPC (IMC)	Abcam	Ab222929
B220 (IMC)	eBioscience	12-0452-82
Pan-cytokeratin (IMC)	BioLegend	628602
CD11b (IMC)	Abcam	Ab216445
RELMa (IMC)	Peprotech	500-P214
Ym1 (IMC)	Novus	AF2446
Collagen IV (α1) (IMC)	Abcam	Ab2256353
Chondroitin sulphate (IMC)	Abcam	Ab11570
Scgb1a1 (IMC)	Novus	NBP1-57961
Vimentin (IMC)	Antibodies.com	A86652-100
E-cadherin (IMC)	Standard Biotools	3158029D
Fibrinogen (IMC)	Abcam	Ab227063
CD44 (IMC)	BioLegend	103002
Ly6C (IMC)	Thermo Fisher	MA1-81899
MHCII (IMC)	Novus	NBP1-43312
vWF (IMC)	Merck	AB7356
Collagen III (α1) (IMC, IF, PCLS)	Southern Biotech	1330-01
Fibronectin (IMC)	Abcam	Ab268022
Laminin γ1 (IMC)	Abcam	Ab234963
Collagen I (IMC)	Standard Biotools	3169023D
Podoplanin (IMC)	BioLegend	127416
Muc1 (IMC)	Thermo Fisher	PA595487
MerTK (IMC)	Abcam	Ab250715
PDGFRα (IMC)	Abcam	Ab234965
Ly6G (IMC)	BioLegend	127602
Biotin (IMC)	BioLegend	409002
PE (IMC)	BioLegend	408102
FITC (IMC)	BioLegend	408302
Rage (IF)	Bio-Techne	AF1145
SPC (IF)	Abcam	ab211326
MertK (IF)	Bio-Techne	BAF591
Collagen IV (α1) (IF)	Abcam	ab236640
Pdgfr α (IF)	Bio-Techne	AF1062
Anti-Rabbit (IF)	Bio-Techne	NL005
Anti-goat (IF)	Bio-Techne	NL002
Anti-rabbit (IF)	Bio-Techne	NL006
Anti-rabbit (IF)	Bio-Techne	NL004
Anti-goat (IF)	Bio-Techne	NL001
CD64 (PCLS)	BioLegend	139321
SigF (PCLS)	Thermo Fisher	406-1702-82
Cd11b (PCLS)	BioLegend	101204
Collagen I (PCLS)	Southern Biotech	1310-01
Cd45 (PCLS)	BioLegend	103123
Cd11b (PCLS)	BioLegend	101219
Collagen IV (α1) (PCLS)	Bio-Techne	NB120-6586
Cd11c (PCLS)	BioLegend	117334
Anti-Goat (PCLS)	ThermoFisher	A48259
Anti-Goat (PCLS)	Thermo Fisher	SA5-10088
Anti-Rabbit (PCLS)	Thermo Fisher	SA5-10040
**Chemicals, enzymes and other reagents**		
*Dermatophagoides pteronyssinus*	Greer Laboratories	XPB91D3A2.5
*Ambrosia artemisiifolia*	Greer Laboratories	XP56D3A25
*Aspergillus fumigatus*	Greer Laboratories	XPM3D3A25
Hyaluronan binding protein-biotin	Sigma	385911
DNA-intercalator	Standard Biotools	201192B
DAPI	Sigma	D9542
Syto9 Green	Thermo Fisher	S34854
Streptavidin NL557	Bio-Techne	NL999
Streptavidin NL637	Bio-Techne	NL998
Streptavidin AF532	Thermo Fisher	S11224
Low-gelling temperature agarose	Sigma	A9045
HBSS without Ca and Mg	Gibco	14170-112
DMEM high glucose	Sigma	D5796
Insulin-transferrin-selenium	Gibco	41400-045
Amphotericin B	Sigma	A2942
MaxPar antibody conjugation kit	Standard Biotools	201145B
10% neutral buffered formalin	VWR	11699455
Ethylenediaminetetraacetic acid	Fisher Scientific	15748687
PBS (tablets)	Merck	P4417
Ready Probes Avidin/biotin blocking kit	Thermo Fisher	R37627
BSA	Sigma	A7906
ProLong Gold Antifade Mount	Thermo Fisher	P36930
Fc Block	BD Bioscience	553142
**Software**		
QuPath v0.7.0	https://qupath.github.io	
Zen lite v3.10	https://www.zeiss.com/microscopy/en/products/software/zeiss-zen-lite.html	
Arivis Cloud	https://www.zeiss.com/microscopy/en/products/software/arivis-cloud-ai.html	
SAS 9.4 TS level 1M3 (Windows)	https://www.sas.com/en_gb/software/viya.html	
Histocat++	https://github.com/BodenmillerGroup/histoCAT3D	

### Animals

BALB/c^olaHsd^ and C57BL/6J^olaHsd^ mice were obtained from a commercial supplier (Envigo, Hillcrest, UK). All IMC experimental animals were female and between 8 and 11 weeks of age at the start of the experiment. Mice were housed in individually ventilated cages in groups of up to five animals in specific pathogen-free facilities at the Biological Services Facility, the University of Manchester or the University of Aberdeen. Mice were randomly assigned to a treatment group. Investigators were not blinded to mouse identity during experiments. Sample size was calculated based on pilot experiments (*N* = 3 per group), based on the number of animals needed for detection of a 25% change in Masson’s trichrome-positive area around the airway in PBS *versus* allergic mice (Power = 0.8, *P* value <0.05). All animal experiments were performed in accordance with the UK Animals (Scientific Procedures) Act of 1986 under Project Licences (70/8548 or PP0624245) granted by the UK Home Office, approved by the University of Manchester or University of Aberdeen Animal Welfare and Ethical Review Body. Animals were euthanised by asphyxiation in rising concentrations of carbon dioxide, or for PCLS animals were euthanised by anaesthetic overdose, and confirmation by cessation of circulation.

### Allergic airway inflammation model

A model of allergic airway inflammation was induced in animals as previously described (Goplen et al, 2009; Parkinson et al, 2021). Allergen cocktail containing 5 µg house dust mite (*Dermatophagoides pteronyssinus*, 5450 EU, 69.23 mg per vial), 50 µg ragweed (*Ambrosia artemisiifolia*) and 5 µg *Aspergillus fumigatus* extracts (Greer Laboratories, Lenoir, NC, USA) was freshly prepared prior to installation. Mice were administered either 20 µL of DRA cocktail or PBS via intranasal instillation. This occurred twice weekly for up to 8 weeks following anesthetisation by isoflurane inhalation. Mice were then rested for 5 days prior to collecting lung tissue.

### Lung preparation for immunostaining

After euthanasia, bronchoalveolar lavage was taken as described previously (Parkinson et al, 2021), and the right main bronchi was ligated with thread and the associated lobes removed for other analysis. The left lobe was then inflated with 0.5 mL of 10% neutral buffered formalin (NBF) before being resected and fixed overnight in 10 mL of NBF. Following this, the tissues were transferred to 70% ethanol for at least 24 h or until processed. Tissues were processed and embedded in paraffin wax using standard protocols. Tissue sections (5 µm) were prepared using a microtome onto glass slides (Superfrost Plus adhesion). Tissue sections were cut immediately prior to staining with antibodies for imaging mass cytometry (IMC).

### Generation of murine precision-cut lung slices

Following exsanguination, the mouse lungs were exposed and the trachea canulated. The lungs were inflated with 1.4 ml of 2% low-gelling temperature agarose (A9045, Sigma-Aldrich), followed immediately by 0.2 ml of air and cooled with ice until agarose solidified. Whole lungs were extracted and placed into cold HBSS without calcium and magnesium (14170-112, Gibco). Lungs were subsequently dissected into individual lobes before embedding in top-removed syringes with 4% low-gelling point agarose. Lung-agarose cores were mounted into a vibratome bath with ice-cold PBS and cut into 200-μm thick slices (VT-1200-S, Leica Biosystems, 3 mm amplitude, 0.4 mm/s speed). Slices were immediately transferred into cold DMEM high glucose (D5796, Sigma-Aldrich) supplemented with 1x Insulin-Transferrin-Selenium (41400-045, Gibco), Penicillin-Streptomycin (P4333, Sigma-Aldrich) and Amphotericin B (A2942, Sigma-Aldrich). Slices are then used for direct immunofluorescence staining or culture.

### IMC antibody conjugation

Purified, carrier-free antibodies (see Table 1) were conjugated to selected metal isotopes using the MaxPar antibody conjugation kit following the manufacturer’s instructions (Standard BioTools). Briefly, Lanthanide metals were loaded onto MaxPar polymer and incubated for 60 min at room temperature (RT), with regular agitation. Antibodies were partially reduced and coupled with metal-loaded polymer before being washed and concentrated to a volume of ~20 µL using a 50 kDa molecular weight cut-off filter column. Final concentrations of conjugated antibody solutions were measured, and antibodies were stored in stabilisation buffer (Candor) at 4 °C.

**Table 1:** Antibodies for IMC tissue staining.

Antigen	Conjugate	Final Conc (µg/mL)	Clone	Company	Cat #
HABP	Biotin	5.00	N/A	Sigma	385911
Ym2	^115^In	0.48	Rab polyclonal	In-House	In-House
αSMA	^141^Pr	0.20	1a4	Standard Biotools	3141017D
Collagen VI (α1)	^142^Nd	6.14	EPR17072	Abcam	Ab229450
Heparan Sulphate	^143^Nd	1.16	F58-10E4	Amsbio	270255-1
Desmin	^144^Nd	1.44	DES/1711	Novus	NBP2-54503
S100a4	^145^Nd	7.53	Rab polyclonal	BioLegend	810101
SPC	^146^Nd	0.96	EPR19839	Abcam	Ab222929
B220	PE	2.00	RA3-6B2	eBioscience	12-0452-82
Pan-cytokeratin	^148^Nd	9.63	C-11	BioLegend	628602
CD11b	^149^Sm	4.00	EPR1344	Abcam	Ab216445
RELMa	^151^Eu	0.13	Rab polyclonal	Peprotech	500-P214
Ym1	^152^Sm	0.17	Goat polyclonal	Novus	AF2446
Collagen IV (α1)	^153^Eu	3.00	EPR22911-127	Abcam	Ab2256353
Chondroitin Sulphate	^154^Sm	2.78	Cs-56	Abcam	Ab11570
Scgb1a1	^155^Gd	1.75	Rab polyclonal	Novus	NBP1-57961
Vimentin	^156^Gd	0.31	V1.10	Antibodies.com	A86652-100
E-cadherin	^158^Gd	2.50	2.4E + 11	Standard Biotools	3158029D
Fibrinogen	^159^Tb	0.89	EPR18145-84	Abcam	Ab227063
CD44	^160^Gd	5.25	IM7	BioLegend	103002
Ly6C	^162^Dy	3.60	ER-MP20	ThermoFisher	MA1-81899
MHCII	^163^Dy	6.16	M5/114.15.2	Novus	NBP1-43312
vWF	^164^Dy	7.06	Rab polyclonal	Merck	AB7356
Collagen III (α1)	^166^Er	4.38	Goat polyclonal	Southern Biotech	1330-01
Fibronectin	^167^Er	1.07	EPR23110-46	Abcam	Ab268022
Laminin γ1	^168^Er	20.67	EPR21199	Abcam	Ab234963
Collagen I	^169^Tm	1.11	Goat polyclonal	Standard Biotools	3169023D
Podoplanin	FITC	11.60	8.1.1	BioLegend	127416
Muc1	^172^Yb	2.13	Rab polyclonal	ThermoFisher	PA595487
MerTK	^174^Yb	7.94	EPR17534-139	Abcam	Ab250715
PDGFRα	^175^Lu	7.92	Rab polyclonal	Abcam	Ab234965
Ly6G	^176^Yb	4.47	1a8	BioLegend	127602
DNA	^191^Di,^193^Di	0.42 (μM)	N/A	Standard Biotools	201192B

Secondary antibodies					
Anti-Biotin	^89^Y	6.00	1D4-C5	BioLegend	409002
Anti-PE	^147^Sm	5.00	PE001	BioLegend	408102
Anti-FITC	^170^Er	5.80	FIT-22	BioLegend	408302

### Immunostaining

#### IMC staining

Tissue sections were dewaxed through xylene and a decreasing ethanol gradient (100, 90 and 75%) using standard protocols. Antigen retrieval was performed on dewaxed sections using 10 mM Tris-HCl, pH 9.0, 1 mM ethylenediaminetetraacetic acid (EDTA) + 0.02% Tween-20 for 20 min by heating in a microwave. Slides were then permeabilised in 0.2% Triton X-100 in Phosphate Buffered Saline, pH 7.4 (PBS) for 20 min at RT prior to staining. Non-specific protein was blocked with 3% bovine serum albumin (BSA) in PBS for 1 h at RT in a hydration chamber, followed by blocking endogenous biotin according to the manufacturer’s instructions (Ready Probes Avidin/biotin blocking kit, Thermo Fisher). For staining, antibody cocktails (see Table 1) were prepared in PBS with 0.5% BSA. All conjugated antibodies were centrifuged (10,000×*g* for 2 min) immediately prior to use to minimise antibody aggregates. Sections were incubated with primary antibody cocktail overnight at 4 °C in a hydration chamber. Slides were then washed in PBS with 0.2% Triton X-100 (8 min), followed by PBS alone (8 min). Secondary antibodies (see Table 1) were diluted in PBS with 0.5% BSA as before and added to tissue sections for 1 h at RT. Slides were then washed as before and incubated with Iridium Intercalator in PBS (1:1200) for 30 min at RT. Stained slides were washed in dH_2_O for 2 min with gentle agitation and then air-dried for at least 20 min at RT and stored at 4 °C until acquisition.

#### Immunofluorescence FFPE

Tissue sections were processed as for IMC, with protocol variations described below. Non-specific protein was blocked using 2% normal donkey serum in 1%BSA/PBS for 1 h at room temperature, followed by blocking endogenous biotin. For staining, primary antibodies (diluted in 1% BSA/PBS, Table 2) were incubated at 4 °C in a hydration chamber. Slides were washed in PBS prior to adding secondary antibodies (Table 2) diluted in 1% BSA/PBS for 1 h at room temperature. Sections were washed in PBS, stained with DAPI (1 µg/mL, Merck) for 10 min prior to washing in PBS. Autofluorescence signal was blocked by incubating sections in TrueView (2B Scientific) for 3 min prior to washing in PBS and mounting with ProLong Gold Antifade Mountant (P36930, Thermo Fisher).

**Table 2:** Antibodies for immunofluorescence FFPE tissue staining.

Antigen	Conjugate	Final Conc (µg/mL)	Clone	Company	Cat #
Rage		1.00	Goat polyclonal	Biotechne	AF1145
SPC		0.48	EPR19839	Abcam	ab211326
MertK	Biotin	2.00	Goat Polyclonal	Biotechne	BAF591
Collagen IV (α1)		6.14	EPR22911-127	Abcam	ab236640
Collagen III (α1)		1.00	Goat Polyclonal	Southern Biotech	1330-01
Pdgfra		2.00	Goat Polyclonal	Biotechne	AF1062
S100a4		2.00	Rabbit polyclonal	BioLegend	810101
Anti-rabbit	NL637	1:200	Donkey polyclonal	Bio-Techne	NL005
Anti-goat	NL637	1:200	Donkey polyclonal	Bio-Techne	NL002
Anti-rabbit	NL493	1:200	Donkey polyclonal	Bio-Techne	NL006
Anti-rabbit	NL557	1:200	Donkey polyclonal	Bio-Techne	NL004
Anti-goat	NL557	1:200	Donkey polyclonal	Bio-Techne	NL001
Streptavidin	NL557	1:800	Donkey polyclonal	Bio-Techne	NL999
Streptavidin	NL637	1:400	Donkey polyclonal	Bio-Techne	NL998

#### Immunofluorescence PCLS

Washes in PBS were performed between steps in the plates. Slides were pre-circled with a pap pen before sections were transferred into PBS droplets. Staining and blocking steps were performed in 100 μl droplets on slides. Non-specific binding blocking was performed with 1:100 mouse FC block (553142, BD Biosciences) in 1% BSA/PBS for 1 h at 4 °C. Endogenous biotin was blocked using ReadyProbes™ Avidin/Biotin Blocking Solution (R37627, Thermo Fisher) as per manufacturer instructions where required. Slices were incubated with primary antibodies in 1% BSA/PBS overnight at 4 °C (Table 3). Where required, slices were incubated with secondary antibodies in 1% BSA/PBS for 1 h at room temperature. Fixation was performed for 12-min in 10% neutral buffered formalin before staining with nucleic acid stain Syto9 (S34854, Thermo Fisher). Sections were mounted on slides with ProLong Gold Antifade Mountant (P36930, Thermo Fisher) and 1.5# coverslips and stored at 4 °C until imaging.

**Table 3:** Antibodies for PCLS staining.

Antigen	Conjugate	Final Conc (µg/mL)	Clone	Company	Cat #
CD64	AF647	5	X54-5/7.1	BioLegend	139321
SigF	BV605	2	1RNM44N	Thermo Fisher	406-1702-82
Cd11b	Biotin	25	M1/70	BioLegend	101204
Collagen I		4	Goat polyclonal	Southern Biotech	1310-01
Cd45	AF647	5	30-F11	BioLegend	103123
Cd11b	AF488	5	M1/70	BioLegend	101219
Collagen III (α1)		4	Goat polyclonal	Southern Biotech	1330-01
Collagen IV (α1)		1	Rabbit polyclonal	Bio-Techne	NB120-6586
HABP	Biotin	5	n/a	Sigma	385911
S100a4		1	S100A4	BioLegend	810101
Cd11c	BV605	2	N418	BioLegend	117334

Secondary antibodies					
Streptavidin	AF532	10	n/a	Thermo Fisher	S11224
Anti-Goat	AF + 405	10	Donkey Polyclonal	Thermo Fisher	A48259
Anti-Goat	Dylight 594	2.5	Donkey Polyclonal	Thermo Fisher	SA5-10088
Anti-Rabbit	Dylight 594	2.5	Donkey Polyclonal	Thermo Fisher	SA5-10040
SYTO 9 Green		1:3000	n/a	Thermo Fisher	S34854

### IMC image acquisition

IMC data were acquired on the Hyperion® Imaging System (Standard BioTools). The instrument was calibrated using a tuning slide to optimise the sensitivity of the detection range with a signal for 175Lu of at least 1500 mean duals. To capture ROIs, specific 660 × 690 μm regions of interest (ROI) were identified with investigators blinded to mouse identity during acquisition. These were required to have a small-medium airway, associated vasculature, and surrounding alveolar parenchyma. Where possible, ROIs were selected across different lobes. Once selected, ROIs were laser-ablated in 1 μm^2^ pixels before being passed through a plasma source and metal-conjugated antibodies were detected with time-of-flight mass spectrometry. Antibody staining was reviewed with histoCAT++ and data extracted from mcd files.

### Deepthresh matrix thresholding

All regions of interest (ROIs) were first enhanced using IMCDenoise (Lu et al, 2023). Six ROIs were manually thresholded for use in a semi-automated thresholding approach we refer to as DeepThresh. Four images were used for training, one for validation and one for testing. DeepThresh was built using PyTorch (Paszke et al, 2019) (version 2.3) and Pytorch Segmentation Models (qubvel-org/segmentation_models.pytorch, 2024). The architecture mimics that of U-Net (Ronneberger et al, 2015) with a ResNet152 encoder (He et al, 2016). Adam (Kingma and Ba, 2017) optimiser was applied with a learning rate of 0.0001. All models were trained for 50 epochs. In relation to training, a separate DeepThresh model was trained for each channel. ROIs were channel-wise min-max normalised. Each image was zero-padded around the edges to enable splitting into overlapping 64 × 64 patches with a stride of 32. In addition, data augmentation was applied by rotating the patches by 90° and 180°. In terms of inference, the images were zero- padded, split into 64 × 64 non-overlapping patches, normalised and the binary mask predicted from the trained channel-specific models. Subsequently, the patches were reconstructed and the zero-padding was removed (https://github.com/moeghaf/DeepThresh).

### Matrix distance calculation

To accurately analyse ECM molecules, we developed a pipeline using binary thresholding of matrix staining, followed by distance calculation between positive ECM staining and previously generated cell masks (Fig. EV2A). Firstly, processed stacked images were exported from the IMCdenoise (Lu et al, 2023) pipeline (Fig. EV2A i). Manual thresholding was applied to a subset of images to generate binary images of positive ECM marker staining (Fig. EV2A ii). The binary images were then used as training data for DeepThresh (Fig. EV2A iii,B). DeepThresh was then applied to the entire dataset, which allowed thresholding of ECM channels whilst accounting for variances in brightness and background between ROIs (Fig. EV2A iv). Once thresholding had been applied, skimage (Walt et al, 2014) was used to calculate distance maps from binary images (Fig. EV2A v) (https://github.com/jparkinson375/MatrixIMC). Distance maps represented the value of each pixel as its distance from the closest positive pixel in the binary thresholded image. Distance maps were appended to z-stack images prior to importing into the Steinbock pipeline, enabling ECM-cell data integration into the standard pipeline (Fig. EV2A vi). Quantification of average values across DeepCell (Greenwald et al, 2022) generated cell masks, as implemented in the steinbock package, then gave the average distance of each cell from specific ECM components (Fig. EV2A vii).

### IMC image preparation and analysis

Following data extraction, cell mask generation, and cell marker quantification, IMC data were imported into R using the imcRtools package. Batch correction was performed using the R implementation of Harmony, providing the slide number as a variable, to remove variance between different slides when imaged at different times. Following batch correction, dimensionality reduction and clustering were performed using principal component analysis (PCA) (Kendall, 1939; Maćkiewicz and Ratajczak, 1993), multidimensional scaling (MDS) (Nowicka et al, 2019), uniform manifold approximation projection (UMAP) (McInnes et al, 2018), and single nearest neighbours (SNN) (Ertöz et al, 2003), respectively.

### Immunofluorescent FFPE image acquisition and data analysis

Images were acquired using a Zeiss AxioScan Z1 Slide scanner, with all images within an experimental dataset acquired at one time using the same settings. Data were analysed using Qupath (version 0.7.0). Collagen, Rage and SPC staining was analysed by creating a pixel threshold to identify positively stained areas within regions of interest (e.g., alveoli, airways, and adventitial cuff). Collagen staining data were normalised to ROI area (alveoli and adventitial cuff) or basement membrane length (airway). Rage and SPC staining were normalised to the number of cells within an ROI by calculating the positive area covered by DAPI staining. For analysis of macrophages and fibroblasts, cell segmentation was performed within ROIs (airways, adventitial cuff) using Instanseg, followed by creating a threshold classifier to identify Mertk+, S100a4+ and Pdgfra+ cells within each ROI, with numbers normalised to area (cuff) or basement membrane length (airway). This data were used to identify macrophages as Mertk+ or MertK+ S100a4+ cells and the inflammatory fibroblast subset as S100a4+ Pdgfra+ cells. Distance calculations were performed using distance to detection analysis in Qupath for each cell.

### Fluorescent PCLS image acquisition

Immunofluorescent images of PCLS were acquired using a Zeiss LSM 880 confocal microscope with 20x/0.8 NA and 63x/1.4 NA (Oil) Plan-apochromat objectives. Images were acquired as z-stacks with an optical interval of 0.952 µm (20x) or 0.430 µm (63x). The total thickness of the captured z-stacks ranged from 5 to 20 µm, depending on the region of interest. For visualisation, images are displayed as maximum intensity projections generated using ZEN lite 3.10.

### Adventitial cuff segmentation

Region-based semantic segmentation of PCLS immunofluorescent images was performed in Arivis Cloud using a previously trained model. The training dataset comprised 57 nuclear staining z-stack images, including PBS and 1 to 4-week DRA samples. Each image comprised 20 optical planes, of which 4–11 planes were manually annotated, depending on inter-plane heterogeneity. Regions were classified into four categories: Adventitial-cuff, Alveolar-space, Airway and background, with background regions including blood vessels and small airway lumina. The model was trained over three iterative cycles, during which incorrectly or poorly predicted regions were manually re-annotated before retraining. For mask generations, the displayed nuclear staining 10 µm maximum intensity projections were automatically segmented in arivis Cloud and exported as binary masks for downstream overlay.

### Statistical analysis

Data were analysed using SAS 9.4 TS level 1M3 (Windows), and when the residuals, assessed visually using plots = studentpanel(marginal conditional) within SAS, failed to meet the assumptions of homoscedasticity or normal distribution, data were log transformed. Unless specified otherwise data were imported using PROC IMPORT and then statistical significance calculated using PROC MIXED using a repeated measures mixed-effects linear model or general linear model analysis as specified. Strain and treatment were included as CLASS variables (For Figs. 7j–m and EV7d, time and treatment were defined as the CLASS variables, but everything else was the same) with mouse as the SUBJECT for the repeated measures. The MODEL was defined as variable = strain treatment strain*treatment to evaluate the simple and interaction effects of treatment across strains. The Kenward–Roger degrees-of-freedom approximation was used to account for uncertainty in the variance estimates of random effects. Post hoc testing was performed using LSMEANS with the SLICE variable used to assess the simple effects of treatment on either strain or the differences between strains at different treatment levels. Statistical significance is shown on graphs as the interaction effect of treatment across strains for BALB/c DRA vs C57BL/6 DRA comparison, or for within-strain/treatments as the significance of this comparison in isolation.

Specifically for the distance calculations in Figs. 7n, o and EV7e,f data were again imported with PROC IMPORT and then statistical analysis was calculated using PROC MIXED on the mean values across samples due to the presence of zero values in the calculated distances. Treatment and time were included as CLASS variables, and the MODEL was defined as variable = treatment | time to include the main effects and interaction of each factor. Post-hoc testing was computed for specific linear combinations with the ESTIMATE function, and these are shown on the respective figures.

## Supplementary information

Peer Review File

Source data Fig. 1

Source data Fig. 2

Source data Fig. 3

Source data Fig. 4

Source data Fig. 5

Source data Fig. 6

Source data Fig. 7

SD EV Figures

Expanded View Figures

## Data Availability

The datasets and computer code produced in this study are available in the following databases: Raw and processed source data: EMBL-EBI Bioimage Archive S-BIAD3184 (https://doi.org/10.6019/S-BIAD3184). Computer scripts: GitHub (https://github.com/moeghaf/DeepThresh) and (https://github.com/jparkinson375/MatrixIMC) The source data of this paper are collected in the following database record: biostudies:S-SCDT-10_1038-S44320-026-00234-5.
