# Supplementary material for: Extracellular matrix phenotyping by imaging mass cytometry defines distinct cellular matrix environments associated with allergic airway inflammation
Source: Mol Syst Biol. 2026 Jul 20;22(11):1713–46. doi: 10.1038/s44320-026-00234-5 (PMC13728205; doi:10.1038/s44320-026-00234-5)
Supplement: Supplementary file 1 [file 44320_2026_234_MOESM10_ESM.pdf]

Expanded View Figures

Figure EV1. IMC allows identification of different cell populations in the lung.

(**A**) IMC panel antibodies designed to cover a wide range of cell populations and extracellular matrix components found across sub-structures within the lung, such as airways, vessels and alveoli. (**B**) Representative image of an IMC-stained section from a BALB/c PBS-treated animal with the three separate ROIs highlighted (white boxes). Areas were chosen to include at least one small-medium airway and associated structures. Cell segmentation was performed with DeepCell and (**C**) segmentation accuracy was assessed with the CellSegmentationEvaluator pipeline (GitHub), dashed black line shows an equivalent "good" segmentation from a mixed dataset (Chen and Murphy, 2023) with circles representing individual ROIs. Cells were then clustered and annotated based on marker expression. (**D**) Heatmap of a subset of 20,000 cells showing the arcsin-transformed mean expression per cell of the different markers. (**E**) UMAP projection showing cells coloured by their SNN cluster and labelled with their manual annotation. (**F**) Optimal clustering parameters were determined using average silhouette width and neighbourhood purity generated within the Bluster package in R. Manual investigation of K = 40 clusters showed no identifiable improvement over K = 20, so K = 20 was used. (**G**) Multiple dimension scaling (MDS) of cell populations based on average marker expression. (**H**) MDS reduction using the proportions of different cell clusters per ROI for all mice. (**I**) Schematic showing the categorisation and relationship between different levels of the cell populations identified in all mice. (**J**) To broadly characterise the response across mice, the abundance of cells in defined classes from (**I**) were quantified. (**K**) LisaClust regions across all individual ROIs in the dataset. Points represent individual ROIs with the black horizontal bars showing the mean for all ROIs in that group; *n* = 2–4 animals with three corresponding ROIs per sample (**C**) and bars represent the average for that population across all ROIs of that group (**J**).

 © The Author(s)

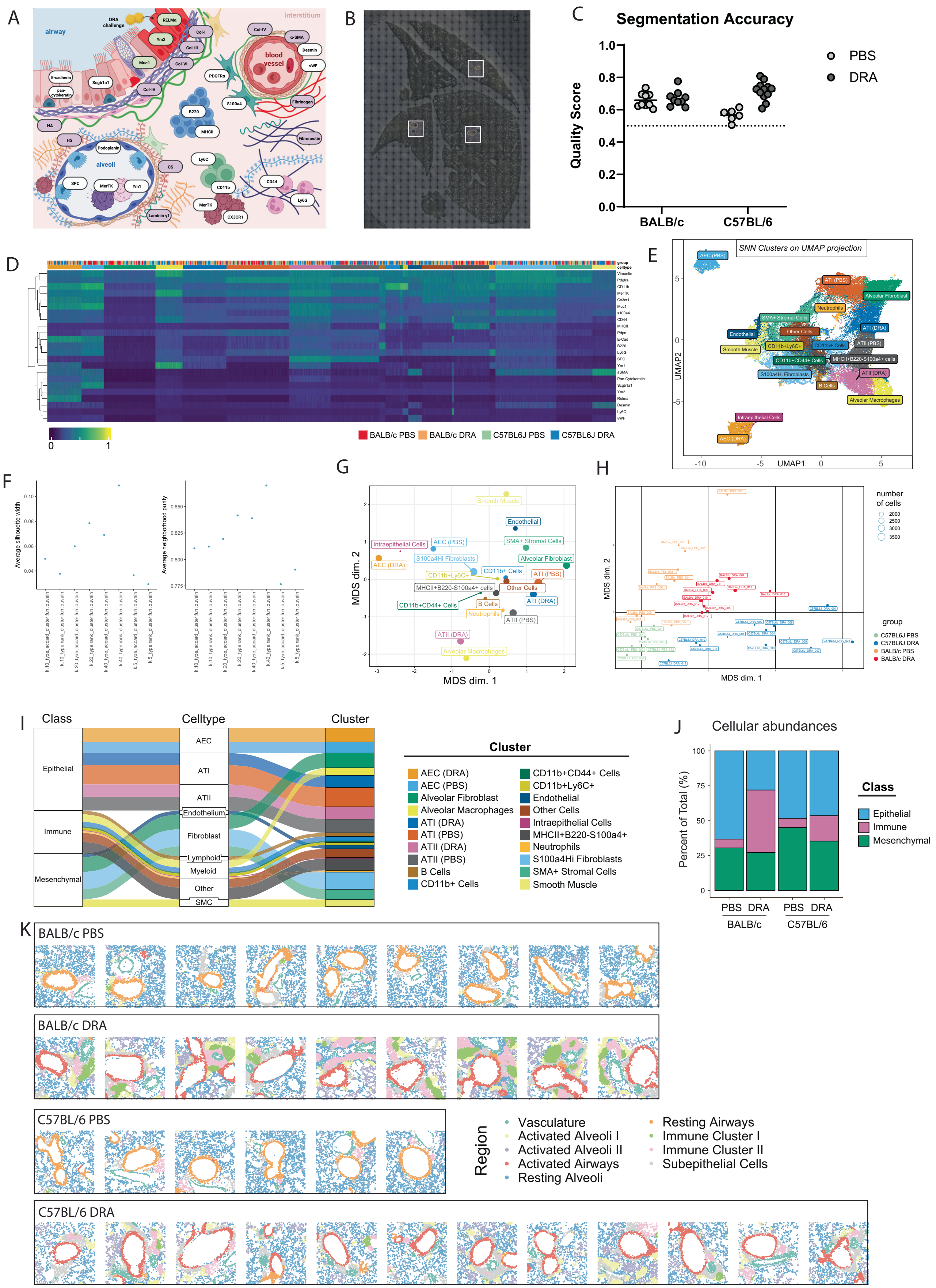
A
airway
interstitium
blood vessel
alveoli
DRA challenge
B
C
Segmentation Accuracy
Quality Score
PBS
DRA
BALB/c
C57BL/6
D
BALB/c PBS
BALB/c DRA
C57BL6J PBS
C57BL6J DRA
E
SNN Clusters on UMAP projection
UMAP1
UMAP2
F
Average silhouette width
Average neighborhood purity
G
MDS dim. 1
MDS dim. 2
H
number of cells
group
C57BL6J PBS
C57BL6J DRA
BALB/c PBS
BALB/c DRA
I
Class
Celltype
Cluster
Epithelial
Immune
Mesenchymal
AEC
ATI
ATII
Endothelium
Fibroblast
Lymphoid
Myeloid
Other
SMC
AEC (DRA)
AEC (PBS)
Alveolar Fibroblast
Alveolar Macrophages
ATI (DRA)
ATI (PBS)
ATII (DRA)
ATII (PBS)
B Cells
CD11b+ Cells
CD11b+CD44+ Cells
CD11b+Ly6C+
Endothelial
Other Cells
Intraepithelial Cells
MHCII+B220-S100a4+
Neutrophils
S100a4Hi Fibroblasts
SMA+ Stromal Cells
Smooth Muscle
J
Cellular abundances
Percent of Total (%)
Class
Epithelial
Immune
Mesenchymal
PBS DRA PBS DRA
BALB/c C57BL/6
K
BALB/c PBS
BALB/c DRA
C57BL/6 PBS
C57BL/6 DRA
Region
Vasculature
Activated Alveoli I
Activated Alveoli II
Activated Airways
Resting Alveoli
Resting Airways
Immune Cluster I
Immune Cluster II
Subepithelial Cells

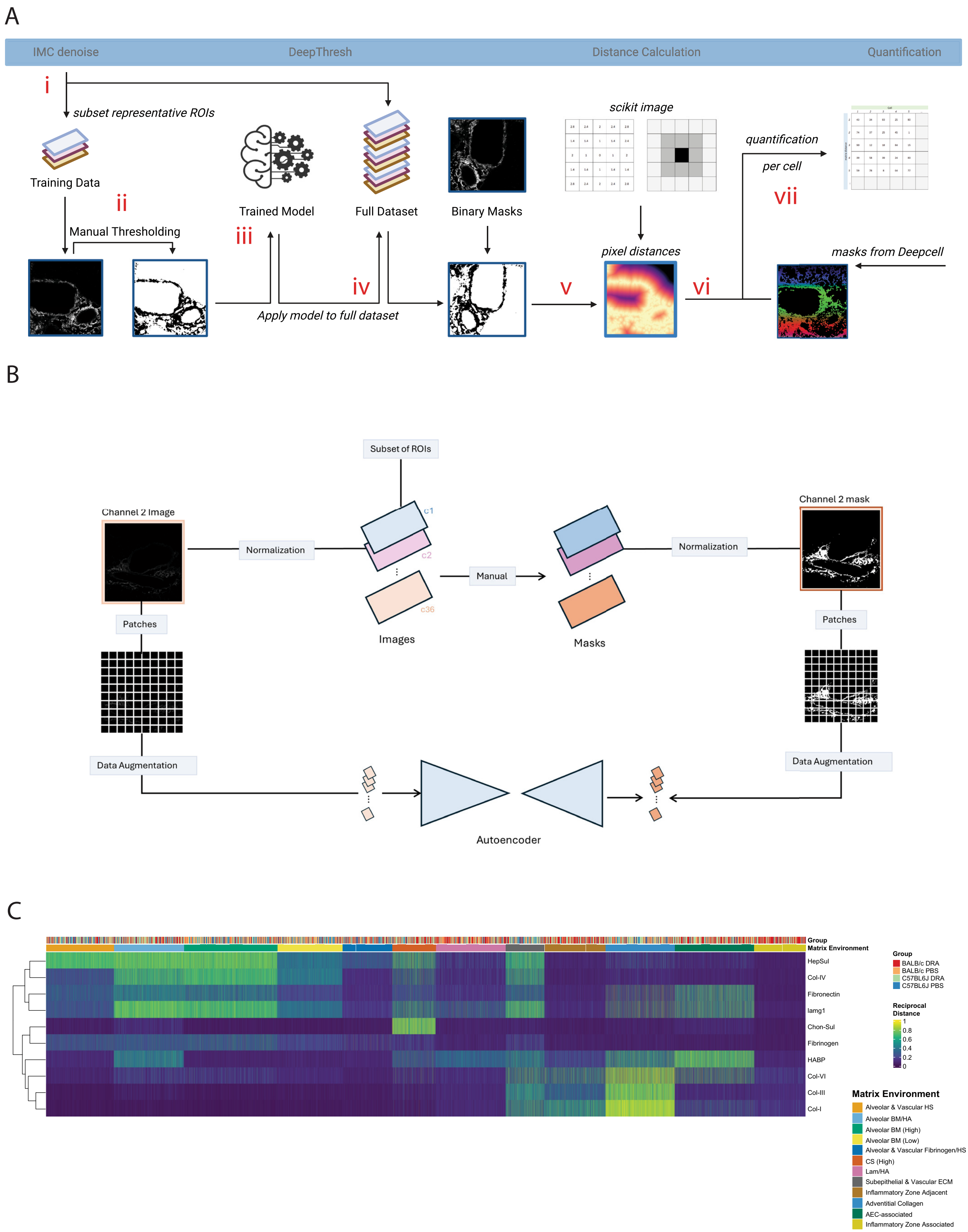
A
IMC denoise
DeepThresh
Distance Calculation
Quantification
i
subset representative ROIs
Training Data
ii
Manual Thresholding
iii
Trained Model
Full Dataset
iv
Apply model to full dataset
Binary Masks
v
scikit image
pixel distances
vi
quantification
per cell
vii
masks from Deepcell
B
Subset of ROIs
Channel 2 Image
Normalization
c1
c2
c36
Images
Manual
Masks
Normalization
Channel 2 mask
Patches
Patches
Data Augmentation
Data Augmentation
Autoencoder
C
Group
Matrix Environment
HepSul
Col-IV
Fibronectin
lamg1
Chon-Sul
Fibrinogen
HABP
Col-VI
Col-III
Col-I
Group
BALB/c DRA
BALB/c PBS
C57BL6J DRA
C57BL6J PBS
Reciprocal Distance
1
0.8
0.6
0.4
0.2
0
Matrix Environment
Alveolar & Vascular HS
Alveolar BM/HA
Alveolar BM (High)
Alveolar BM (Low)
Alveolar & Vascular Fibrinogen/HS
CS (High)
Lam/HA
Subepithelial & Vascular ECM
Inflammatory Zone Adjacent
Adventitial Collagen
AEC-associated
Inflammatory Zone Associated

 © The Author(s)

Figure EV2. DeepThresh for analysis of matrix staining in IMC data.

(**A**) Schematic showing the DeepThresh pipeline for matrix distance quantification. (**i**) Raw stacked tiff files were exported from the IMCdenoise pipeline and split into two groups. (**ii**) One group consisting of a representative sample from each biological group was manually thresholded to generate a binary image showing positive staining as 1 (foreground) and negative staining as 0 (background). (**iii**) A random forest classifier was trained using this data to identify positive regions. (**iv**) The trained classifier was applied to the full dataset to threshold positive staining. (**v**) Thresholded images were used to calculate pixel distances from positive staining using the scikit-image package and images were appended to the original stacked tiff files. (**vi**) Masks were generated with DeepCell using the IMCdenoise data and used to quantify the expression of cell markers, including distance values per cell mask. Data were imported into the normal Steinbock pipeline for further analysis. (**B**) Detailed description of the methodology for thresholding images using DeepThresh. All regions of interest (ROIs) were enhanced using IMCDenoise (Lu et al, 2023). Six ROIs were manually thresholded for use in a semi-automated thresholding approach we refer to as DeepThresh. Four images were used for training, one for validation and one for testing. DeepThresh was built using PyTorch2 (version 2.3) and Pytorch Segmentation Models3. The architecture mimics that of U-Net4 with a ResNet152 encoder5. Adam6 optimiser was applied with a learning rate of 0.0001. All models were trained for 50 epochs. (**C**) Heatmap showing matrix distances across classified matrix environments for all ECM markers evaluated. The scale shows a reciprocal transformation.

© The Author(s)

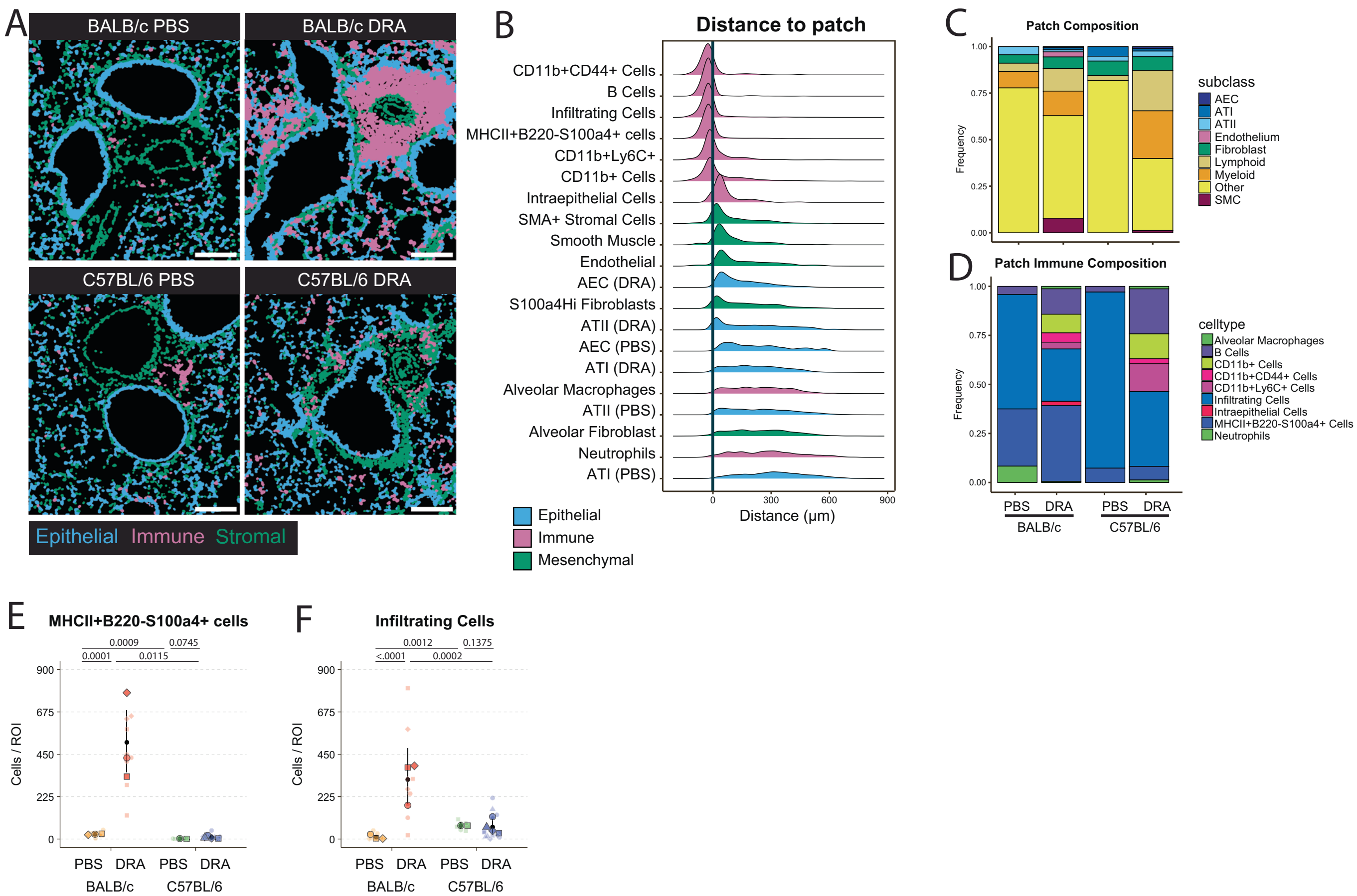

Figure EV3. Spatial cellular composition of the lung during allergic airway pathology.

(**A**) Representative images showing the distribution of different cell classes defined in Fig. 1. (**B**) The mean Euclidean distance (µm) of previously defined SNN cell clusters from their nearest patch. The vertical black line represents 0, the external border of the cell patch. Bars are coloured according to cell class. (**C**) Mean frequency of cell types found across all patches across treatment and mouse strain and (**D**) mean frequency of specific SNN cell clusters within the immune cell class. Quantification of mean cells per ROI of (**E**) $MHCII^{+}B220^{-}S100a4^{+}$ cells and (**F**) infiltrating cells per ROI. (**A**) The same representative sample from each group are shown; scale bars 130 µm. For (**E**, **F**), data points represent individual ROIs with error bars showing the 95% confidence intervals around the mean (black filled circle). $n = 2$–4 animals per group corresponding to 6–12 ROIs per group; mean and error bars are calculated from all ROIs. Statistical significance was calculated with linear mixed-effects models using PROC MIXED in SAS, with *P* values shown on the graph; see Methods for full details.

 © The Author(s)

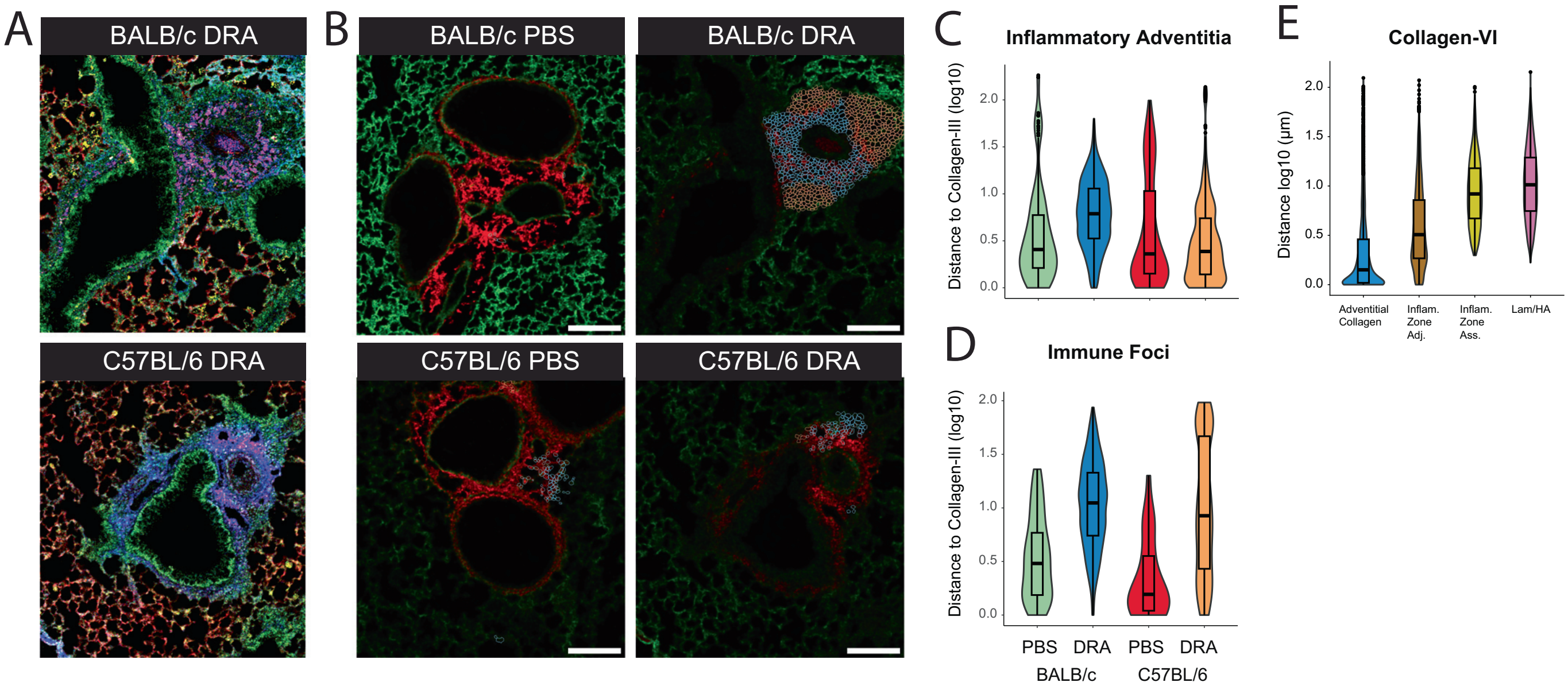

Figure EV4. Immune cell localisation to ECM molecules within the adventitial cuff.

(**A**) Representative images showing zoomed out ROIs shown in 4e (left). (**B**) Representative images showing zoomed out ROI from representative PBS-treated animals and DRA-treated animals, which are also shown in 4e (right). Quantification of the average Euclidean distance of each cell in the (**C**) inflammatory adventitia and (**D**) immune foci lisaClust region to type-III collagen. (**E**) Distance of adventitial matrix environments to collagen VI. For (**A**, **B**), the same representative sample from each group are shown and scale bars are equal to 130 µm. For (**C–E**), graphs are violin plots with overlaid boxplots. The thick central line represents the median with the box showing the interquartile range and lines representing Q1 and Q4, outliers are shown as individual points. $n = 2–4$ animals per group corresponding to 6–12 ROIs per group; mean and error bars are calculated from all ROIs. Statistical significance was calculated with general linear models using PROC GLM in SAS, with *P* values shown on the graph; see Methods for full details. HA hyaluronan. Images from (**A**) have been reused in Fig. 2A.

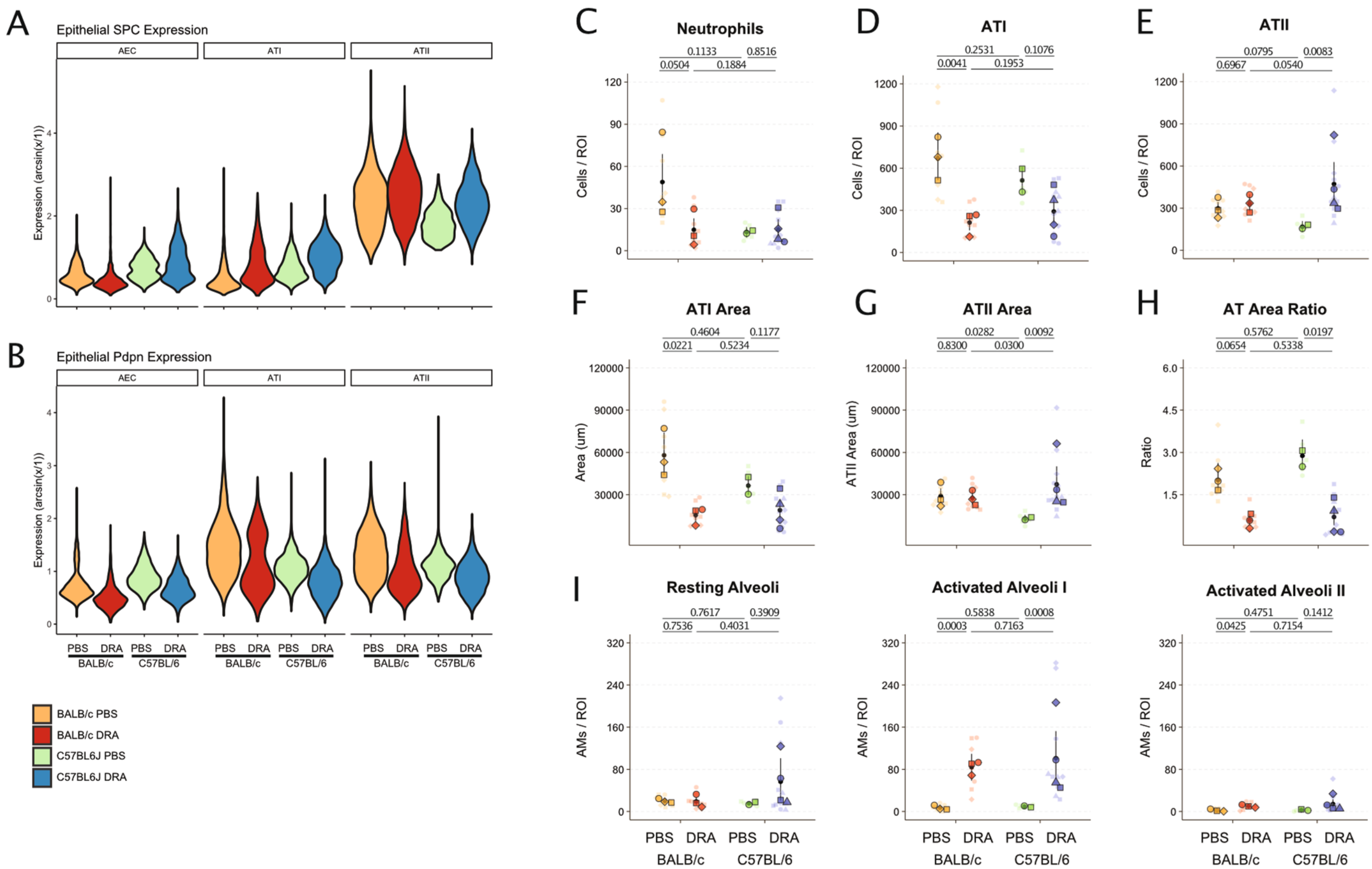

Figure EV5. Phenotyping of the allergic lung epithelium.

Lung epithelial cells express distinct markers, a violin plot showing (**A**) SPC expression and (**B**) Podoplanin (pdpn) expression in epithelial subsets. Quantification of the mean cell number per ROI of (**C**) Neutrophils, (**D**) alveolar type-I (ATI) cells, and (**E**) alveolar type-II (ATII) cells. Quantification of the mean area of (**F**) ATI and (**G**) ATII cells per ROI and (**H**) the ratio of this ATI and ATII area. (**I**) Quantification of the number of alveolar macrophages across different alveolar tissue regions. For (**A**, **B**), the expression of all cells in labelled populations per group are shown in a violin plot. For (**C–I**), smaller background data points represent individual ROIs, larger points show the average per animals, with black error bars showing the 95% confidence intervals around the mean (black filled circle). $n = 2–4$ animals with three corresponding ROIs per sample; mean and error bars are calculated from all ROIs. Statistical significance was calculated with linear mixed-effects models using PROC MIXED in SAS, with *P* values shown on the graph; see Methods for full details.

 © The Author(s)

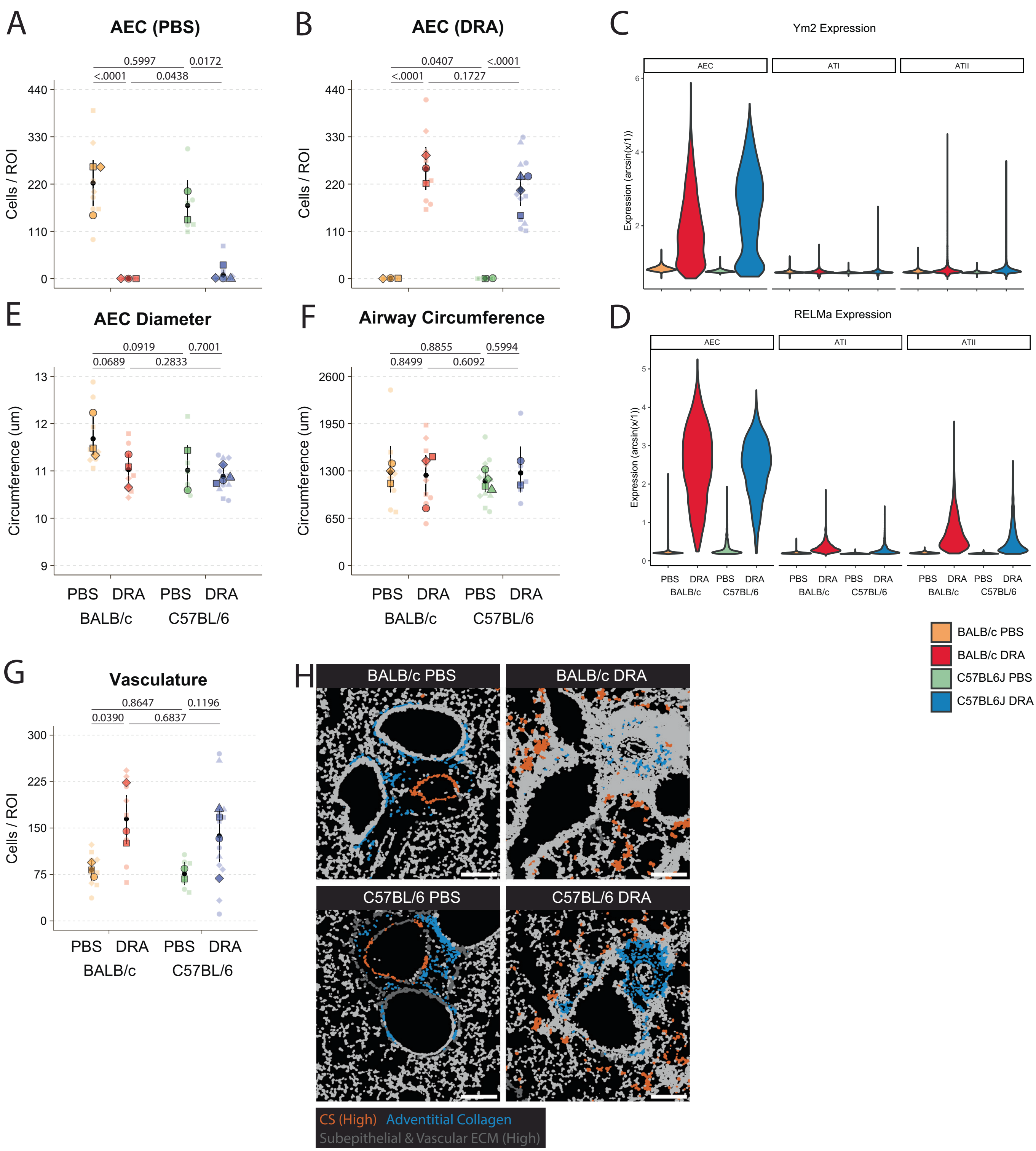

Figure EV6. Airway-associated changes in cells and matrix during AAI.

Quantification of the mean cells per ROI of (**A**) resting AEC (PBS) and (**B**) activated AEC (DRA) per ROI. Violin plots showing the mean expression per cell of canonical epithelial activation markers (**C**) Ym2, and (**D**) RELMα. Quantification of the mean diameter of AECs (**E**) and total airway circumference (**F**) per ROI. (**G**) Quantification of the number of cells in the vasculature lisaClust region per ROI. (**H**) Representative ROIs showing cells in select matrix environments. For panels (**A**, **B**, **E**–**G**) smaller background data points represent individual ROIs, larger points show the average per animals, with black error bars showing the 95% confidence intervals around the mean (black filled circle). $n = 2$–4 animals with three corresponding ROIs per sample. For panels (**C**, **D**), violin plots represent the mean expression calculated across the cell mask. $n = 2$–4 animals per group corresponding to 6–12 ROIs per group; mean and error bars are calculated from all ROIs. Statistical significance was calculated with linear mixed-effects models using PROC MIXED in SAS, with *P* values shown on the graph; see Methods for full details.

© The Author(s)

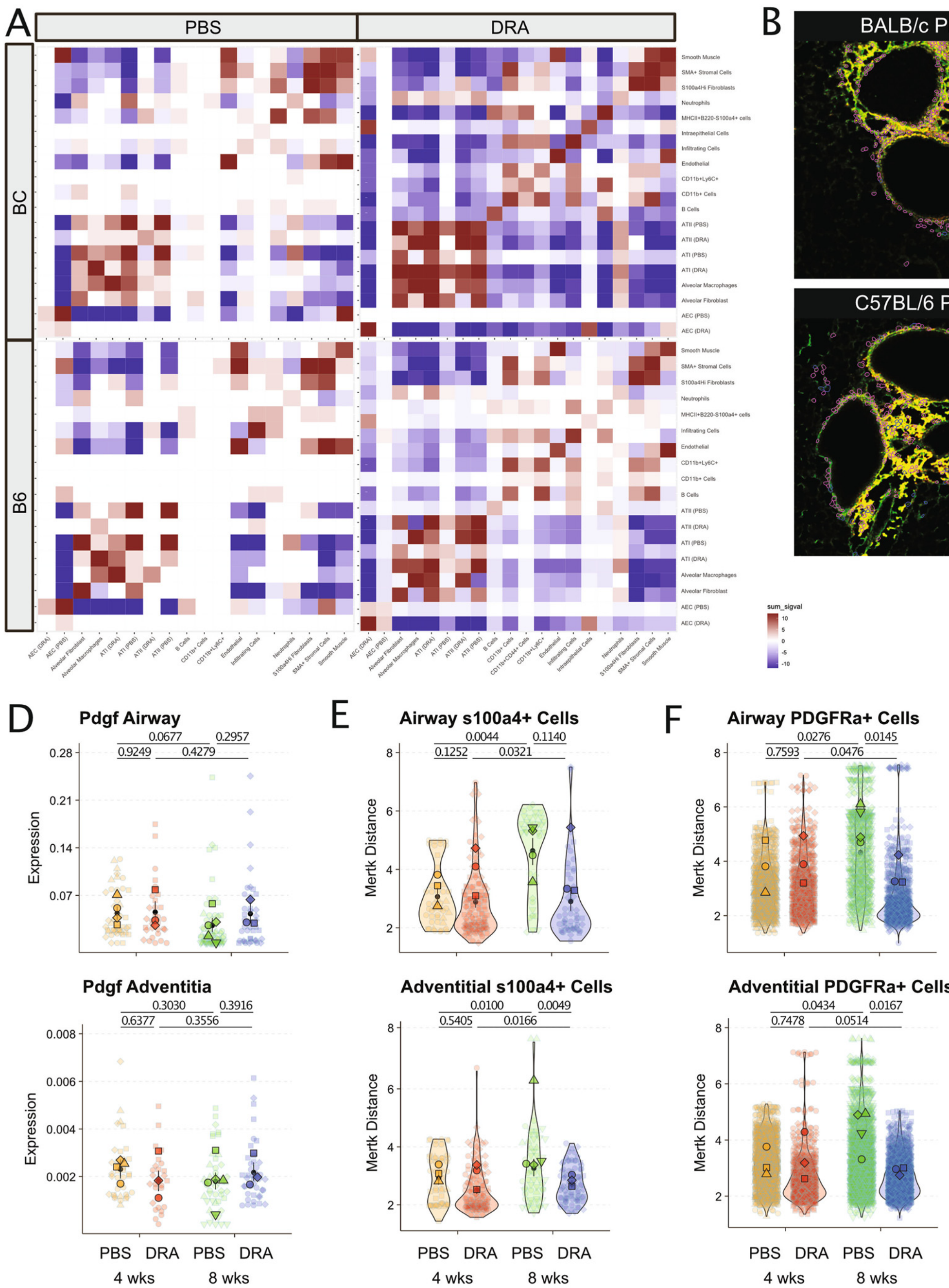

Figure EV7. Macrophages and fibroblasts co-localise in the subepithelial niche during AAI.

(**A**) Further characterisation of the neighbourhood analysis with data split between strains and treatments. (**B**) Representative ROIs (PBS-treated) showing type-I and type-III collagen expression and the localisation of both SMA^{+} stromal cells and S100a4^{+} fibroblasts. Scale bar: 130 μm. (**C**) PCLS immunofluorescent staining of 2-week PBS-challenged BALB/c mice. Scale bar: 50 μm (**D**) Quantification of the number of PDGFRα^{+} cells in the airway and adventitial space. (**E**) Quantification of the distance of S100a4^{+} cells from the closest MertK^{+} staining in the airway and adventitia. (**F**) Quantification of the distance of PDGFRα^{+} cells from the closest MertK^{+} staining in the airway and adventitia. For (**D–F**) graphs are violin plots with overlaid boxplots. The thick central line represents the median, with the box showing the interquartile range and lines representing Q1 and Q4; outliers are shown as individual points. Statistical significance was calculated using PROC MIXED or PROC GLM (for **D–F**) in SAS, with *P* values shown on the graph; see Methods for full details. For panels (**D–F**) data points represent individual ROIs with error bars showing the 95% confidence intervals around the mean (black filled circle). For panels (**E, F**), violin plots represent the mean expression calculated across the cell mask. $n = 2$–4 animals per group corresponding to 6–12 ROIs per group; mean and error bars are calculated from all ROIs. Statistical significance was calculated with linear mixed-effects models using PROC MIXED in SAS; see Methods for full details.

 © The Author(s)
