# Supplementary material for: Extracellular matrix phenotyping by imaging mass cytometry defines distinct cellular matrix environments associated with allergic airway inflammation
Source: Mol Syst Biol. 2026 Jul 20;22(11):1713–46. doi: 10.1038/s44320-026-00234-5 (PMC13728205; doi:10.1038/s44320-026-00234-5)
Supplement: Supplementary file 2 [file 44320_2026_234_MOESM1_ESM.pdf]

molecular systems biology

Spatial ECM mapping defines cellular matrix environments associated with allergic airway pathology

James Parkinson, Morgan Bryant, Mohamed Ghafoor, Rebecca Dodd, Hannah Tompkins, Martin Fergie, Matthew Burgess, Magnus Rattray, and Tara Sutherland

Corresponding author(s): Tara Sutherland (tara.sutherland@abdn.ac.uk)

Review Timeline:

Transfer from Review Commons:	28th Feb 25
Editorial Decision:	10th Mar 25
Revision Received:	10th Apr 26
Editorial Decision:	19th May 26
Revision Received:	25th Jun 26
Accepted:	6th Jul 26

Review Commons

Editor: Poonam Bheda

Transaction Report:

(Note: With the exception of the correction of typographical or spelling errors that could be a source of ambiguity, letters and reports are not edited. Depending on transfer agreements, referee reports obtained elsewhere may or may not be included in this compilation. Referee reports are anonymous unless the Referee chooses to sign their reports.)

Review #1

1. Evidence, reproducibility and clarity:

Evidence, reproducibility and clarity (Required)

In this study, Parkinson et al. investigated lung extracellular matrix using imaging mass cytometry (IMC) in mouse models. Overall, the paper is well-written, and the data are clear, although major points outlined below need to be addressed. In its current form, the paper appears more like a methods-focused study since, to my understanding, no new biological responses are described. The methods employed are very interesting, particularly the extracellular matrix analysis. However, the scope of the study is quite limited, as all the experiments were performed with mouse samples, which are relatively easy to work with, and the cell organisation is simple compared to humans. The authors do not discuss how this analysis pipeline could be applied to human samples. Furthermore, the entire paper relies on imaging mass cytometry, and additional techniques could have been used to confirm some of the observations, especially given the availability of mouse samples.

The introduction mentions the DRA model without providing an explanation of what it involves. Non-specialist readers may not be familiar with this abbreviation, and further clarification should be provided.

In the methods section, it is not mentioned whether the lungs were inflated before tissue collection, which is crucial for preserving normal cellular organization. The authors should clarify whether this was performed.

Figure 1 provides a brief summary of the methods employed in the study but could be enriched with additional information. In its current state, it does not provide meaningful insights beyond what is described in the methods section. It would be helpful if the authors clarified whether the mice used were adults and whether both male and female animals were included. Additionally, they could present examples of the cell segmentation approach with zoomed-in images at the cellular level to illustrate the analysis.

The first set of data in Figure 2 suggests that C57Bl/6 mice did not respond to allergen treatment, as shown by the non-significant increase in cell numbers. The authors should provide evidence that their model induced inflammation through alternative methods, such as assessing eosinophil counts or pathology.

The UMAP representation indicates significant overlap between cell clusters, which raises concerns about the accuracy of cell segmentation. For example, the heatmap in Supplementary Figure 1 shows endothelial cells expressing markers such as VWF, aSMA, Vimentin, and PDGFRα, suggesting that the cell cluster may contain a mixture of endothelial cells, vascular smooth muscle cells, and fibroblasts. Examples of segmented cells should be shown to validate this approach.

It is unclear what Figure 2e represents. If it is simply to show that certain clusters can be grouped together, such as AEC, AT1, and AT2 as epithelial cells, this could be conveyed in a simpler way.
The analysis of extracellular matrix components presented in Figure 3 provides a novel method for studying these acellular structures, which is a challenge in the field. The authors should be commended for their efforts in this area.
However, the parameters investigated in Figures 4-6 do not report any novel findings. While IMC appears to work effectively to analyse these parameters simultaneously, the induction of immune foci and changes in tissue organisation following allergen challenges are already well-documented in both mouse models and human samples.

In Figure 5, the authors show a decrease in neutrophil numbers in challenged mice. This is unexpected, as this model is widely known to induce strong neutrophil recruitment. The authors should clarify this finding and investigate whether neutrophil chemoattractants are increased in these samples.
When analysing epithelial cells, the authors separate AT1 and AT2 cells based on podoplanin expression. However, data in Supplementary Figure 4b suggest that both cell types express similar levels of podoplanin. The authors do not provide statistical validation for the claim that AT1 cells express higher levels. Additionally, as podoplanin is expressed by various cell types, such as lymphatic endothelial cells, additional markers are required to confirm the identity of AT1 cells.
The last set of data in Figure 7 is interesting and shows that immune cells interact with a population of S100a4 fibroblasts. This finding could be expanded further, as CD11b and Ly6C are expressed by a variety of immune cells. The authors should include additional staining to identify the specific cell types involved, such as monocytes, eosinophils, or airway macrophages. Furthermore, the authors should speculate on why these fibroblast regions attract immune cells. Are these regions enriched in chemokines or other factors?
The discussion is engaging but focuses more on methodological aspects than new biological insights. Without mechanistic links, it is challenging to draw meaningful biological conclusions.

2. Significance:

Significance (Required)

The study of Parkinson et al. provides interesting methodological insights into the use of imaging mass cytometry (IMC) to analyse lung architecture following inflammation. The application of multiplex antibody staining will leverage important information related to how tissues are adapting to chronic immune response. Here the authors rely entirely on

mouse models for their studies and compared two lines of WT animals and the same allergen model. This limits the scope of the study, additional timepoints, sex or age would have improved the manuscript.

The applicability of this analysis pipeline to human tissue samples is not discussed, which would significantly enhance the impact of the study. Additionally, complementary techniques, such as flow cytometry or immunohistochemistry, could be used to validate the findings and improve reproducibility. A specialised audience of immunology researchers would be interested by the image analysis approach.

3. How much time do you estimate the authors will need to complete the suggested revisions:

Estimated time to Complete Revisions (Required)

(Decision Recommendation)

Between 3 and 6 months

4. *Review Commons* values the work of reviewers and encourages them to get credit for their work. Select 'Yes' below to register your reviewing activity at [Web of Science Reviewer Recognition Service] (formerly Publons); note that the content of your review will not be visible on Web of Science.

Web of Science Reviewer Recognition

Yes

Review #2

1. Evidence, reproducibility and clarity:

Evidence, reproducibility and clarity (Required)

Summary

Parkinson and colleagues provided a highly intriguing manuscript on spatial resolution of cell-ECM interaction in mouse models of allergic airway inflammation. They used IMH to analyse two common mouse strains for allergic airway inflammation with a human relevant allergen mix. The study implements a novel technique to better segment tissue stainings

(DeepThresh) and modifies existing tools to assess non-cellular seqmentation, ECM or fibrous structures that is. The study identifies region-specific ECM and confirms cellular proximity with canonical cell markers. Furthermore, clear ECM and cellular differences between the two mouse strains are found. The study concludes that this IMH approach is superior to existing methods as it provides a high spatial resolution of ECM protein - cell interaction.

Major:

ECM Isoform Annotation - The manuscript lacks precise annotation of ECM isoforms, particularly for Collagen I, IV, and VI. This impacts the accuracy of reported associations between ECM environments and cellular interactions.
Spatial Annotation Consistency- The manuscript inconsistently defines and annotates ECM environments (e.g., adventitial collagen, subepithelial & vascular ECM), leading to confusion in spatial correlation analyses.
Lack of Supplemental Data- Activated cell types and regions are not clearly defined, and no supplemental data is provided to verify classifications.
DeepThresh Validation - The method for removing staining artifacts via DeepThresh lacks clear validation.
Complexity - Overlapping marker definitions (e.g., CD11b+ cells and infiltrating cells) need clarification for accurate immune cell characterization.

Minor:

Terminology Inconsistency- The manuscript uses inconsistent terminology for ECM components and anatomical regions (e.g., adventitial collagen, immune foci, inflammatory zone).
ROI Mask Coverage - Statistical insignificance in C57BL/6 ROI mask coverage is not addressed.
Figure 3d Labeling- Matrix cluster names do not always match tissue localization.
Spelling Error - "Immunte foci" in Figure 4h.
Figure 6g Correlation Issue- The matrix environment correlation plot does not align with expected cell-ECM interactions.
Color Issues in Figures - ColI and ColIII have the same color in Fig. 3a, making interpretation difficult.
Patch Annotation (Fig. 4i) - The method for defining immune cell patches is unclear.

Detailed review:

Methods: Animal model is suitable for differential analysis of various mouse strain responses to allergic airway inflammation.

Deepthresh matrix thresholding: IMCDenoise is sensitive to clusters of staining artefacts (specks). Please explain how DeepThresh via manual thresholding enables staining artefacts removal/detection. Manual ground truth mapping is common however it is not clear how your approach is performing against another tool. How was manual thresholding controlled (several analysts thresholded same image)?

Antibodies
Collagen IV, stains col4a1 - please correct, as isoforms vary throughout tissue.
Collagen VI, stains col6a1 - isoforms vary in lung tissue, please state correct isoform throughout the document.
Heparan sulfate: Molecular weight?
Collagen I - isoform not defined, please state in methods.

Figure 3 d
As a resultant of the choice of antibodies against some particular isoforms of ECM molecules associations of cells, compartments are correct yet do not comply with all isoforms.
Col4a1 is a basal membrane collagen from blood vessels; the adventitial area and vascular area are high in Col4a1. Other Col4 isoforms are found more frequently in the alveolar regions (col4a5,a6) and the subepithelial membrane. It is of utmost importance to clearly label the correct isoforms throughout the document.

Spelling error in figure 4 h (immunte foci)

ROI mask coverage in C57/6 not significant

Activated cell types/region: This definition is not specified and no supplemental data is provided to see which markers classify such areas/cells. Please provide.

Figure 2i: The cell-specific marker expression is in part already confounded by region. So vasculature or resting airways show no "resting" fibroblasts as their annotation is linked to activation (indicated by S100A4 expression). Anatomic locations such as airways with remodelling are termed "activated" to explain morphological differences which is acceptable given the model chosen. However, some cell type are not given an anatomical

or morpholocial "resting" nomenclature. Only during activation and through location a cell type may aquire e.g. a nomenclature such as "alveolar fibroblast". The correlation blot 2i should provide this basic information. Please explain.

Figure 2h: How do you explain subepithelia to "leak" luminally in C57BL/6 DRA animal? How is an activated airway possible in a Balb/c PBS animal (same for inflammatory adventitia, alveoli)? How is subepithelia adjacent to immune foci and inflammatory adventitia (Balb/c DRA).

Fig 3a: ColI and ColIII have same colour, this makes images not easy to understand please change.
Text for fig 3c: Here it should be mentioned that a cell is used as a proxy locator to the ECM region. Again, in UMAP3b location and ECM molecule a mixed a priori which only can be achieved through proxy loction as in fig 3c or correlation matrix analysis. The UMAP shows ECM molecules in various combinations. Fig3c analysis of anatomic location from images with cell proxies would validate morpho-spatial UMAP annotation. Please make this clear in the manuscript or specify why your approach is superior in its presented format.

Fig 3d: The Matrix Cluster names are in part not correct. Subepithelial & Vascular ECM does not correlate with Vasculature in LisaClust Regions. Also ColIV is not AEC associated, yet subepithelial. No ECM molecule is inflammatory zone associated. Does this indicate cellular density does not allow to distinguish ECM? Also the term "adventitial collagen" is locating to LisaClust Regions Vasculature, Subepithelial Cells, Resting Airways, Infiltrating Cells, Activated Airways. Adventitial per definition of fig. 2g is around blood vessels extending to airways and around it. The adventitial regions are the ECM rich areas after the fibroblasts (as for blood vessels, PMID: 31522963). The definition used in this study therefore generates morphological overlaps between airways and their basolateral regions and blood vessels. Whilst both morphological regions have an adventitia the Matrix cluster assumes from areas to close by this terminology. As a sensitivity analysis I would suggest to reduce the perimeter around blood vessels to the same borderline as seen in airways. If composition remains similar "adventitial collagen" could be a broader term. Alternatively, if adventitia from airway and blood vessel differ these should be separate terms.

Fig 4c: Balb/c and C57bl/6 labels are incorrect (see a,b)
Fig 4h: Cell type "other is highly present in immune foci and inflammatory adventitia but not further classified and not myeloid. This seems either a difficult definition for myeloid or a significant immune population wasn't stained. How is myeloid defined?

Fig 4l has a vast variety of marker combinations some being very specific within the staining panel, others subsummarise entire groups of cells. It would be very helpful to know if the lables are specific and exclusive or if larger clusters exist, that they then subdivide into specific groups (e.g. Infiltrating cells: any of CD11b, CD44, Ly6C vs. B-cells or CD11b+Lys6C). This graph would profit also from either using markers or cell types only. Your marker set is very distinct and limited so per definition it is either a neutrophil or a Lys6C+. Please decide, explain and provide the other graph as supplement.

Fig 5l and sup Fig4i: There is no graph confirming the statement that Ym1 is produced by macrophages. From the graphs in either of the two panels, The AEC are highly associated with Ym1/2 expression or the activated alveoli. Please explain ad amend.

Fig 6g: The correlation plots again show that the matrix environment labels are somewhat confounded. Whilst AEC associated makes perfect sense, adventitial collagen only weakly correlates, yet was part of the adventitial mapping. Cell types like AEC are expected however fibroblasts, especially in resting airways as large constituent cell populations. There are not other, myeloid or lymphoid cells associated with these airways, which under activated conditions seems rather odd. From fig6a it is appearant that the lisaClust has ascribed subepithelial regions to distal parts of the airway separated by blood vessel or parenchyma (C57BL/6 and Balb/c DRA). Also blood vessels are in part other cell types or epithelium (B6 PBS). Is the annotation here the reason for this rather confusing result? Please explain and/or amend.

2. Significance:

Significance (Required)

Strength

Innovative ECM-Immune Interaction Approach- The study integrates extracellular matrix (ECM) phenotyping with immune cell spatial mapping, providing novel insights into allergic airway inflammation
Multiplex Imaging Technology - The use of Imaging Mass Cytometry (IMC) allows high-resolution spatial characterization of both cellular and ECM components.
Strain Analysis - The inclusion of BALB/c and C57BL/6 mice enables differentiation of strain-specific ECM and immune responses.
Deep-Learning-Based ECM Quantification - DeepThresh offers an advanced computational approach for ECM analysis, enhancing accuracy in defining ECM-cell associations.
Comprehensive Tissue Classification- LisaClust clustering facilitates detailed

segmentation of lung microenvironments, improving understanding of localized tissue remodeling.

Limitations

ECM Isoform Inconsistencies - The study lacks precise annotation of ECM isoforms, which affects the accuracy of reported ECM-cell interactions.
Ambiguous Spatial Correlations- Some ECM clusters, such as "adventitial collagen," overlap inconsistently with anatomical regions, making interpretation challenging.
Unvalidated DeepThresh Method - The manuscript does not provide sufficient validation of DeepThresh's ability to remove staining artifacts.
Lack of Supplemental Data- Key activated cell types and regions lack supporting data for classification.

Advance, gap filled

Clearly the next step to improve organ compendia such as the lung cell atlas, spatial protein analysis is warranted. scRNA-Seq in particular for ECM molecules is challenging as these molecules are produced in small quantities or have a very slow turn-over. This study has the potential to provide novel deep learning algorithms to include not only cellular markers but consider larger panels of ECM molecules and their spatial orientation in the lung.

Audience

The manuscript is interdisciplinary located between advanced image analysis with deep learning methods, fundamental lung biology and single cell analysis. The readership would entice molecular biologists, bioinformaticians and basic disease model scientists. The manuscript would appeal to clinician scientists and a broader audience if human tissue pendants could be provided validating the methods and outcomes.

Own Expertise

Translational scientist in the field of chronic lung disease, highly familiar with epithelial cells, mouse models, human cohorts and next generation sequencing and imaging of live single cells.

3. How much time do you estimate the authors will need to complete the suggested revisions:

Estimated time to Complete Revisions (Required)

(Decision Recommendation)

More than 6 months

4. *Review Commons* values the work of reviewers and encourages them to get credit for their work. Select 'Yes' below to register your reviewing activity at Web of Science Reviewer Recognition Service (formerly Publons); note that the content of your review will not be visible on Web of Science.

Web of Science Reviewer Recognition

Yes

Revision Plan

Manuscript number: RC-2024-02789
Corresponding author(s): Tara E, Sutherland

[The "revision plan" should delineate the revisions that authors intend to carry out in response to the points raised by the referees. It also provides the authors with the opportunity to explain their view of the paper and of the referee reports.

The document is important for the editors of affiliate journals when they make a first decision on the transferred manuscript. It will also be useful to readers of the reprint and help them to obtain a balanced view of the paper.

If you wish to submit a full revision, please use our "Full Revision" template. ***It is important to use the appropriate template to clearly inform the editors of your intentions.****]*

1. General Statements [optional]

High-dimensional single-cell imaging is being used more and more to study complex cellular neighborhoods in tissues to better understand development, disease processes, and responses to treatments. However, due to barriers in methodology availability, such datasets completely ignore the extracellular matrix (ECM) environment, even though the ECM is vital for tissue and cell function. One of the main aims of this manuscript was to provide an avenue in which ECM molecules could be meaningfully incorporated into spatial cell datasets. As recognised by both reviewers, we achieved this goal and provide an advanced computation approach to study cell-ECM interactions. Furthermore, we demonstrate the utility of using such analysis methods to discover new cell-cell and cell-matrix relationships that form during chronic allergic airway pathology. Together our data provides insights into the cell networks that can contribute to ECM remodelling around the airways.

2. Description of the planned revisions

We have included a response to the comments addressed by the reviewers below. We appreciate there are a lot of comments but would like to highlight that many of the reviewers' points ask for clarification or text changes rather than additional experiments. In cases where we feel significant text changes, additional experiments and/or data analysis can be added to enhance the manuscript in response to specific points, we have highlighted these in bold below. As several points made by reviewer 2 were repeated through their review, we have only included a response to the initial point below.

Revision Plan

Reviewer 1

In this study, Parkinson et al. investigated lung extracellular matrix using imaging mass cytometry (IMC) in mouse models. Overall, the paper is well-written, and the data are clear, although major points outlined below need to be addressed. In its current form, the paper appears more like a methods-focused study since, to my understanding, no new biological responses are described.
As the reviewer points out a large part of this manuscript is the development of a novel methodology for analyzing the spatial ECM changes in a model of allergic airway inflammation. However, there are several novel responses described in the manuscript. Firstly, differing spatial organisation of immune cells across different mouse strains has not been shown before, particularly in a model of chronic allergic pathology that shares features of severe steroid-resistant asthma in people. Secondly, we show that specific macrophage-fibroblast interactions are occurring in the subepithelial region during DRA-induced allergic airway inflammation. Finally, we integrate all these established and novel findings with detailed spatial analysis of the cellular ECM environment, something which is sorely needed in the field.

The methods employed are very interesting, particularly the extracellular matrix analysis. However, the scope of the study is quite limited, as all the experiments were performed with mouse samples, which are relatively easy to work with, and the cell organisation is simple compared to humans. The authors do not discuss how this analysis pipeline could be applied to human samples. Furthermore, the entire paper relies on imaging mass cytometry, and additional techniques could have been used to confirm some of the observations, especially given the availability of mouse samples.
Whilst we appreciate that the dataset in this study is limited, imaging mass cytometry studies, especially when optimizing reagents, are costly, time consuming, and have limited throughput, not to mention the time required to develop new computational tools for data analysis. Investigating cell-matrix changes in mouse data is vitally important for understanding the mechanistic role of pathways and interactions during disease processes. Whilst we have not provided human datasets in this study, staining, data acquisition and analysis has been performed on FFPE samples, making our pipelines applicable to archival tissue banks. Regardless, we are currently preparing a publication showing the applicability of this technique to human samples. Many ECM components are well conserved between humans and mice and the cellular structure and architecture of the lung shares a lot of similarities. Many papers (PMID: 39437149, 38758780, 38581685, and 38142637) have used this imaging technology in the analysis of human cancer, which shows an even more complicated and dense cellular organisation. Additionally, Steinbock, the main cellular analysis pipeline, is well published in human datasets (PMID: 38758780, 39905080, 39759522, and 39761010). The technology itself is completely species agnostic, so there is no reason to think that there would be issues when applying to humans, other than some differences in the marker expression of certain populations, which is well characterised in many cases.

Revision Plan

The reviewer's comment regarding the use of additional techniques is valid. Firstly, these murine lung pathology samples are derived from the same mouse experiments used in our previous publication (PMID: 33587776) where we have analysed histology, immune mediators and cells using a variety of techniques including flow cytometry and ELISA. We will ensure this point is made clearer in the manuscript. In addition, **for revision we plan to compliment IMC data presented with fluorescent immuno-staining to characterize cell populations in greater resolution and also using 3D precision cut lung slices to better characterize and visualize cell populations of interest in greater depth, directly addressing the reviewer's concerns.**

The introduction mentions the DRA model without providing an explanation of what it involves. Non-specialist readers may not be familiar with this abbreviation, and further clarification should be provided.
As the DRA model has been characterized previously, we provided references in the text in order to save space. However, we agree with the reviewer and will provide this information up front in the introduction to make the manuscript more approachable for a non-specialist.

In the methods section, it is not mentioned whether the lungs were inflated before tissue collection, which is crucial for preserving normal cellular organization. The authors should clarify whether this was performed.
Lungs were inflated prior to tissue collection. We agree that this is important information to include in the methods and we will update the manuscript to reflect this.

Figure 1 provides a brief summary of the methods employed in the study but could be enriched with additional information. In its current state, it does not provide meaningful insights beyond what is described in the methods section. It would be helpful if the authors clarified whether the mice used were adults and whether both male and female animals were included.
We agree with the reviewer. The idea behind this figure was to have an approachable introduction to the manuscript. However, in line with the reviewer's previous comments about focusing more on the biology we will move this to supplementary to keep the importance focused on the biological results. Mouse age and gender were included in the methods of the paper, aligning to the ARRIVE guidelines for reporting animal research. We will clarify that these are adult mice.

Additionally, they could present examples of the cell segmentation approach with zoomed-in images at the cellular level to illustrate the analysis.
This is a great idea and appreciate the reviewer's suggestion. **We will provide maps (with zoomed inserts) of the cell segmentation and cell classification across representative ROIs to show not only the segmentation but to provide an overview of how the cell types localise across the lung.** This addition will also highlight the caveat of IMC around image resolution of 1μm^2 which limits the sensitivity of cell segmentation. **We will discuss such limitations of the technique in**

general in the manuscript in response to this and later reviewer comments.

The first set of data in Figure 2 suggests that C57Bl/6 mice did not respond to allergen treatment, as shown by the non-significant increase in cell numbers. The authors should provide evidence that their model induced inflammation through alternative methods, such as assessing eosinophil counts or pathology.
We know that these exact animals are allergic as their immunological responses were characterized in a previous publication (PMID: 33587776) demonstrating eosinophil counts and cytokine responses measured by flow cytometry. **However, in light of the reviewer's comment, we will add histological images of the lung to this current manuscript.** Such data, together with enhanced expression of RELMα and Ym2 from airway epithelial cells (Sup Fig 6) and the shift from ATI to ATII cells in both C57BL/6 and BALB/c mice after DRA treatment (Fig 5 g) will provide thorough evidence that the DRA model induces allergic airway inflammation and pathology in both mouse strains.

The UMAP representation indicates significant overlap between cell clusters, which raises concerns about the accuracy of cell segmentation. For example, the heatmap in Supplementary Figure 1 shows endothelial cells expressing markers such as VWF, aSMA, Vimentin, and PDGFRα, suggesting that the cell cluster may contain a mixture of endothelial cells, vascular smooth muscle cells, and fibroblasts.
UMAP reductions of IMC do not separate as clearly as those from single cell RNAseq or flow cytometry. This is because the staining intensity from IMC is much lower. Rather than being on a log scale, as for single cell or flow cytometry, the values are much closer to linear. Additionally, due to the limitations in IMC resolution and the fact that we did not have distinct membrane markers in our panel, cell mask generation is often non-optimal. This is particularly evident in regions where cells are in close proximity and where the limitations of, an effectively, two-dimensional 5-micron thick tissue section mean that there can be overlap between one cell and another. Whilst we acknowledge that some populations will be a mix of cell types we are limited by the number of markers we can use in IMC, as well as the limitations mentioned above. We have accounted for this by using methodologies to identify and focus on tissue regions (lisaClust) and correlate changes to differences in these regions rather than single cells per se.

Examples of segmented cells should be shown to validate this approach.
As per the reviewers comment above, we will provide maps (with zoomed inserts) of the cell segmentation and cell classification across representative ROIs to show not only the segmentation but to provide an overview of how the cell types localised across the lung.

It is unclear what Figure 2e represents. If it is simply to show that certain clusters can be grouped together, such as AEC, AT1, and AT2 as epithelial cells, this could be conveyed in a simpler way.

We apologise that the reviewer found Figure 2e confusing. The aim of this figure was to provide a simple diagram to highlight how different classifications of cell types aligned. This was required because there were variations in the specificity of some clusters and to address specific questions it made more sense to analyse cells at a broader level. i.e. merging resting and activated ATI/II cells or grouping specific immune cell clusters into larger groups. We did consider a table, but we did not feel this was a "simpler" way to do it. As it is simply for reference, we will move Figure 2e to supplemental.

The analysis of extracellular matrix components presented in Figure 3 provides a novel method for studying these acellular structures, which is a challenge in the field. The authors should be commended for their efforts in this area.
We thank the reviewer for their comment here. We agree that this is a vital area that needs to be addressed as the immunomatrix becomes ever more important in understanding disease pathogenesis. We developed this novel method to begin to understand key spatial interactions between cells and ECM molecules, something missing from the majority of high-dimensional imaging datasets.

However, the parameters investigated in Figures 4-6 do not report any novel findings. While IMC appears to work effectively to analyse these parameters simultaneously, the induction of immune foci and changes in tissue organisation following allergen challenges are already well-documented in both mouse models and human samples.
We disagree with the reviewer on this point. Figure 4 shows that immune cell infiltration in the adventitial cuff is different between BALB/c and C57BL/6 mice. This is a new discovery and provides nuance to our previously published data (PMID: 33587776), which showed that in the bronchoalveolar lavage from these same mice there were no differences in immune cell populations at these chronic time points. Therefore, analysis of lavage cells or lung histology in isolation does not provide a full picture of allergic immune responses.
Figure 5 shows neutrophils localised with alveolar macrophages in the alveolar parenchyma in this chronic DRA model completely distinct from the spatial adventitial cuff region occupied by other $CD11b^{+}$ cells. In addition, we show that we can identify perturbations in the alveolar parenchyma by IMC and these correlate with known differences in allergy and asthma such as alterations in ATI/ATII balance, which has also not been shown in this model.
Figure 6 demonstrates that we can identify a tissue region termed "subepithelial cells" which is the site of where remodelling events are known to occur in asthma and allergic pathology. This ECM-rich region is strongly associated with fibroblasts and immune cells which leads in to figure 7 showing that these cells are interacting.
In addition to all of this the main focus of this manuscript is to link these analysis parameters to changes in the ECM environment and we have included this in each of these figures showing how these correlates with allergic changes and how they may be important in understanding these processes. **In response to this reviewer's point, we will highlight and make these novel**

findings clearer within the text of the manuscript.

When analysing epithelial cells, the authors separate AT1 and AT2 cells based on podoplanin expression. However, data in Supplementary Figure 4b suggest that both cell types express similar levels of podoplanin. The authors do not provide statistical validation for the claim that AT1 cells express higher levels. Additionally, as podoplanin is expressed by various cell types, such as lymphatic endothelial cells, additional markers are required to confirm the identity of AT1 cells.
Again, the reviewer is entirely correct here. The cells we have identified are labelled as ATI as a best guess and correlate with ATII cells based on anatomical location – though this is likely shared by some of the populations mentioned by the reviewer. The majority of cells in this population are likely ATIs, as they are localized in the alveolar parenchyma and are cells that are not SPC^{+}, though we cannot say for sure without more markers and we were already at the limit of the number of markers that we can run with one IMC panel. It is likely that there are contaminating lymphatic endothelial cells in this cluster. However, these will be a relatively minor population and do not change the main findings presented in the paper. To address this and other comments by the reviewer **we plan to include a limitation section to the discussion that highlights exactly these points for future studies**.

The last set of data in Figure 7 is interesting and shows that immune cells interact with a population of S100a4 fibroblasts. This finding could be expanded further, as CD11b and Ly6C are expressed by a variety of immune cells. The authors should include additional staining to identify the specific cell types involved, such as monocytes, eosinophils, or airway macrophages. Furthermore, the authors should speculate on why these fibroblast regions attract immune cells. Are these regions enriched in chemokines or other factors?
We thank them for this suggestion. To answer this point, **we will conduct immunofluorescent imaging to provide further characterization of these cells in greater depth, as we agree, this will be important to consider**. To best visualize cells and their interactions in this adventitial region, we plan to use 3D precision cut lung slices from PBS versus DRA mice in combination with confocal imaging. This method will allow us to utilize antibodies and markers that do not work in the FFPE sections such as SiglecF (eosinophils), CD11c (DCs, macrophages), CD64 and CD169 (macrophages).

The discussion is engaging but focuses more on methodological aspects than new biological insights. Without mechanistic links, it is challenging to draw meaningful biological conclusions.
We agree that the discussion could be used to reinforce the importance of the biological discoveries we have made (listed previously) in the discussion and will incorporate this into our revision. However, we also believe that it is important to discuss the methodology as this is a novel way to explore ECM-cell interactions in the tissue as highlighted by the reviewer. There are many limitations to using IMC and similar techniques that should be highlighted for future studies so that we can develop better ways of quantifying the ECM environment during disease.

Revision Plan

Significance
The study of Parkinson et al. provides interesting methodological insights into the use of imaging mass cytometry (IMC) to analyse lung architecture following inflammation. The application of multiplex antibody staining will leverage important information related to how tissues are adapting to chronic immune response. Here the authors rely entirely on mouse models for their studies and compared two lines of WT animals and the same allergen model. This limits the scope of the study, additional timepoints, sex or age would have improved the manuscript.
Whilst we appreciate the reviewers points here, we would like to highlight the time involved in generating such datasets, with a lot of careful optimization and experimental design aspects going into each study. Whilst we have also performed staining and analysis using our described method in human FFPE tissue, we are currently looking to further develop analysis tools to assess ECM-cell interactions. Additionally, data acquisition using IMC takes considerable time, and hence it is not feasible run and analysis the number of samples required to address some of the questions proposed by the reviewer.
We believe our manuscript provides novel methodology to analyse ECM environments within spatial datasets, something that no other spatial datasets have explored to date. Furthermore, we provide numerous new biological findings in relation to how cells are organized within the tissue during allergic pathology and propose immune-fibroblast interactions that may be key for driving ECM remodelling in the lung. Integrating these analyses will be key for further understanding the role of the ECM in disease pathogenesis.

The applicability of this analysis pipeline to human tissue samples is not discussed, which would significantly enhance the impact of the study. Additionally, complementary techniques, such as flow cytometry or immunohistochemistry, could be used to validate the findings and improve reproducibility. A specialised audience of immunology researchers would be interested by the image analysis approach.
As mentioned above, this analysis pipeline is easily applied to human samples or any other species as ECM molecule organization is largely conserved across species. Moreover, we have already explored this using human samples. Adding human data to this manuscript is beyond the scope of this manuscript which was aiming to build one of the first methodologies for incorporating the ECM into this kind spatial analysis from the start in order to make biological discoveries. **Regardless, we will add a discussion point on utilizing these pipelines to other species within the discussion of the manuscript.**
Flow cytometry has been published on this model and the exact samples used within this study as mentioned previously (PMID: 33587776), validating some of these findings – we will make this point more clearly in the manuscript. **We do appreciate that it would be good to further expand on some findings presented in the manuscript. As such we will expand our immunostaining (as mentioned above) to give more detail on the infiltrating immune cell populations and their interactions with fibroblasts.**

Revision Plan

<u>Reviewer 2</u>

Major.

ECM Isoform Annotation - The manuscript lacks precise annotation of ECM isoforms, particularly for Collagen I, IV, and VI. This impacts the accuracy of reported associations between ECM environments and cellular interactions.

We thank the reviewer for this excellent comment and pointing this out. **We agree that this is very important and will add this data to the manuscript.** All information was included by reference of the antibody clones. However, it is an important point to make and we will account for this during interpretation of the results.

Spatial Annotation Consistency- The manuscript inconsistently defines and annotates ECM environments (e.g., adventitial collagen, subepithelial & vascular ECM), leading to confusion in spatial correlation analyses.

We are unsure what the reviewer is exactly referring to here. We have maintained a consistent nomenclature for these annotations throughout the manuscript. If the reviewer has an issue with the names we have provided for the regions; names were chosen these to be more informative than just naming them "region 1, 2, 3…". Names in the manuscript were based on taking the lung tissue region and the prominent ECM molecules present. Whilst some level of detail will naturally be lost, we considered this the best way to keep data clear and consistent throughout the manuscript. For example, adventitial collagen describes the region predominantly around the adventitial cuff (fig 3c and d; shown in dark blue) that has high levels of Collagen I, III and VI. Yes, HA, laminin and fibronectin are also expressed, but at much lower levels. Regardless, all the information is present within the figures with readers to observe and make their own interpretations. We are happy to consider alternative names if the reviewer were to provide some guidance on what they thought was more appropriate.

Lack of Supplemental Data- Activated cell types and regions are not clearly defined, and no supplemental data is provided to verify classifications.

DeepThresh Validation - The method for removing staining artifacts via DeepThresh lacks clear validation.

Complexity - Overlapping marker definitions (e.g., CD11b+ cells and infiltrating cells) need clarification for accurate immune cell characterization.

We provide heatmaps in the supplementary data which shows the exact marker expression pattern for all of the clusters we define (Sup Fig 1a). Additionally, we provide graphs showing the cellular contribution and spatial distribution of all the regions we defined with lisaClust (Fig 2h & I; Sup Fig 1d). Most activated cells are a feature of a specific clustered cell type only being present in either PBS or DRA treated animals. However, the features which have led to separation these cell types are available in the heatmaps as mentioned (Sup Fig 1a).

We believe the reviewer may be confused about the purpose of DeepThresh. This algorithm is not for removing staining artifacts. Instead it uses expert annotation of a small training set to generate a method of accurately thresholding images for positive staining in relatively small ROIs which may have diverse structural features with different staining properties. We did not have space in the manuscript to go into this in more detail. **However, we appreciate this may not be as clear as needed for readers, and hence, will provide supplementary data showing some example thresholding alongside the original staining in a new edit of the manuscript.**

$CD11b^+$ and infiltrating cells are not an overlapping population, they were separately clustered by the algorithm, but we take the reviewers point that further characterisation could be done. As mentioned in comments from reviewer 1, there is a limitation in the number of markers we can use in IMC, especially with the number of ECM markers we included. Additionally, there are limitations in the appropriate antibodies (carrier-free) that work in FFPE mouse tissue with the antigen retrieval that we use for good, reliable staining of ECM components. **As such, we will perform additional immunofluorescence staining in 3D precision cut lung slices to better characterize the $CD11b^+$ population to address comments by both reviewers.**

Minor:

Terminology Inconsistency- The manuscript uses inconsistent terminology for ECM components and anatomical regions (e.g., adventitial collagen, immune foci, inflammatory zone).

This point was directly addressed above in “Major” points and appears to be a duplicate comment.

ROI Mask Coverage - Statistical insignificance in C57BL/6 ROI mask coverage is not addressed.

The increase in C57BL/6 mice upon DRA treatment in panel A is not “significant” in the modern sense of the word. However, we would argue that stating it is “not significant” would also be a mistake. We prefer to use p values as an inferential measure of significance in combination with measures such as effect size and variance (PMID: 8465801). We find this more useful considering the vast number of mistakes made when interpreting p values (PMID: 18582619). The importance of not purely relying on p values for clinical research has been reviewed recently here (PMID: 39909800).

Whilst we appreciate the reviewer’s stringent requirement for significance, we do not want to make sweeping statements based off of a p value of 0.07, especially in only one experiment. Many papers have been published on the pitfalls of stringently adhering to $p < 0.05$ (PMID: 32157489) Therefore, we focused the discussion more around the BALB/c increase as this was clearly visible in the images and was backed up by the patch detection and also shows the difference in response between these strains.

Figure 3d Labeling- Matrix cluster names do not always match tissue localization.

We believe this is a duplication of the point in “major” points to address by the reviewer.

Spelling Error - "Immunte foci" in Figure 4h.

We thank the reviewer for pointing this out and will correct this.

Figure 6g Correlation Issue- The matrix environment correlation plot does not align with expected cell-ECM interactions.
We find it hard to comment on this without more detail of the cell-ECM interactions that the reviewer believes should be occurring. We analysed this in an unbiased way, so we have not forced interactions to appear based on our preconceptions. The regions being analysed in Fig 6g are the resting (PBS) and activated (DRA) airways that contain expected cell populations of airway epithelial cells and a low level of fibroblasts, likely from just under the airway epithelial cells. These cell populations align with AEC-associated matrix, laminin and hyaluronan, and adventitial collagen regions. Perhaps the reviewer is questioning why the airways are associated with adventitial collagens? The reason behind this, is due to adventitial cuff residing adjacent to a proportion of all airways, and hence any ECM associated with the adventitial cuff will likely be included in an airway region. However, as mentioned previously there are limitations to this analysis and we are very likely missing finer details due to issues such as resolution which we have discussed within the point-by-point on numerous occasions, **and something we will directly address by adding a limitations section to the discussion of the revised manuscript.**

Color Issues in Figures - ColI and ColIII have the same color in Fig. 3a, making interpretation difficult.
We agree with the reviewer on this point. The issue we had here was that Col-I and Col-III strongly overlap in these images, whilst one was green and one yellow the effect was to make them look the same in the final images. **We will remake these images with clearer colours that better illustrate differences in Col-I and Col-III expression**.

Patch Annotation (Fig. 4i) - The method for defining immune cell patches is unclear.
Patches refers to an approach that is used to identify interconnected groups of similar cell types and is a method that is based off published data (PMID: 35363540). **We will add further method details that explains this process to the revised manuscript.**

Detailed review:
Methods: Animal model is suitable for differential analysis of various mouse strain responses to allergic airway inflammation.
We thank the reviewer for this comment and also agree that the mouse models presented in the manuscript can provide insightful and mechanistic data for investigating human disease.

Deepthresh matrix thresholding: IMCDenoise is sensitive to clusters of staining artefacts (specks). Please explain how DeepThresh via manual thresholding enables staining artefacts removal/detection. Manual ground truth mapping is common however it is not clear how your

approach is performing against another tool. How was manual thresholding controlled (several analysts thresholded same image)?

As described for an earlier comment, this is not the function of DeepThresh. Manual annotation for training data was performed by consensus agreement of four independent researchers. In terms of performance against another tool, we are not aware of another tool which performs this function and hence cannot compare. **However, we will add additional data showing the validation metrics for the pipeline to make future comparisons easier.**

Antibodies

Collagen IV, stains col4a1 - please correct, as isoforms vary throughout tissue.

Collagen VI, stains col6a1 - isoforms vary in lung tissue, please state correct isoform throughout the document.

Heparan sulfate: Molecular weight?

Collagen I - isoform not defined, please state in methods.

Figure 3 d

As a resultant of the choice of antibodies against some particular isoforms of ECM molecules associations of cells, compartments are correct yet do not comply with all isoforms.

Col4a1 is a basal membrane collagen from blood vessels; the adventitial area and vascular area are high in Col4a1. Other Col4 isoforms are found more frequently in the alveolar regions (col4a5,a6) and the subepithelial membrane. It is of utmost importance to clearly label the correct isoforms throughout the document.

This relates to the earlier comment made by the reviewer 2. As mentioned, we agree with this key point and will provide this information from the respective antibody clones.

However, we are unable to provide details on the molecular weight of heparan sulfate (HS) as this will vary depending on location/tissue/condition etc. The antibody recognises 10E4 epitope on HS which is found across a wide variety of tissues and species and will recognise many different sizes of HS and even associated molecules such as porcine Heparan. Importantly it is relatively specific, not cross reacting with hyaluronan, keratan sulphate, chondroitin sulphate, or dermatan sulphate which is an issue for certain clones. Whilst the size of the HS is an interesting facet, consideration of changes in sulphation patterns would also be of interest, though these currently cannot be accurately assessed via purely immunostaining-based methodologies and would require more targeted biochemical techniques. In addition to this there are multiple nuances in 10E4 antibody binding (PMID: 15044385 and 11278655) which are interesting, but far beyond the scope of this study. **Although captured in the antibody clone information, we will also ensure this is clear in the methods.**

In relation to Col4 isoforms specifically, often antibodies for the ECM are limited because of their repeating structures it is hard to generate specific antibodies. For collagen IV there many clones for Col4a1, but no specific clones for Col4a3/col4a5 etc, suitable for use in FFPE tissues and metal conjugation required for IMC. Therefore, we were very limited in what was available to detect them

at all. **We will bring this up in the discussion as this is an important point, not just for our data, but also for people attempting to replicate this kind of analysis.**

Spelling error in figure 4 h (immunte foci)
ROI mask coverage in C57/6 not significant
Activated cell types/region: This definition is not specified and no supplemental data is provided to see which markers classify such areas/cells. Please provide.
These appear to be repeated comments, and have been address above.

Figure 2i: The cell-specific marker expression is in part already confounded by region. So vasculature or resting airways show no "resting" fibroblasts as their annotation is linked to activation (indicated by S100A4 expression). Anatomic locations such as airways with remodelling are termed "activated" to explain morphological differences which is acceptable given the model chosen. However, some cell type are not given an anatomical or morpholocial "resting" nomenclature. Only during activation and through location a cell type may aquire e.g. a nomenclature such as "alveolar fibroblast". The correlation blot 2i should provide this basic information. Please explain.
Our staining approach and analysis have only identified certain activated populations as pointed out by the reviewer. Most of the populations that we have identified as “activated” have been identified primarily only in mice administered DRA. The reason that we have not included “resting” and “activated” populations for all cell types is that these clusters were generated using a clustering algorithm based on the cellular markers used within the study. Each cluster was then simply labelled as best we could, using information from marker expression, published biological data, anatomical location, and sample identity (e.g. PBS or DRA).
A caveat to using IMC and other similar imaging techniques is that we will miss certain “flavours” of cell populations because we simply do not have the markers, or scope to include markers, with which to identify these cells. This is partly a problem of appropriate antibody availability, but also for many populations there are no specific markers identified in the literature/databases. Single cell RNAseq has provided deep segmentation of some of these populations, but we (and others) have found that often these make poor antibody choices at the protein immunostaining level.
We are unsure what the reviewer wants adding to plot 2i. This plot shows the cell cluster contribution to different lisaClust defined tissue regions. Hence the presence of alveolar fibroblasts in the resting and activate alveoli region. **However, we will include more discussion on the limitations of markers and identification of specific cell populations in the discussion.**

Figure 2h: How do you explain subepithelia to "leak" luminally in C57BL/6 DRA animal?
We assume the reviewer is referring to the overlap of some grey circles though/over the red airway epithelial cells in the C57BL/6 DRA panel of figure 2h. This figure represents individual cells as circles with the centroid of the circle at the centroid of the cell. Cells are rarely perfect circles and, in this case, it has made it seem like the cell is coming through the airway epithelium, when likely it

is a longer cell that sits directly under it. In addition to this, these are effectively 2-dimensional section (5um thick) that capture as small portion of the lung anatomy, hence occasionally this can result in unusual tissue structures that make no sense in the confines of a 2D section but instead correlate with the larger 3D structure.

How is an activated airway possible in a Balb/c PBS animal (same for inflammatory adventitia, alveoli)?
Activated airway simply describes a region that is showing some evidence of activation markers such as RELMα and/or Ym2 etc. PBS itself, as with any other liquid administered into the lungs, will drive a very low level of inflammation, which is why it is used as a control in the animal model. Therefore, it is not surprising that we see a low number of these “activated” cells in PBS animals vice versa for their activated counterparts in DRA treated animals. This is similar for the other regions mentioned.

How is subepithelia adjacent to immune foci and inflammatory adventitia (Balb/c DRA).
We are somewhat confused by this question. We have termed the region “subepithelia” because it is mostly found under the airway epithelial cells. We found that this region expands during DRA treatment and covers areas close to the immune foci and inflammatory adventitia, hence they are next to each other.
As described above, the names of these regions were chosen for simplicity and to communicate its general features. These, regions were identified by detection of nearby regions of cells with similar cellular compositions and the names we a “best fit”.

Fig 3a: ColI and ColIII have same colour, this makes images not easy to understand please change.
Comment was already addressed above

Text for fig 3c: Here it should be mentioned that a cell is used as a proxy locator to the ECM region.
We apologise that this was unclear for the reviewer. Rather than describing it as using the cell as a proxy locator to the ECM region we find it more accurate to think of it as we are characterizing the matrix environment of the cell i.e. what is the cell close to and what is it far away from. **We will make this clearer in the results by changing the name to cellular matrix environment, rather than matrix environment.**

Again, in UMAP3b location and ECM molecule a mixed a priori which only can be achieved through proxy loction as in fig 3c or correlation matrix analysis. The UMAP shows ECM molecules in various combinations. Fig3c analysis of anatomic location from images with cell proxies would validate morpho-spatial UMAP annotation. Please make this clear in the manuscript or specify why your approach is superior in its presented format.
We struggled to ascertain what the reviewer was referring to here and what edits they were suggesting to the revised manuscript. However, this comment seems to assume that we have used

cellular location as an input to the UMAP in figure 3b, which is untrue. This UMAP (and associated clustering) shows each cell as a dot which is organised based on its distance to the different matrix components. Effectively showing us how different cells cluster based on their cellular matrix environment, with no input of cellular based markers. We are unsure what the reviewer is referring to in relation to the UMAP – as they seem to be describing exactly what figure 3c already is (a spatial map of the UMAP clusters on representative images, which shows that a cells matrix environment does seem to show patterns that align with the general lung anatomy).
Finally, the reviewer asks us to specify why our approach is superior, but we are unclear what the alternative approach is. This methodology is effectively a repurposing of the traditional UMAP and clustering methodology used in many single cell techniques, but instead of applying this to cellular markers we are applying it to a cells matrix environment as quantified by the matrix distances. If the reviewer could clarify this comment we would be happy to revisit it. **As mentioned in the previous comment, we will more clearly describe cellular matrix environments in the revised manuscript and this may also help with the confusion.**

Fig 3d: The Matrix Cluster names are in part not correct. Subepithelial & Vascular ECM does not correlate with Vasculature in LisaClust Regions. Also ColIV is not AEC associated, yet subepithelial.
Respectfully, we completely disagree with the reviewer on this point. In the heatmap (Fig 3d) the Subepithelial & Vascular matrix environment correlates most strongly with the Vasculature and Subepithelial cells as shown by the stronger green-yellow colour in the corresponding cell of the heatmap.
As mentioned previously in response to another comment by this reviewer, there could be many reasons that we are not detecting collagen-IV in the AEC associate cell matrix environment. One likely explanation is that this is too fine for the resolution of IMC (1-micron^2) or it could be that certain subchains are utilised here that are not recognized by the antibody we managed to optimize. Additionally, AEC-associated matrix environment is comprised of both mouse strains and includes higher representation from DRA treated animals. From our previous work (PMID: 33587776), we have shown that Col-IV expression around the AEC is reduced in DRA versus PBS - treated animals.

No ECM molecule is inflammatory zone associated. Does this indicate cellular density does not allow to distinguish ECM?
This is a great point from the reviewer and their explanation is entirely possibly. As mentioned there are huge limitations in the resolution of IMC and so we are likely missing finer matrix structures. There is a huge recruitment of cells within this environment so it could be that we cannot clearly visualise fine ECM structure through this considering we are also looking at a 5-micron thick 2D tissue section. Additionally, cells maybe degrading the ECM in order to infiltrate into the tissue. This is definitely an interesting point to examine in further detail, but would need to be done with a different methodology. **We will aim to look at an ECM molecule and its distribution within the**

inflammatory zone using 3D precision cut lung slices and also immune-staining of tissue sections to see whether we can better resolve this in a revised manuscript.

Also the term "adventitial collagen" is locating to LisaClust Regions Vasculature, Subepithelial Cells, Resting Airways, Infiltrating Cells, Activated Airways. Adventitial per definition of fig. 2g is around blood vessels extending to airways and around it. The adventitial regions are the ECM rich areas after the fibroblasts (as for blood vessels, PMID: 31522963). The definition used in this study therefore generates morphological overlaps between airways and their basolateral regions and blood vessels. Whilst both morphological regions have an adventitia the Matrix cluster assumes from areas to close by this terminology. As a sensitivity analysis I would suggest to reduce the perimeter around blood vessels to the same borderline as seen in airways. If composition remains similar "adventitial collagen" could be a broader term. Alternatively, if adventitia from airway and blood vessel differ these should be separate terms.
Whilst the adventitial cuff does refer to the region immediately around a blood vessel in the lung, these structures are slightly more nuanced as blood vessels normally travel through the lung in close association with an airway. This is true all the way down to the close association with the capillaries and the alveolar spaces where gas exchange occurs. Indeed, previous publications (PMID: 30824323) have shown that these adventitial cuffs extend out from around the contiguous area around the blood vessel and associated airway and these can expand during inflammation (PMID: 24631179). This region is rich in Collagen-I and Collagen-III, as we have shown in this manuscript and previously (PMID: 33587776).
Whilst we agree that there are likely microanatomical niches within this larger structure, our dataset lacks the resolution to study this in more detail. However, as mentioned above we can include matrix markers in our future IF staining to examine this region in more detail. The adventitial collagen environment described in this manuscript and beyond, are vital “meet and greet” spots for immune cell infiltrating into the lungs (PMID: 30824323) as well as being sites of iBALT formation (PMID: 24631179)
We are unsure what the reviewer means by “...reduce the perimeter around blood vessels to the same borderline as seen in airways.” We have not defined a manual threshold for the border of the airways. These regions were all defined by SNN clustering and not manual segmentation. Whilst this methodology could be developed we do not believe that this dataset has the resolution to answer this question, as mentioned previously.

Fig 4c: Balb/c and C57bl/6 labels are incorrect (see a,b)
We thank the reviewer for noticing this incorrect labelling and will update this.

Fig 4h: Cell type "other is highly present in immune foci and inflammatory adventitia but not further classified and not myeloid. This seems either a difficult definition for myeloid or a significant immune population wasn't stained. How is myeloid defined?

We define myeloid broadly as CD11b⁺ or alveolar macs. There were certain populations that were not stained, notably T cells. We were unable to have suitable or reliable staining in FFPE tissue with CD90, TCRa/b, CD3e antibodies via IMC. The same was true for Eosinophil markers (SiglecF, Ccr3, EPO, MBP). **The additional experiments we will perform for a revised manuscript (using 3D precision cut lung slices and/or IF staining) should shed further light on these cells. Additionally, as we are not limited by the processing requirements of IMC we can use a wider range of markers.**

Fig 4l has a vast variety of marker combinations some being very specific within the staining panel, others subsummarise entire groups of cells. It would be very helpful to know if the lables are specific and exclusive or if larger clusters exist, that they then subdivide into specific groups (e.g. Infiltrating cells: any of CD11b, CD44, Ly6C vs. B-cells or CD11b+Lys6C). This graph would profit also from either using markers or cell types only. Your marker set is very distinct and limited so per definition it is either a neutrophil or a Lys6C+. Please decide, explain and provide the other graph as supplement.
We apologise that this was not clear to this reviewer. Labels are exclusive and represent the clusters that were identified in figure 2 and are at the finest level of detail that we felt we were able to biologically infer from the data. In terms of the reviewer's first point about infiltrating cells, these are completely separate from the other cell types mentioned. As mentioned in the previous comment, we were simply unable to find working antibodies for some of the common lung populations (a common problem for FFPE sections where antigens are often masked or lost due to fixation and processing) and so are limited to general annotations for these. For the reviewer's second example of Neutrophils vs Ly6C⁺ cells, neutrophils were classified by expression of Ly6G, CD11b⁺, and Ym1 whereas there are many other cell types that express Ly6C, including, but not limited to, dendritic cells, monocytes, eosinophils, and even some T cells.
We believe that the graph in combination with data in Fig 1c and supplementary Fig 1a, already shows what the reviewer is asking for.

Fig 5l and sup Fig4i: There is no graph confirming the statement that Ym1 is produced by macrophages. From the graphs in either of the two panels, The AEC are highly associated with Ym1/2 expression or the activated alveoli. Please explain ad amend.
We assume the reviewer means Fig 5l and sup Fig 5i (as there is no figure sup Fig 4i). Whilst we did not include a graph to show that alveolar macrophages produce Ym1, we did include two references in the text and this has been widely shown in the literature for many years (PMID: 11141507 and 15148607). We are somewhat unclear on the reviewers second point. AEC (airway epithelial cells) can definitely also produce Ym1, though this can often be contentious because of issues with cross-reactivity with its highly homologous sister protein Ym2, which is also produced from airway epithelial cells under Type-2 settings. If the reviewer is referring to AEC (alveolar epithelial cells) then this is true. Activated alveoli are lisaClust regions with lots of alveolar macrophages which was the original statement we made and aligns with sup Fig 5i. Activated

alveoli II have less alveolar macrophages and also have less Ym1, which would correlate though there are other cell types which can make Ym1 as well.

Fig 6g: The correlation plots again show that the matrix environment labels are somewhat confounded. Whilst AEC associated makes perfect sense, adventitial collagen only weakly correlates, yet was part of the adventitial mapping. Cell types like AEC are expected however fibroblasts, especially in resting airways as large constituent cell populations. There are not other, myeloid or lymphoid cells associated with these airways, which under activated conditions seems rather odd. From fig6a it is appearant that the lisaClust has ascribed subepithelial regions to distal parts of the airway separated by blood vessel or parenchyma (C57BL/6 and Balb/c DRA). Also blood vessels are in part other cell types or epithelium (B6 PBS). Is the annotation here the reason for this rather confusing result? Please explain and/or amend.
We are again somewhat confused by this comment. Adventitial collagen only weakly correlates because it is not within the airway epithelial cells, instead it is adjacent in the subepithelial region which is shown in Fig 6j. We are unsure exactly what the reviewer is referring to in terms of “adventitial mapping” but are happy to comment on this if the reviewer can clarify what they mean. We agree with the reviewer that it is somewhat surprising to see so many fibroblasts in the resting and activated airway regions. There is a level of ambiguity here in what lisaClust decides to include in one region vs another. However, what it does show is that there are a large population of fibroblasts around the airway, possibly correlating with peribronchial fibroblasts. We did not observe immune cells in between the airway cells or immediately underneath it. We do not believe this is odd, as from our data it appears that these cells are more likely to be found in the adventitial (including peribronchial as mentioned previously) cuff. Cell are most certainly moving into the airways as shown from the BAL in our previous publication (PMID: 33587776). However, we are unlikely to capture this process in the snapshot of our histology across a relatively small section of the airways covered in our 2D sections.
In regards to the reviewers comment about figure 6a we agree that some of the regions between the airways and blood vessels have been characterised as subepithelia. As mentioned previously we are happy to consider alternative names but have been unable to come up with an alternative that encompasses the cells and spatial region more accurately and clearly. Regardless, the main purpose of these names is to provide simple nomenclature to follow throughout the manuscript and make these types of analyses accessible to all readers. We believe that these are accurately labelled and have provided information about the constituent cell populations that are present within them, making the data and subsequent analysis transparent for others to view and explore. Our data suggests that the adventitial cuff may fulfil multiple roles during DRA-induced inflammation, some of which are more focused on immune cell recruitment and others which may correlate more with the fibroblast rich subepithelial region.
The reviewer is entirely correct to point out that some blood vessels were not entirely annotated. We used vWF to manually separate blood vessels from the adjacent smooth muscle layers, which were not separated by the clustering originally. Notably it appears that veins seem to not separate

as well as arteries suggesting another marker (e.g. CD31) may help with this, though we were limited in what we could include as mentioned previously. As this is only a small effect, which we do not have a way to correct, and blood vessels were not the focus of this manuscript, we have left the annotation as it is with raw data included.

3. Description of the revisions that have already been incorporated in the transferred manuscript

Please insert a point-by-point reply describing the revisions that were already carried out and included in the transferred manuscript. If no revisions have been carried out yet, please leave this section empty.

We have not yet made any revisions to the manuscript, as we would like the advice from editors regarding our point-by-point. However, we have already begun to plan and optimise experiments for characterising cell immune clusters in 3D living lung slices which will complement our current findings.

4. Description of analyses that authors prefer not to carry out

Reviewer 1

In Figure 5, the authors show a decrease in neutrophil numbers in challenged mice. This is unexpected, as this model is widely known to induce strong neutrophil recruitment. The authors should clarify this finding and investigate whether neutrophil chemoattractants are increased in these samples.

This is a keen observation by the reviewer. We were interested in this finding however as it was not the focus of the paper we did not investigate further. In our previous publication we show that there are increased neutrophil numbers in the BAL of these animals (PMID: 33587776) and as mentioned above, we show in figure 5 that neutrophils are found mainly in the alveolar parenchyma. This perhaps means that they are more sensitive to being washed out in the BAL and perhaps there are differences in their "stickiness" in BALB/c and C57BL/6 animals or during DRA-induced allergy. This is in contrast to eosinophils (likely within our $CD11b^+$ cells) which are found in the adventitial cuff, a region is not likely to be captured by BAL wash, though we know that these cells are actively present in the BAL. Overall, though this is an interesting result it was not the focus of this already lengthy paper and is best investigated in another project.

10th Mar 2025

Manuscript Number: MSB-2025-12944-T
Title: Analysis of spatial extracellular matrix-cell environments associated with allergic airway pathology

Dear Dr. Sutherland,

Thank you again for submitting your work to Molecular Systems Biology. We have now discussed your manuscript, the reviews from Review Commons, and your revision plan to address the remaining reviewer comments. We also sent the proposed revision plan also to Reviewer 1, who felt that the revision plan was appropriate - with the exception of the lack of single-cell segmentation that may result in clusters of mixed cell types. We understand that with the current resolution of IMC, this may not be possible. However, given that the reviewers have suggested validation with orthogonal methods that would allow single-cell confirmation of your results, which you have also planned, we would encourage you to also try to validate your cluster analysis (if possible). Please let me know in case you would like to discuss in further detail, I would be happy to schedule a call.

When submitting your revised manuscript, please carefully review the instructions that follow below. We perform an initial quality control of all revised manuscripts before re-review; failure to include requested items will delay the evaluation of your revision.

We require:

1) A .docx formatted version of the manuscript text (including legends for main figures, EV figures and tables). Please make sure that the changes are highlighted to be clearly visible. Alternatively you may choose to submit your manuscript as a LaTeX file.

2) Individual production quality figure files as .eps, .tif, .jpg (one file per figure). For guidance, download the 'Figure Guide PDF' (https://www.embopress.org/page/journal/17574684/authorguide#figureformat).

3) At EMBO Press we ask authors to provide source data for the main figures. Our source data coordinator will contact you to discuss which figure panels we would need source data for and will also provide you with helpful tips on how to upload and organize the files.

4) A .docx formatted letter INCLUDING the reviewers' reports and your detailed point-by-point responses to their comments. As part of the EMBO Press transparent editorial process, the point-by-point response is part of the Peer Review File (PRF), which will be published alongside your paper.

5) A complete author checklist, which you can download from our author guidelines (https://www.embopress.org/page/journal/17574684/authorguide#submissionofrevisions). Please insert information in the checklist that is also reflected in the manuscript. The completed author checklist will also be part of the PRF.

6) Please note that all corresponding authors are required to supply an ORCID ID for their name upon submission of a revised manuscript.

7) It is mandatory to include a 'Data Availability' section after the Materials and Methods. Before submitting your revision, primary datasets produced in this study need to be deposited in an appropriate public database, and the accession numbers and database listed under 'Data Availability'. Please remember to provide a reviewer password if the datasets are not yet public (see https://www.embopress.org/page/journal/17574684/authorguide#dataavailability).

In case you have no data that requires deposition in a public database, please state so in this section as follows: "This study includes no data deposited in external repositories". Note that the Data Availability Section is restricted to new primary data that are part of this study.

8) All Materials and Methods need to be described in the main text using our 'Structured Methods' format, which is required for all research articles. According to this format, the Methods section includes a Reagents and Tools Table (listing key reagents, experimental models, software and relevant equipment and including their sources and relevant identifiers) followed by a Methods and Protocols section describing the methods using a step-by-step protocol format. The aim is to facilitate adoption of the methodologies across labs. Please upload the Reagents and Tools table as a separate document when submitting your revised manuscript. More information on how to adhere to this format as well as a downloadable template (.docx) for the Reagents and Tools Table can be found in our author guidelines:
https://www.embopress.org/page/journal/17444292/authorguide#structuredmethods

An example of a Method paper with Structured Methods can be found here:
https://www.embopress.org/doi/10.15252/msb.20178071.

9) For data quantification: please specify the name of the statistical test used to generate error bars and p-values, the number (n) of independent experiments (specify technical or biological replicates) underlying each data point and the test used to calculate p-values in each figure legend. The figure legends should contain a basic description of n, p-values and the test applied. Graphs must include a description of the bars and the error bars (s.d., s.e.m.). Please provide exact p-values (in either the figure or figure legend).

10) Our journal encourages inclusion of *data citations in the reference list* to directly cite datasets that were re-used and obtained from public databases. Data citations in the article text are distinct from normal bibliographical citations and should directly link to the database records from which the data can be accessed. In the main text, data citations are formatted as follows: "Data ref: Smith et al, 2001" or "Data ref: NCBI Sequence Read Archive PRJNA342805, 2017". In the Reference list, data citations must be labeled with "[DATASET]". A data reference must provide the database name, accession number/identifiers and a resolvable link to the landing page from which the data can be accessed at the end of the reference. Further instructions are available at .

11) We replaced Supplementary Information with Expanded View (EV) Figures and Tables that are collapsible/expandable online. EV Figures should be cited as 'Figure EV1, Figure EV2" etc... in the text and their respective legends should be included in the main text after the legends of regular figures.
- For the figures that you do NOT wish to display as Expanded View figures, they should be bundled together with their legends in a single PDF file called *Appendix*, which should start with a short Table of Content. Appendix figures should be referred to in the main text as: "Appendix Figure S1, Appendix Figure S2" etc.
- Additional Tables/Datasets should be labeled and referred to as Table EV1, Dataset EV1, etc. Legends should be provided in a separate tab in case of .xls files. Alternatively, the legend can be supplied as a separate text file (README) and zipped together with the Table/Dataset file.
See detailed instructions here:
https://www.embopress.org/page/journal/17574684/authorguide#expandedview

12) Author contributions: CRediT has replaced the traditional author contributions section because it offers a systematic machine-readable author contributions format that allows for more effective research assessment. Please remove the Authors Contributions from the manuscript and use the free text boxes beneath each contributing author's name in our system to add specific details on the author's contribution. More information is available in our guide to authors.

13) Disclosure statement and competing interests: We updated our journal's competing interests policy in January 2022 and request authors to consider both actual and perceived competing interests. Please review the policy https://www.embopress.org/competing-interests and update your competing interests if necessary.

14) Every published paper now includes a 'Synopsis' to further enhance discoverability. Synopses are displayed on the journal webpage and are freely accessible to all readers. They include a short stand first (maximum of 300 characters, including space) as well as 2-5 one-sentences bullet points that summarizes the paper. Please write the bullet points to summarize the key NEW findings. They should be designed to be complementary to the abstract - i.e. not repeat the same text. We encourage inclusion of key acronyms and quantitative information (maximum of 30 words / bullet point). Please use the passive voice. Please attach these in a separate file or send them by email, we will incorporate them accordingly.

Please also suggest a striking image or visual abstract to illustrate your article as a PNG file 550 px wide x 300-600 px high.

Please note that these would be the final versions and changes during proofing are usually not allowed.

15) As part of the EMBO Publications transparent editorial process initiative (see our policy here: https://www.embopress.org/transparent-process#Review_Process), Molecular Systems Biology will publish online a Peer Review File (PRF) to accompany accepted manuscripts.
In the event of acceptance, this file will be published in conjunction with your paper and will include the anonymous referee reports, your point-by-point response and all pertinent correspondence relating to the manuscript. Let us know whether you agree with the publication of the PRF and as here, if you want to remove or not any figures from it prior to publication.
Please note that the Author checklist will be published at the end of the PRF.

Molecular Systems Biology has a "scooping protection" policy, whereby similar findings that are published by others during review or revision are not a criterion for rejection. Should you decide to submit a revised version, I do ask that you get in touch after three months if you have not completed it, to update us on the status.

I look forward to receiving your revised manuscript.

Yours sincerely,

Poonam Bheda, PhD
Scientific Editor

Reviewer #1:

Overall, the authors agree to follow the reviewer's suggestions and will provide additional data to strengthen their study. However, a key issue they do not seem inclined to fully address is cellular segmentation. They acknowledge that their method results in "some populations being a mix of cell types," which is a significant concern given the conclusions drawn. I still believe this limitation affects the biological relevance of the study, and I look forward to reviewing the revised version of the paper.

***

Rev_Com_number: RC-2024-02789
New_manu_number: MSB-2025-12944-T
Corr_author: Sutherland
Title: Analysis of spatial extracellular matrix-cell environments associated with allergic airway pathology

Response to reviewers

MSB-2025-12944R

Reviewer 1

Evidence, reproducibility and clarity

In this study, Parkinson et al. investigated lung extracellular matrix using imaging mass cytometry (IMC) in mouse models. Overall, the paper is well-written, and the data are clear, although major points outlined below need to be addressed.

In its current form, the paper appears more like a methods-focused study since, to my understanding, no new biological responses are described. The methods employed are very interesting, particularly the extracellular matrix analysis.

This manuscript does describe the development of novel methodology for analysing spatial ECM changes during allergic airway inflammation filling a current gap in the tools available for spatial cell-matrix analysis. However, there are also novel biological responses described: 1) differing spatial organisation of immune cells across different mouse strains; 2) specific macrophage-fibroblast interactions are occurring in the subepithelial region during DRA-induced allergic airway inflammation; 3) integration of finds with spatial analysis of cellular ECM environment. To further highlight these new biological responses, we have extensively revised the manuscript text, added new analysis data and performed new experiments (highlight below in specific comments).

However, the scope of the study is quite limited, as all the experiments were performed with mouse samples, which are relatively easy to work with, and the cell organisation is simple compared to humans.

We appreciate the dataset was limited, being from a single experiment which we agree was a limitation. Imaging mass cytometry studies when appropriately optimised and controlled, are lengthy and expensive. Therefore, to increase the scope of this manuscript, we have added new data using precision cut lung slices, and immunostaining in FFPE sections from separate mouse experiments and additional timepoints. Additionally, we would note that the cellular organisation of the mouse lung is not straightforwardly simple by comparison to humans. Whilst many differences exist between the species (https://doi.org/10.1093/emph/eow014), we would argue that the relative complexity of mouse versus human lung organisation is not straightforward to define. Many ECM components are well conserved between humans and mice, and the cellular structure and architecture of the lung shares a lot of similarities. Hence, investigating cell-matrix changes in mouse data is vitally important for understanding the mechanistic role of pathways and interactions during disease processes. Whilst we have not analysed human datasets in this manuscript, staining, data acquisition and analysis of mouse tissue has been performed on FFPE samples, making our pipelines applicable to archived tissue banks. Overall, we would argue that this current manuscript provides new understanding on the spatial relationships between cells and matrix that provides insight into studying lung organisation during health versus pathology.

The authors do not discuss how this analysis pipeline could be applied to human samples. Furthermore, the entire paper relies on imaging mass cytometry, and additional techniques could have been used to confirm some of the observations, especially given the availability of mouse samples.

Many published studies (including: https://doi.org/10.1158/0008-5472.CAN-24-1211, https://doi.org/10.1126/sciadv.adj3301, https://doi.org/10.1158/2159-8290.cd-23-1380, https://doi.org/10.1016/j.ebiom.2023.104945) have used imaging mass cytometry in the context of human disease, often cancer where cellular organisation is often complicated and the tissue is cellularly dense. The main analysis pipeline used in this manuscript (Steinbock) has been published in many human datasets (e.g. https://doi.org/10.1126/sciadv.adj3301, https://doi.org/10.1038/s41467-025-56546-x, https://doi.org/10.3389/fimmu.2024.1522582, https://doi.org/10.1097/hc9.0000000000000540) and the homology between ECM components is relatively strong between human and mouse. The technology itself is species agnostic. Therefore, there are no issues applying it to humans, with species-specific marker differences for key cell populations being well documented.

We agree that additional techniques would help support conclusions in the manuscript. Pathology and inflammation measured by histology, flow cytometry, qPCR and ELISA from this same mouse experiment has been published previously, demonstrating the validity of using the DRA allergens as a model of chronic allergic airway pathology (https://doi.org/10.1111/imcb.12448). We have made this point clearer within the methodology section. In addition, in the revised manuscript IMC data has been complemented with fluorescent immune staining in FFPE lung sections (Fig 4, Fig 5 and Fig 7) and 3D lung slices (Fig 3 and Fig 7) to better characterise and visualise key findings; directly addressing the reviewers concerns.

The introduction mentions the DRA model without providing an explanation of what it involves. Non-specialist readers may not be familiar with this abbreviation, and further clarification should be provided.

As the DRA model has been characterized previously, we provided references in the text to save space. However, we agree with the reviewer and provided an expanded introduction on the DRA model (pg 4, paragraph 2 and 3) to make the manuscript more approachable for a non-specialist.

In the methods section, it is not mentioned whether the lungs were inflated before tissue collection, which is crucial for preserving normal cellular organization. The authors should clarify whether this was performed.

Lungs were inflated prior to tissue collection. We agree this is important and it is included in the methods (pg 16).

Figure 1 provides a brief summary of the methods employed in the study but could be enriched with additional information. In its current state, it does not provide meaningful insights beyond what is described in the methods section. It would be helpful if the authors clarified whether the mice used were adults and whether both male and female animals were included.

We agree animal age and sex are important to include. We avoided defining animals as "adult" as this could be differentially interpreted, instead details of mouse age and sex were included in the method's "animals" section, aligning to ARRIVE guidelines for reporting animal research. Figure 1 was used to have an approachable introduction to the manuscript but agree with the reviewer that focusing more on the biology and key findings would enrich the manuscript. Consequently, a lot of the original Fig. 1 has been moved into Sup Fig 1 with Figure 1 now focussed on more concisely describing the different lung tissue niches following analysis of IMC data.

Additionally, they could present examples of the cell segmentation approach with zoomed-in images at the cellular level to illustrate the analysis.

This is an excellent idea, and we appreciate the reviewer's suggestion. Figure 1b includes a map of cell segmentation and cell classification across the representative ROIs provide an overview of cell shape and how specific cell types localise across the lung. Furthermore, throughout the manuscript we have provided zoomed in regions of segmented cells that help highlight key findings (Fig 3g, Fig 4e, Fig 5a, e, o, Fig 6a and j, Fig 7g).

The first set of data in Figure 2 suggests that C57Bl/6 mice did not respond to allergen treatment, as shown by the non-significant increase in cell numbers. The authors should provide evidence that their model induced inflammation through alternative methods, such as assessing eosinophil counts or pathology.

The immunological response of these exact animals was characterised in a previous publication (https://doi.org/10.1111/imcb.12448) demonstrating allergen-induced eosinophilia and pulmonary cytokine responses. Following peer review suggestions, the data throughout the manuscript including total cell numbers (now Fig 1d) is now shown as a superplot (https://doi.org/10.1083/jcb.202001064) allowing visualisation of the averages per mouse and also the different ROIs. In line with this and following discussion

with a professional statistician we have also improved our statistical analysis to use a mixed model which more accurately allows us to incorporate the separate ROIs within an animal into the statistical model, this is detailed in the new statistics section. This new analysis now shows there is a significant change in cell number in C57BL/6 DRA vs PBS mice, albeit to a much lesser degree compared to BALB/c mice. Immune differences between strains have also been extensively discussed previously (https://doi.org/10.1111/imcb.12448). Furthermore, enhanced expression RELMalpha and Ym2 from airway epithelium (Sup Fig 6) and the shift in AT1 and ATII cells (Fig 5a-h) in both C57BL/6 and BALB/c mice after DRA treatment demonstrates that DRA induces allergic airway pathology in both BALB/c and C57BL/6.

The UMAP representation indicates significant overlap between cell clusters, which raises concerns about the accuracy of cell segmentation. For example, the heatmap in Supplementary Figure 1 shows endothelial cells expressing markers such as VWF, aSMA, Vimentin, and PDGFRα, suggesting that the cell cluster may contain a mixture of endothelial cells, vascular smooth muscle cells, and fibroblasts.

As is well recognised in IMC data, staining intensities are closer to a linear scale compared to scRNAseq or flow cytometry, meaning UMAP separation is inherently less discrete. Additional limitations in IMC resolution (1μm^2) means cell mask generation cannot be fully optimised, particularly in regions where cells are in close proximity and where the limitations of a 2D-5μm thick section means cells often overlap. Therefore, we acknowledge that some populations will be a mix of cell types due to limitations in cell masks as well as the number of markers that can be used in IMC. To account for these limitations, our analysis has largely utilised methodologies that identified and focuses on tissue regions (lisaClust) and correlates changes to differences in these regions rather than single cells per se. Furthermore, key biological findings have been further characterised using higher resolution fluorescence imaging of 3D slices or 2D FFPE sections (Fig 3, 4, 5, and 7).

Examples of segmented cells should be shown to validate this approach.

As per the reviewer's comments above, we now provide a map of segmented cells in figure 1b.

It is unclear what Figure 2e represents. If it is simply to show that certain clusters can be grouped together, such as AEC, AT1, and AT2 as epithelial cells, this could be conveyed in a simpler way.

The aim of Fig 2e (now Sup Fig 1i) was to provide a simple diagram to highlight how different classifications of cell types aligned. This was necessary as there were variations in cluster specificity and it made more biological sense to address some key questions at a broader level i.e. merging resting and activated ATI/ATII cells; or grouping specific immune clusters into larger groups. As this figure is for reference, we have moved it to supplemental material and conveyed this meaning in the main text in a simpler way, following the reviewer's suggestion.

The analysis of extracellular matrix components presented in Figure 3 provides a novel method for studying these acellular structures, which is a challenge in the field. The authors should be commended for their efforts in this area.

We thank the reviewer for highlighting the importance of ECM analysis. Whilst single cell transcriptomic and proteomic approaches have provided enormous insight into cellular behaviour during disease, these methods inherently lose spatial context and cannot capture acellular structures such as ECM components. Traditional histological approaches can visualise ECM but are limited in the number of targets that can be simultaneously assessed. High-dimensional spatial imaging therefore represents a critical bridge between these approaches, and its application to ECM analysis remains largely unexplored.

However, the parameters investigated in Figures 4-6 do not report any novel findings. While IMC appears to work effectively to analyse these parameters simultaneously, the induction of immune foci and changes in tissue organisation following allergen challenges are already well-documented in both mouse models and human samples.

We respectfully disagree with the reviewer on this point. Figure 4 (now Fig 3 plus supplementary data) shows that immune infiltration in the adventitial cuff is different between mouse strains. This is a new discovery and provides nuance to our previously published data (https://doi.org/10.1111/imcb.12448), which showed that in the bronchoalveolar lavage from these same mice, there were no differences in immune cell populations at chronic time points. Therefore, analysis of lavage cells or lung histology in isolation will never provide a full picture of the allergic immune response. This leads into Figure 4 which brings in novel ECM analysis within these tissue regions.

Figure 5 identifies perturbations in the alveolar parenchyma both for cell populations and ECM environments. These findings correlate with differences in allergy and asthma such as alterations in ATI/ATII balance, which has not been shown for this model. New fluorescence immunostaining data validates these conclusions.

Figure 6 demonstrates that we can use high-dimensional analysis to identify tissue regions (e.g. sub-epithelial cells) that are the site of remodelling events in asthma and allergic pathology. This ECM-rich region is strongly associated with fibroblast and immune cells which leads into Figure 7, showing that these cells are interacting during remodelling.

Furthermore, the focus of this manuscript is to link analysis parameters to changes in ECM environment and we have included this in each of the figures showing how this correlates with allergic changes and how they may be important in understanding these processes. Regardless, in response to this reviewer's point, we have rewritten the manuscript significantly to ensure these novel findings are clearer and highlighted within the text.

In Figure 5, the authors show a decrease in neutrophil numbers in challenged mice. This is unexpected, as this model is widely known to induce strong neutrophil recruitment. The authors should clarify this finding and investigate whether neutrophil chemoattractants are increased in these samples.

This is a keen observation by the reviewer, and we were interested in this finding. However, as it was not the focus of the paper we did not investigate further. As such this data has now been placed in supplementary data (Sup Fig 5e). As stated by the reviewer, neutrophil numbers are increased in the BAL of DRA animals (https://doi.org/10.1111/imcb.12448), and as mentioned in this manuscript neutrophils are mainly found within the alveolar parenchyma. This perhaps means that neutrophils are more sensitive to being washed out in the lavage and perhaps there are differences in their stickiness in BALB/c and C57BL/6 animals during DRA-induced allergy. This contrasts with eosinophils (likely within our CD11b+ cells of IMC data set) which are found in the adventitial cuff, a region not likely captured by BAL washes, though we know these cells are actively present in the BAL as well, perhaps indicating they are more easily washed out as we did not observe them in the parenchyma in our IMC data. Overall, though this is an interesting result it is not the focus of this already lengthy paper and is best investigated in another project.

When analysing epithelial cells, the authors separate AT1 and AT2 cells based on podoplanin expression. However, data in Supplementary Figure 4b suggest that both cell types express similar levels of podoplanin. The authors do not provide statistical validation for the claim that AT1 cells express higher levels. Additionally, as podoplanin is expressed by various cell types, such as lymphatic endothelial cells, additional markers are required to confirm the identity of AT1 cells.

We agree with the reviewer. The cells were identified and labelled as ATI largely based on positive expression of podoplanin along with pan-cytokeratin and negative or low expression of SPC, in addition to their anatomical location that correlated with ATII cells. As such, the majority of cells in this population are likely ATI's which are a substantial population within the lung. We were at the limit of the number of markers that could be run within one IMC panel, and additional suitable antibodies for labelling ATIs were not available. There are likely some lymphatic endothelial cells within this cluster. However, these will be a relatively minor population. We have performed additional immuno-fluorescence experiments using Rage, an additional

marker of ATI's, with analysis (Fig 5e-h) confirming original findings presented in the manuscript. Hence, the overall conclusions regarding ATI's and ATII's is this manuscript have been confirmed with further studies.

Regardless, to address this and other comments by the reviewer, we have added points throughout the manuscript including the discussion, that highlights limitations and future directions.

The last set of data in Figure 7 is interesting and shows that immune cells interact with a population of S100a4 fibroblasts. This finding could be expanded further, as CD11b and Ly6C are expressed by a variety of immune cells. The authors should include additional staining to identify the specific cell types involved, such as monocytes, eosinophils, or airway macrophages. Furthermore, the authors should speculate on why these fibroblast regions attract immune cells. Are these regions enriched in chemokines or other factors?

As already discussed, IMC allows for high-parameter imaging but is a technique still limited to a set number of markers. Furthermore, our choice of performing IMC on FFPE tissue sections to create methodology relevant to archival tissue, meant that certain antigens are not easily recovered following fixation and paraffin embedding (e.g. Siglec F for eosinophils, alveolar macrophages; CD11c for dendritic cells). However, we agree with this reviewer and felt it was important to provide further characterisation of these immune cells. To best visualise cells within the adventitial region, we used 3D precision cut lung slices from PBS and DRA mice combined with confocal imaging which allows use of a wider range of antibodies. Using this technique, we were able to better show the presence of macrophage populations (CD64+, CD11b+/-, SigF+/-) and eosinophils (CD64- SigF+ CD11b+) in allergic immune foci within BALB/c mice (Fig 3 j-k).

Why immune cells are attracted to this region is of particular interest for future studies. We have included some ideas as to why this may occur within the discussion (pg 15). The adventitial cuff is a complex organ which collects interstitial fluid and is the location of lymphatic vessels reviewed in detail here (https://doi.org/10.1016/j.it.2019.08.002. As such the adventitia functions to monitor tissue signals and subsequently mediate cell recruitment from adjacent blood vessels. Additionally, this cuff may then be important in directing the localisation or activation of infiltrating immune cells as they enter the tissue. This aligns with the identification of ILCs within this area responding to tissue danger signals and initiating effector responses.

The discussion is engaging but focuses more on methodological aspects than new biological insights. Without mechanistic links, it is challenging to draw meaningful biological conclusions.

We agree that the discussion could be used to reinforce the importance of the biological discoveries we have made, as outlined in our response to the preceding comment. However, we also believe it is important to discuss the methodology, its benefits and limitations, particularly as we present a novel way to explore ECM-cell interactions in the tissue (as highlighted by this reviewer). There are many limitations to using IMC and similar techniques that we discuss in brief throughout the manuscript by way of highlighting how future studies can develop better ways of quantifying the ECM environment during disease.

Significance

The study of Parkinson et al. provides interesting methodological insights into the use of imaging mass cytometry (IMC) to analyse lung architecture following inflammation. The application of multiplex antibody staining will leverage important information related to how tissues are adapting to chronic immune response. Here the authors rely entirely on mouse models for their studies and compared two lines of WT animals and the same allergen model. This limits the scope of the study, additional timepoints, sex or age would have improved the manuscript.

We believe this manuscript provides novel methodology to analyse ECM environments within spatial datasets, something that no other spatial datasets have explored to date. Furthermore, we provide numerous new biological findings in relation to how cells are organised within the tissue during allergic

pathology and propose immune-fibroblast interactions that may be key for driving ECM remodelling in the lung. Integrating these analyses will be key for further understanding the role of the ECM in disease pathogenesis.

We appreciate the reviewers points here but would like to highlight the time involved in generating such datasets, with a lot of careful optimisation and experimental design aspects going into each study. Whilst we have also performed staining and analysis using our described method in human FFPE tissue, we are currently looking to further develop analysis tools to assess ECM-cell interactions. Additionally, data acquisition using IMC takes considerable time, and hence it is not feasible to run and analyse the number of samples required to address some of the questions proposed by the reviewer. Many important biological discoveries and methodological advances have been established using defined model systems before broader application across species or conditions; this manuscript is intended to provide that foundational framework. Regardless, following revision and in response to reviewers' comments, we have added additional data that complements IMC studies and adds further strength to our conclusions, including the addition of additional timepoints (Fig 3j and k, Fig 7h-o).

The applicability of this analysis pipeline to human tissue samples is not discussed, which would significantly enhance the impact of the study. Additionally, complementary techniques, such as flow cytometry or immunohistochemistry, could be used to validate the findings and improve reproducibility. A specialised audience of immunology researchers would be interested by the image analysis approach.

We agree that discussing the applicability of this pipeline to human tissue would strengthen the manuscript, as mentioned in the 2nd and last paragraph of the discussion. As highlighted in our response to earlier comments by the reviewer, this pipeline is readily applicable to human or any other species; the technology is species agnostic, ECM components are largely conserved, and the Steinbock analysis pipeline has been widely used in human datasets. We have already begun applying this to human samples, and while including this data is outside the objectives of the current manuscript, we view this as an important next step.

Regarding complementary validation, we have already published flow cytometry data from these exact animals using the same DRA-induced allergy model (https://doi.org/10.1111/imcb.12448, https://doi.org/10.1126/sciimmunol.adk6707), which validates key findings from our IMC data. We have made this point more explicit in the revised methods section. Furthermore, in direct response to this concern, we have added new fluorescent immunostaining in both 2D FFPE sections and 3D precision cut lung slices (Figs 3, 4, 5, 7), providing independent validation of our key findings on immune cell populations and fibroblast interactions.

Reviewer 2

Evidence, reproducibility and clarity

Parkinson and colleagues provided a highly intriguing manuscript on spatial resolution of cell-ECM interaction in mouse models of allergic airway inflammation. They used IMH to analyse two common mouse strains for allergic airway inflammation with a human relevant allergen mix. The study implements a novel technique to better segment tissue stainings (DeepThresh) and modifies existing tools to assess non-cellular seqmentation, ECM or fibrous structures that is. The study identifies region-specific ECM and confirms cellular proximity with canonical cell markers. Furthermore, clear ECM and cellular differences between the two mouse strains are found. The study concludes that this IMH approach is superior to existing methods as it provides a high spatial resolution of ECM protein - cell interaction.

Major:

ECM Isoform Annotation - The manuscript lacks precise annotation of ECM isoforms, particularly for Collagen I, IV, and VI. This impacts the accuracy of reported associations between ECM environments and cellular interactions.

We thank the reviewer for pointing this out and agree it is important information required for full interpretation of the data. All information was included by reference of the antibody clones but we have also added specific isoforms where possible to Table 1-3.

Spatial Annotation Consistency- The manuscript inconsistently defines and annotates ECM environments (e.g., adventitial collagen, subepithelial & vascular ECM), leading to confusion in spatial correlation analyses.

We thank the reviewer for this comment, though we are not sure we have fully understood their concern. We have endeavoured to maintain consistent nomenclature for these spatial annotations throughout the manuscript. Names for annotations were chosen to reflect both anatomical location and dominant ECM composition rather than naming them region 1, 2, 3 etc. For example, "adventitial collagen" defines the matrix environment around the adventitial cuff enriched in collagens VI, I and III (Fig 2c and d). Whilst some detail is naturally lost by naming regions, it was considered the best way to be consistent and maintain clarity throughout the manuscript. Regardless, all the information is present within the figures so readers can observe and make their own interpretations. However, if this does not address the reviewer's specific concern, we would welcome further clarification and are happy to revise accordingly.

Lack of Supplemental Data- Activated cell types and regions are not clearly defined, and no supplemental data is provided to verify classifications.

Heatmaps showing the exact marker expression pattern for all clusters alongside the UMAP is shown in the supplementary data (now Sup Fig 1d & 1e). Additionally, we provided graphs showing the cellular contribution and spatial distribution of all regions defined with lisaClust (Fig 1f, 1g, Sup Fig 1k). Most of the cell clusters defined as "activated" are a feature of a specific clustered cell type only being present in either PBS or DRA animals (e.g. lisaClust region Activated alveoli I is more prominent in DRA-treated mice and features an increased number of alveolar macrophages and ATII (DRA) which express high levels of SPC and activation marker Ym1). The features that have led to separation of these cell types are visible in the heatmaps (Sup Fig 1d).

DeepThresh Validation - The method for removing staining artifacts via DeepThresh lacks clear validation.

We appreciate that our description of DeepThresh may not have been sufficiently clear. This algorithm is not for removing staining artifacts. Instead, it uses expert annotation of a. small training set to generate a method of accurately thresholding images for positive staining in relatively small ROIs which may have diverse structural features with different staining properties. We did not have space in manuscript to go into this in more detail. However, we appreciate this may not have been clear. Therefore, we have added to the text in the manuscript to further explain the thresholding (pg. 7). Further details on DeepThresh are also published on GitHub with examples of the thresholding provided https://github.com/moeghaf/DeepThresh and is referred to in the methods section (pg 20).

Complexity - Overlapping marker definitions (e.g., CD11b+ cells and infiltrating cells) need clarification for accurate immune cell characterization.

CD11b+ cells and infiltrating cells are not an overlapping population, they were separately clustered by the algorithm. However, we agree that further characterisation could be done to improve our understanding of these cells. As mentioned for reviewer 1 comments, IMC is limited by the number of markers we can use, especially seeing as though our aim was to assess the ECM environments alongside cells (also discussed pg 4, and 8 of the manuscript). Additionally, we were limited by appropriate antibodies (carrier-free) that provided validated and reliable staining of antigens in FFPE mouse tissue. Therefore, we have performed additional immunofluorescence staining in 3D and 2D lung slices to better characterise the CD11b+ population (Fig 3 and Fig 7).

Minor:

ROI Mask Coverage - Statistical insignificance in C57BL/6 ROI mask coverage is not addressed.

The increase in C57BL/6 mice upon DRA treatment that was presented in Fig 2a (now Fig 1c) was not significant if only analysed the averages across 3 ROI's per mouse. However, after consultation with a statistician we now take into account heterogeneity across the lung for any given individual mouse. As such all data are presented as "superplots" showing both inter mouse variation and mouse to mouse variation with a significant increase in mask coverage in both BALB/c and C57BL/6 mice.

Spelling Error - "Immunte foci" in Figure 4h.

This has been corrected.

Figure 6g Correlation Issue- The matrix environment correlation plot does not align with expected cell-ECM interactions.

Without further detail on which specific interactions were expected, we have attempted to address what we believe the concern may be. Matrix environments were analysed in an unbiased way, so we have not forced interactions to appear based on our preconceptions. The regions being analysed in Fig 6g (now Fig 6f) are the resting (PBS) and activated (DRA) airways that contained expected cell populations (e.g. airway epithelial cells, fibroblasts that are just under the airway epithelial cells) and align to AEC-associated laminin and hyaluronan, and adventitial collagen matrix environments. If the reviewer is questioning why airways are associated with adventitial collagens, this reflects the anatomical proximity of the adventitial cuff to all airways, meaning the ECM from the cuff will naturally be captured within airway regions. We would welcome further clarification if this does not address the reviewer's specific concerns.

Color Issues in Figures - ColI and ColIII have the same color in Fig. 3a, making interpretation difficult.

Thank you for pointing this out. The colours in this figure (now Fig 2a) have now been adjusted to make the differentiation between matrix molecules including Col-I and Col-III as clear as possible. However, it should be noted that these molecules often have overlapping expression patterns in our dataset making distinguishing them independently difficult.

Patch Annotation (Fig. 4i) - The method for defining immune cell patches is unclear.

Patches refers to an approach that is used to identify interconnected groups of similar cell types and is a method that is based off published data (https://doi.org/10.1126/sciimmunol.abk1692). We have added details that explains this process to the revised manuscript (pg 7).

Detailed review:
Methods: Animal model is suitable for differential analysis of various mouse strain responses to allergic airway inflammation.

We thank the reviewer for this comment and also agree that the mouse model presented in this manuscript can provide insightful and mechanistic data for investigating human disease.

Deepthresh matrix thresholding: IMCDenoise is sensitive to clusters of staining artefacts (specks). Please explain how DeepThresh via manual thresholding enables staining artefacts removal/detection. Manual ground truth mapping is common however it is not clear how your approach is performing against another tool. How was manual thresholding controlled (several analysts thresholded same image)?

As described above in response to the reviewer's earlier comment, this is not the function of DeepThresh, though this 'speck' removal was performed during preprocessing with IMCDenoise. Ground truth data was used, manual annotation for DeepThresh training data was performed by consensus agreement of four independent researchers.

In terms of performance against another tool, we are not aware of another tool which performs this exact function and hence cannot compare this directly. However, basic thresholding was initially attempted using Scikit-image which includes several different thresholding methods including: Isodata, Li, Mean, Minimum, Otsu, Triangle, and Yen (https://scikit-image.org/docs/0.23.x/auto_examples/segmentation/plot_thresholding.html). All of these were sensitive to staining variations across tissue structures across small ROIs and were the reason we developed DeepThresh.

Antibodies
Collagen IV, stains col4a1 - please correct, as isoforms vary throughout tissue.
Collagen VI, stains col6a1 - isoforms vary in lung tissue, please state correct isoform throughout the document.
Heparan sulfate: Molecular weight?
Collagen I - isoform not defined, please state in methods.
Figure 3 d
As a resultant of the choice of antibodies against some particular isoforms of ECM molecules associations of cells, compartments are correct yet do not comply with all isoforms.
Col4a1 is a basal membrane collagen from blood vessels; the adventitial area and vascular area are high in Col4a1. Other Col4 isoforms are found more frequently in the alveolar regions (col4a5,a6) and the subepithelial membrane. It is of utmost importance to clearly label the correct isoforms throughout the document.

This in part relates to the comment made earlier by the reviewer, changes which we have incorporated into the manuscript. In relation to Col-4 isoforms specifically, often antibodies for ECM are limited because of their repeating structures and it is hard to generate specific antibodies. There are many Col4a1 clones, but no specific Col4a3/a5 etc clones suitable for use in FFPE tissues and metal conjugation required for IMC. We agree that this is an important point, not just for our data, but also for people attempting to replicate this kind of analysis and all antibody binding information is included by reference of the specific antibody clones but we have included this were possible.

We are unable to provide details on the molecular weight of heparan sulfate as this will vary depending on location/tissue/condition etc. The antibody recognises 10E4 epitope on HS which is found across a wide variety of tissues and species and will recognise many different sizes of HS and even porcine heparan. Importantly it is relatively specific, not cross-reacting with hyaluronan, keratan sulphate, chondroitin

sulphate, or dermatan sulphate which is an issue for certain antibody clones. Whilst the size of HS is interesting, consideration of changes in sulphation patterns would be also be an interesting avenue, though these currently cannot be accurately assessed via purely immunostaining-based methodologies.

Figure 2i: The cell-specific marker expression is in part already confounded by region. So vasculature or resting airways show no "resting" fibroblasts as their annotation is linked to activation (indicated by S100A4 expression). Anatomic locations such as airways with remodelling are termed "activated" to explain morphological differences which is acceptable given the model chosen. However, some cell type are not given an anatomical or morpholocial "resting" nomenclature. Only during activation and through location a cell type may aquire e.g. a nomenclature such as "alveolar fibroblast". The correlation blot 2i should provide this basic information. Please explain.

Our staining approach and analysis have only identified certain activated populations as highlighted by the reviewer. Most of the populations we identify as "activated" are purely based on their identification in DRA mice and not PBS mice. We have not included resting and activated populations for all cell types, as these clusters were generated using a clustering algorithm based on cellular markers used within the study. Each cluster was then simply labelled as best fit using a combination of marker expression, published biological data, anatomical location, and sample identity (e.g. PBS or DRA).

A caveat to using IMC and other similar imaging techniques is that we will miss certain "flavours" of cell populations because we simply do not have the markers (or scope to include markers) with which to identify these cells. This is partly a problem of appropriate antibody availability, but also for many populations there are no specific markers identified in the literature/databases. Single cell RNAseq has provided deep segmentation of some of these populations, but we (and others) have found that these often make poor antibody choices at the protein/immunostaining level.

We are unsure what the reviewer wants adding to plot Fig 2i (now Fig 1g). This plot shows the cell cluster contribution to different lisaClust defined tissue regions. Hence the presence of alveolar fibroblasts in the resting and activated alveoli region.

Figure 2h: How do you explain subepithelia to "leak" luminally in C57BL/6 DRA animal?

We assume the reviewer is referring to the overlap of some grey circles though/over the red airway epithelial cells in the C57BL/6 DRA panel of figure 2h (now Fig 1f). This figure represents individual cells as circles with the centroid of the circle at the centroid of the cell. Cells are rarely perfect circles and, in this case, it has made it seem like the cell is coming through the airway epithelium, when likely it is a longer cell that sits directly under it. In addition to this, these are effectively 2D sections (5um thick) that capture a small portion of the lung anatomy, hence occasionally this can result in unusual tissue structures that make no sense in the confines of a 2D lung section, but instead correlate with the larger 3D structure.

How is an activated airway possible in a Balb/c PBS animal (same for inflammatory adventitia, alveoli)?

Activated airway simply describes a region that enriched in allergic animals and is showing some evidence of activation markers (e.g. RELM-alpha and/or Ym2). PBS itself, as with any liquid administered into the lungs, will drive a very low level of inflammation, which is why it is used as a control in the animal model. Therefore, it is not surprising that we see a low number of these "activated cells in PBS animals. This is similar for other regions mentioned.

How is subepithelia adjacent to immune foci and inflammatory adventitia (Balb/c DRA).

"Subepithelia" refers to an area mostly found under the airway epithelium cells. This region expands during DRA administration and covers areas close to the immune foci and inflammatory adventitia, hence they are often found next to each other. As described above, the names of these regions were chosen for simplicity and to communicate general features in the data. These regions were identified by detection of nearby regions of cells with similar cellularly composition and the names given as "best fit".

Text for fig 3c: Here it should be mentioned that a cell is used as a proxy locator to the ECM region.

We apologise that this was unclear for the reviewer. Whilst they are correct in their understanding, rather than describing it as using the cell as a proxy locator to the ECM region, we find it more accurate and comprehensible to think of it as we are characterising the matrix environment of that cell; i.e. what is the cell close to and what is it far away from. We have added additional text throughout the manuscript that should make this clearer and we have now referred to matrix environments as cellular matrix environments.

Again, in UMAP3b location and ECM molecule a mixed a priori which only can be achieved through proxy loction as in fig 3c or correlation matrix analysis. The UMAP shows ECM molecules in various combinations. Fig3c analysis of anatomic location from images with cell proxies would validate morpho-spatial UMAP annotation. Please make this clear in the manuscript or specify why your approach is superior in its presented format.

We thank the reviewer for this comment, though we are unsure what the reviewer was referring to here and what edits they were suggesting to the revised manuscript. This comment perhaps assumes that we have used cellular location as an input to the UMAP in figure 3b (now 2b), which is untrue. This UMAP (and associated clustering) shows each cell as a dot which is organised based on its distance to the different matrix components, but no actual spatial localisation (x,y coordinates) was used. Hence, it effectively shows us how different cells cluster based on their cellular matrix environment – a readout that has no input from cellular based markers. Figure 3c (now 2b) is a spatial map of the UMAP clusters on representative images, which shows that a cells matrix environment does seem to have patterns that align with the general lung anatomy.

We would welcome clarification on what alternative approach the reviewer is proposing, as this would help us better address this comment. This methodology is effectively repurposing of the traditional UMAP and clustering methodology used in many single cell techniques, but instead of applying this to cellular markers, we are applying it to a cells matrix environment as quantified by the matrix distances. As mentioned in the previous response, we have more clearly described cellular matrix environments in the main text (pg 7) to help with any confusion.

Fig 3d: The Matrix Cluster names are in part not correct. Subepithelial & Vascular ECM does not correlate with Vasculature in LisaClust Regions. Also ColIV is not AEC associated, yet subepithelial.

We respectfully disagree with this interpretation and would like to clarify the data. The heatmap (now Fig 2d) demonstrates that the subepithelial and vascular matrix environment does correlate with the vasculature and subepithelial cell matrix environment, as indicated by the stronger green-yellow colour in the corresponding cell of the heatmap.

As mentioned previously, there could be many reasons that we are not detecting collagen IV in the AEC associated cell matrix environment. One likely explanation is that this is too fine for the resolution of IMC ($1um^2$), or it could be that certain sub-chains are utilised here that are not recognised by the antibody we managed to optimise.

No ECM molecule is inflammatory zone associated. Does this indicate cellular density does not allow to distinguish ECM?

This is a great point from the reviewer, and their explanation is entirely possible. There are essentially two options:
1. The region is ECM sparse
2. We are not detecting the ECM that is here, and there could be several reasons for this second point.

As mentioned, there are huge limitations in the resolution of IMC and so we will be missing finer matrix structures. As such, the ability to clearly visualise individual matrix fibres, especially in regions of dense immune cell recruitment, analysed in 2D – 5-micron thick tissue sections are possibly limited. However, degradation and remodelling of the ECM would fit with the idea of cells being activated by allergen exposure and then dynamically remodelling and degrading the ECM to infiltrate the tissue.

Another very possible option is that other ECM molecules, not captured by our IMC panel, are more prevalent in these regions, rather than it just being ECM sparse. Further assessment of many more matrix molecules, ideally using a more unbiased method, within these immune cell rich regions would be required before any firm conclusions on this point could be made. Whilst an interesting point for further research, we feel this is beyond the scope of the current paper.

Also the term "adventitial collagen" is locating to LisaClust Regions Vasculature, Subepithelial Cells, Resting Airways, Infiltrating Cells, Activated Airways. Adventitial per definition of fig. 2g is around blood vessels extending to airways and around it. The adventitial regions are the ECM rich areas after the fibroblasts (as for blood vessels, PMID: 31522963). The definition used in this study therefore generates morphological overlaps between airways and their basolateral regions and blood vessels. Whilst both morphological regions have an adventitia the Matrix cluster assumes from areas to close by this terminology. As a sensitivity analysis I would suggest to reduce the perimeter around blood vessels to the same borderline as seen in airways. If composition remains similar "adventitial collagen" could be a broader term. Alternatively, if adventitia from airway and blood vessel differ these should be separate terms.

We thank the reviewer for this constructive suggestion. However, we have not manually defined airways or blood vessels, only annotated regions which were defined by SNN clustering. Reducing the perimeter as suggested is not applicable to our methodology as there is nowhere to do this.

Whilst we agree that there are likely microanatomical niches within the larger adventitial structure, our dataset lacks the resolution to study this in more detail. New data added in Fig 4d, clearly shows the presence of ECM within the cuff when using higher resolution techniques and 3D images. The adventitial collagen environment described in this manuscript and beyond, are vital "meet and greet" spots for immune cell infiltrating into the lungs (https://doi.org/10.1016/j.immuni.2019.02.002) as well as being sites of iBALT formation (https://doi.org/10.1016/j.ajpath.2014.01.021). Previous publications (https://doi.org/10.1016/j.immuni.2019.02.002) have shown that these adventitial cuffs extend beyond the immediate perivascular space to encompass the associated airway (https://doi.org/10.1016/j.ajpath.2014.01.021). However, we are not aware of any work showing differences in matrix expression in airway- vs endothelial-associated adventitia.

Additionally, the adventitial cuffs are slightly more nuanced as arteries normally travel through the lung in close association with an airway. This is true all the way down to the close association with the capillaries and the alveolar spaces where gas exchange occurs and the composition of the matrix in these cuffs may change depending on the level of the lung that they are at. However, a general feature is that this region in rich in collagen-1 and collagen-3 as we have shown in this manuscript and previously (https://doi.org/10.1111/imcb.12448).

Fig 4c: Balb/c and C57bl/6 labels are incorrect (see a,b)

Now Fig 3g – this has been corrected

Fig 4h: Cell type "other is highly present in immune foci and inflammatory adventitia but not further classified and not myeloid. This seems either a difficult definition for myeloid or a significant immune population wasn't stained. How is myeloid defined?

We define myeloid broadly as CD11b+ or alveolar macrophages. There were certain populations that were not stained, notably T cells. Although we attempted to stain for T cells in FFPE tissue with CD90, TCRa/b. CD3e antibodies via IMC, staining was not reliable and there was limited scope for addition of more antibodies in the IMC panel. We have shown previously that perturbing T helper cells (ie Th2 and Th17 cells) does not largely impact on ECM remodelling (https://doi.org/10.1126/sciimmunol.adk6707), hence we maintained our focus on myeloid cells in the lungs.

It was also difficult to specifically stain for eosinophil markers (SiglecF, Ccr3, EPO, MBP). However, new data added to the manuscript utilising confocal imaging confirms our findings with IMC. Whilst Fig 3J shows that eosinophils are present in the immune foci and inflammatory adventitia following DRA, many cells are CD64 and CD11b positive. These data suggest that macrophages are among the key populations accumulating in this ECM-rich region.

Fig 4l has a vast variety of marker combinations some being very specific within the staining panel, others subsummarise entire groups of cells. It would be very helpful to know if the lables are specific and exclusive or if larger clusters exist, that they then subdivide into specific groups (e.g. Infiltrating cells: any of CD11b, CD44, Ly6C vs. B-cells or CD11b+Lys6C). This graph would profit also from either using markers or cell types only. Your marker set is very distinct and limited so per definition it is either a neutrophil or a Lys6C+. Please decide, explain and provide the other graph as supplement.

Labels are exclusive and represent the clusters that were identified in figure 1 and Sup Fig 1. These clusters are at the finest level of detail that we felt we were able to biologically infer from the data. In terms of the reviewer's first point about infiltrating cells, these are completely separate from the other cell types mentioned. As mentioned in the previous comment, we were simply unable to find working antibodies for some common lung populations; a common problem for FFPE sections where antigens are often masked or lost due to fixation and processing with the addition of requiring a carrier-free antibody for metal conjugation for IMC. Therefore, we are limited to general annotations for these cells.

For the reviewers second example of neutrophils vs Ly6C+ cells, neutrophils were classified by expression of Ly6G, CD11b+ and Ym1, whereas there are many other cell types that express Ly6C, including but not limited to certain populations of dendritic cells, monocytes, eosinophils, and even some T cells.

We believe the graph, now Sup Fig 3b, combination with data in Sup Fig 1d and 1e already shows what the reviewer is asking for. However, we have also edited text throughout the manuscript to ensure a clearer message.

Fig 5l and sup Fig4i: There is no graph confirming the statement that Ym1 is produced by macrophages. From the graphs in either of the two panels, The AEC are highly associated with Ym1/2 expression or the activated alveoli. Please explain ad amend.

Relates now to Fig 5n and sup Fig 5h. We did not include a graph to show that alveolar macrophages produce Ym1, but did include two references in the text, as this has been widely shown in the literature for many years (https://doi.org/10.1016/s0002-9440(10)63972-7 and https://doi.org/10.1007/s00418-004-0654-4).

We want to ensure we have fully understood the reviewer's concerns regarding AECs. AEC (airway epithelial cells) can also produce Ym1, though this can be contentious because of issues with cross-reactivity with its highly homologous sibling protein Ym2, which is also produced from airway epithelial cells under type 2 settings. Activated alveoli are a lisaClust region that contains alveolar epithelial cells but also a lot of alveolar macrophages. Activated alveoli II have less alveolar macrophages and also have less Ym1, which would correlate though there are other cell types which can make Ym1 as well, the data showing alveolar macrophage numbers in these different alveolar tissue regions is included in Fig S5i.

Fig 6g: The correlation plots again show that the matrix environment labels are somewhat confounded. Whilst AEC associated makes perfect sense, adventitial collagen only weakly correlates, yet was part of the adventitial mapping. Cell types like AEC are expected however fibroblasts, especially in resting airways as large constituent cell populations. There are not other, myeloid or lymphoid cells associated with these airways, which under activated conditions seems rather odd. From fig6a it is appearant that the lisaClust has ascribed subepithelial regions to distal parts of the airway separated by blood vessel or parenchyma (C57BL/6 and Balb/c DRA). Also blood vessels are in part other cell types or epithelium (B6 PBS). Is the annotation here the reason for this rather confusing result? Please explain and/or amend.

Adventitial collagen only weakly correlates because it is not within the airway epithelial cells, instead it is adjacent in the subepithelial region which is shown in Fig 6j.

We agree with the reviewer that it is somewhat surprising to see so many fibroblasts in the resting and activated airway regions. There is a level of ambiguity here in what lisaClust decides to include in one region vs another. However, what it does show is that there are a large population of fibroblasts around the airway, possibly correlating with peribronchial fibroblasts. We did not observe immune cells in between the airway cells, aside from a very small population of Ly6C+ cells (intraepithelial cells - which could represent intraepithelial mast cells) or immediately underneath it. We do not believe this is odd, as from our data it appears that immune cells are more likely to be found in large numbers around the adventitial (including peribronchial as mentioned previously) cuff. However, cells are indeed recruited to the airways as shown from the BAL in our previous publications (https://doi.org/10.1126/sciimmunol.adk6707, (https://doi.org/10.1111/imcb.12448). It would perhaps make sense to keep the number of immune cells within the alveolar parenchyma to a minimum so as to not interfere with the process of gas exchange. However, cells likely do transit across the alveolar parenchyma but we probably would not be able to capture significant number of cells in this process during the snapshot of our histology across a relatively small section of the airways covered in 2D thin tissue sections.

With regards to the comment about Fig 6a, we agree that some of the regions between the airways and blood vessels have been characterised as subepithelia. We would be happy to consider alternative names but have been unable to come up with something that would encompass the cells and spatial region more accurately and clearly. Regardless, the main purpose of these names is to provide simple nomenclature to follow throughout the manuscript and make these types of analyses accessible to all readers. We believe these are accurately labelled and have provided information about the constituent cell populations that are present within them, making the data and subsequent analysis transparent for others to view and explore. Together, our data suggests that the adventitial cuff may fulfil multiple roles during DRA-induced inflammation, some of which are more focused on immune cell recruitment and others which may correlate more with the fibroblast rich subepithelial region.

The reviewer is entirely correct to point out that some blood vessels were not entirely annotated. We used vWF to manually separate blood vessels from adjacent smooth muscle layers, which were not separated by the clustering originally. Notably, it appears that veins seem to not separate as well as arteries suggesting other markers (e.g. CD31) may help with this, though we were limited at the time in what we could include as mentioned previously. As this is only a small effect, which we do not have a way to correct, and blood vessels were not the focus of this manuscript, we have left the annotation as it is with raw data included.

19th May 2026

Manuscript Number: MSB-2025-12944R
Title: Spatial ECM mapping defines cellular matrix environments associated with allergic airway pathology

Dear Dr. Sutherland,

Thank you for the submission of your revised manuscript to Molecular Systems Biology. We have now received the enclosed reports from the referees that were asked to re-assess it. As you will see the reviewers are now globally supportive and I am pleased to inform you that we will be able to accept your manuscript pending the following final amendments:

1) Please include an institutional email address on the manuscript title page for the corresponding author(s).
2) Authors need to be listed with full first name, instead of just initial (e.g. Mark J Jonson).
3) Please remove the Table of Contents from the manuscript cover page.
4) Please include keywords to max. 5.
5) Please format the Data availability section according to the example below.
"The datasets and computer code produced in this study are available in the following databases:
- Chip-Seq data: Gene Expression Omnibus GSE46748 (https://www.ncbi.nlm.nih.gov/geo/query/acc.cgi?acc=GSE46748)
- Modeling computer scripts: GitHub (https://github.com/SysBioChalmers/GECKO/releases/tag/v1.0)
- [data type]: [full name of the resource] [accession number/identifier] ([doi or URL or identifiers.org/DATABASE:ACCESSION])"
6) Please publicly release the data in Bioimage Archive and update the Data Availability statement to ensure the correct links are included.
7) Please remove the author contributions from the manuscript and specify author contributions in our submission system. CRediT has replaced the traditional author contributions section because it offers a systematic machine-readable author contributions format that allows for more effective research assessment. You are encouraged to use the free text boxes beneath each contributing author's name to add specific details on the author's contribution. More information is available in our guide to authors: https://link.springer.com/journal/44320/submission-guidelines#cms-Manuscript-organisation-and-formatting
8) Please correct the reference citation in the reference list to be alphabetical (not numerical). Where there are more than 10 authors on a paper, only the first 10 should be listed, followed by "et al.". DOIs should only be used for preprints and datasets that have not been published yet. Please check "Author Guidelines" for more information: https://link.springer.com/journal/44320/submission-guidelines#cms-Reference-guidelines
9) Our journal encourages inclusion of *data citations in the reference list* to directly cite datasets that were re-used and obtained from public databases. Data citations in the article text are distinct from normal bibliographical citations and should directly link to the database records from which the data can be accessed. In the main text, data citations are formatted as follows: "Data ref: Smith et al, 2001" or "Data ref: NCBI Sequence Read Archive PRJNA342805, 2017". In the Reference list, data citations must be labeled with "[DATASET]". A data reference must provide the database name, accession number/identifiers and a resolvable link to the landing page from which the data can be accessed at the end of the reference. Further instructions are available at: https://link.springer.com/journal/44320/submission-guidelines#cms-Reference-guidelines
10) In the Methods, please take care of the following:
- The Materials and Methods section should be renamed to "Methods".
- Although you have indicated in the Author Checklist that a statement on blinding has been included in the Methods, we were not able to locate this statement. Please ensure that a statement on whether or not blinding was done is included in the Methods even if no blinding was done.
11) Please remove the Reagents and Tools table from the manuscript, as you have already supplied this as a separate file.
12) Please place individual sections of the manuscript in the following order: Title page - Abstract & Keywords - Introduction - Results - Discussion - Methods - Data Availability - Acknowledgements - Disclosure and Competing Interests Statement - References - (Main Tables with legends if applicable) - Expanded View Figure Legends.
13) For the figures and figure legends, please take care of the following:
- Please ensure that the callouts for all figures are updated in the main manuscript - currently there are callouts for Figure S1 and S7, which need to be corrected (potentially these should be Figure EV1/Figure EV7). A callout is missing for Figure 7G.
- In a routine image check, we note that there has been reuse of the same image between Figure 2A and Figure EV4A. While this is not an issue, it should be mentioned in both Figure legends that the figure has been reused.
- Please note that information related to n is missing in the legend of figure EV1 C
- Please note that the exact p values are not provided in the legends of figures 3F, I; 4D, F, H, G, I; 5C, H; EV6 A, B.
- Please indicate the statistical test used for data analysis in the legends of figures 1C, D; 3B, C, E, F, H, I; 4C, D, F, G, H, I, K, L, M.
14) Please note that funding information should be given in the "Acknowledgements" section (not in its own separate section).
15) Synopsis:
- Synopsis image: Please remove the Graphical Abstract from the main manuscript. The Graphical Abstract (referred to as Synopsis image at EMBO Press) should be uploaded as a separate high-resolution jpeg file 550 pixels wide x 300 pixels high.
- Synopsis text: Please provide a separate word document including a short standfirst (maximum of 300 characters, including spaces) and up to 5 bullet points to summarise the key NEW findings. They should be designed to be complementary to the

abstract - i.e. not repeat the same text. We encourage inclusion of key acronyms and quantitative information (maximum of 30 words / bullet point). Please use the passive voice.
- Please check your synopsis text and image before submission with your revised manuscript. Please be aware that in the proof stage minor corrections only are allowed (e.g., typos).
16) Source Data: Please ensure that a completed Source Data checklist is uploaded as a Related Manuscript File. Source Data should be organized as a single source data file (zipped) per figure for main figures (all EV and/or Appendix figure Source Data can be included in a single folder), with the panels clearly visible in the folder structure instead of a single excel file for all Source Data. e.g. all the Source data files for figure 1 need to be saved in a single folder and this needs to be zipped and then uploaded as "SD figure 1.zip" file.
17) As part of the EMBO Publications transparent editorial process initiative (see our policy here: https://www.embopress.org/transparent-process#Review_Process), Molecular Systems Biology will publish online a Peer Review File (PRF) to accompany accepted manuscripts. This file will be published in conjunction with your paper and will include the anonymous referee reports, your point-by-point response and all pertinent correspondence relating to the manuscript. Let us know whether you agree with the publication of the PRF and as here, if you want to remove or not any figures from it prior to publication. Please note that the Authors checklist will be published at the end of the PRF.
18) After your paper is published, we may promote it on social media. If you have any handles or hashtags for Bluesky you would like included, please let us know.
19) Please provide a point-by-point letter INCLUDING my comments and your detailed responses (as Word file).

I look forward to reading a new revised version of your manuscript as soon as possible.

Yours sincerely,

Poonam Bheda, PhD
Scientific Editor
Molecular Systems Biology

Reviewer #1:

I would like to thank the authors for their revised manuscript. All my comments have been addressed.

Reviewer #2:

Parkinson and colleagues have substantially addressed my main concerns and extensively amended the manuscript, which for I'd like to thank the entire research study team. Some interpretative limitations remain, particularly regarding region nomenclature and causal inference from proximity analyses, but these are now sufficiently acknowledged and do not preclude publication. Again, thank you very much for this high level of revision.

Dear Poonam,

Thank you, the team and the reviewers for all feedback and notifying us about our manuscript MSB-2025012944-T "Extracellular matrix phenotyping by imaging mass cytometry defines cellular matrix environments associated with allergic airway inflammation".

Please find below our responses to your requests below:

1) Please include an institutional email address on the manuscript title page for the corresponding author(s).
This has been updated to show corresponding author and email

2) Authors need to be listed with full first name, instead of just initial (e.g. Mark J Jonson).
All authors now have their first name listed and any middles as initials.

3) Please remove the Table of Contents from the manuscript cover page.
This has been removed.

4) Please include keywords to max. 5.
Key words included below the abstract.

5) Please format the Data availability section according to the example below.
"The datasets and computer code produced in this study are available in the following databases:
- Chip-Seq data: Gene Expression Omnibus GSE46748 (https://www.ncbi.nlm.nih.gov/geo/query/acc.cgi?acc=GSE46748)
- Modeling computer scripts: GitHub (https://github.com/SysBioChalmers/GECKO/releases/tag/v1.0)
- [data type]: [full name of the resource] [accession number/identifier] ([doi or URL or identifiers.org/DATABASE:ACCESSION])"
Wording has been adjusted to follow the example above with accession numbers added.

6) Please publicly release the data in Bioimage Archive and update the Data Availability statement to ensure the correct links are included.
Correct links included with data now released.

7) Please remove the author contributions from the manuscript and specify author contributions in our submission system. CRediT has replaced the traditional author contributions section because it offers a systematic machine-readable author contributions format that allows for more effective research assessment. You are encouraged to use the free text boxes beneath each contributing author's name to add specific details on the author's contribution. More information is available in our guide to authors: https://link.springer.com/journal/44320/submission-guidelines#cms-Manuscript-organisation-and-formatting

I’ve removed all information from the manuscript relating to author contributions.

8) Please correct the reference citation in the reference list to be alphabetical (not numerical). Where there are more than 10 authors on a paper, only the first 10 should be listed, followed by "et al.". DOIs should only be used for preprints and datasets that have not been published yet. Please check "Author Guidelines" for more information: https://link.springer.com/journal/44320/submission-guidelines#cms-Reference-guidelines
These have been updated to the correct citation and reference format.

9) Our journal encourages inclusion of *data citations in the reference list* to directly cite datasets that were re-used and obtained from public databases. Data citations in the article text are distinct from normal bibliographical citations and should directly link to the database records from which the data can be accessed. In the main text, data citations are formatted as follows: "Data ref: Smith et al, 2001" or "Data ref: NCBI Sequence Read Archive PRJNA342805, 2017". In the Reference list, data citations must be labeled with "[DATASET]". A data reference must provide the database name, accession number/identifiers and a resolvable link to the landing page from which the data can be accessed at the end of the reference. Further instructions are available at: https://link.springer.com/journal/44320/submission-guidelines#cms-Reference-guidelines
No public datasets were used, all data in this manuscript was generated for the manuscript and is included in data availability.

10) In the Methods, please take care of the following:
- The Materials and Methods section should be renamed to "Methods".
- Although you have indicated in the Author Checklist that a statement on blinding has been included in the Methods, we were not able to locate this statement. Please ensure that a statement on whether or not blinding was done is included in the Methods even if no blinding was done.
Section has been renamed and have included a statement on blinding for the animal experiments (pg15) and IMC image acquisition (pg19)

11) Please remove the Reagents and Tools table from the manuscript, as you have already supplied this as a separate file.
This has been removed from the manuscript.

12) Please place individual sections of the manuscript in the following order: Title page - Abstract & Keywords - Introduction - Results - Discussion - Methods - Data Availability - Acknowledgements - Disclosure and Competing Interests Statement - References - (Main Tables with legends if applicable) - Expanded View Figure Legends.
Sections have been reordered accordingly.

13) For the figures and figure legends, please take care of the following:
- Please ensure that the callouts for all figures are updated in the main manuscript - currently there are callouts for Figure S1 and S7, which need to be corrected

(potentially these should be Figure EV1/Figure EV7). A callout is missing for Figure 7G.
Fig S1 and S7 have been changed to EV1 and EV7. Figure 7G is cited in the results on page 10.

- In a routine image check, we note that there has been reuse of the same image between Figure 2A and Figure EV4A. While this is not an issue, it should be mentioned in both Figure legends that the figure has been reused.
This has now been included in the legend for each figure.

- Please note that information related to n is missing in the legend of figure EV1 C.
This is included in the legend "Points represent individual ROIs with the black horizontal bars showing the mean for all ROIs in that group; n = 2 - 4 animals with 3 corresponding ROIs per sample (c)"

- Please note that the exact p values are not provided in the legends of figures 3F, I; 4D, F, H, G, I; 5C, H; EV6 A, B.
Exact P values are shown for all data apart from if they are less than 0.0001 , as the significance value is capped at 0.0001 by the SAS software.

- Please indicate the statistical test used for data analysis in the legends of figures 1C, D; 3B, C, E, F, H, I; 4C, D, F, G, H, I, K, L, M.
Statistical tests used are now included within the figures and information also updated in the Methods section for statistical analysis.

14) Please note that funding information should be given in the "Acknowledgements" section (not in its own separate section).
Funding information has been moved to the acknowledgements section

15) Synopsis:
- Synopsis image: Please remove the Graphical Abstract from the main manuscript. The Graphical Abstract (referred to as Synopsis image at EMBO Press) should be uploaded as a separate high-resolution jpeg file 550 pixels wide x 300 pixels high.
- Synopsis text: Please provide a separate word document including a short standfirst (maximum of 300 characters, including spaces) and up to 5 bullet points to summarise the key NEW findings. They should be designed to be complementary to the abstract - i.e. not repeat the same text. We encourage inclusion of key acronyms and quantitative information (maximum of 30 words / bullet point). Please use the passive voice.
- Please check your synopsis text and image before submission with your revised manuscript. Please be aware that in the proof stage minor corrections only are allowed (e.g., typos).
Synopsis is now included and has been uploaded as a separate document

16) Source Data: Please ensure that a completed Source Data checklist is uploaded as a Related Manuscript File. Source Data should be organized as a single source data file (zipped) per figure for main figures (all EV and/or Appendix figure Source

Data can be included in a single folder), with the panels clearly visible in the folder structure instead of a single excel file for all Source Data. e.g. all the Source data files for figure 1 need to be saved in a single folder and this needs to be zipped and then uploaded as "SD figure 1.zip" file.
The correct format is uploaded for source data for all figures.

17) As part of the EMBO Publications transparent editorial process initiative (see our policy here: https://www.embopress.org/transparent-process#Review_Process), Molecular Systems Biology will publish online a Peer Review File (PRF) to accompany accepted manuscripts. This file will be published in conjunction with your paper and will include the anonymous referee reports, your point-by-point response and all pertinent correspondence relating to the manuscript. Let us know whether you agree with the publication of the PRF and as here, if you want to remove or not any figures from it prior to publication. Please note that the Authors checklist will be published at the end of the PRF.
We agree to the publication of this information

18) After your paper is published, we may promote it on social media. If you have any handles or hashtags for Bluesky you would like included, please let us know.
@DrTESutherland @james-ep

19) Please provide a point-by-point letter INCLUDING my comments and your detailed responses (as Word file).

Please let us know if we have missed anything

Regards
Tara Sutherland

6th Jul 2026
Manuscript number: MSB-2025-12944RR
Title: Spatial ECM mapping defines cellular matrix environments associated with allergic airway pathology

Dear Dr. Sutherland,

Thank you again for sending us your revised manuscript. We are now satisfied with the modifications made and I am pleased to inform you that your paper has been accepted for publication.

Your manuscript will be processed for publication by EMBO Press. It will be copy edited and you will receive page proofs prior to publication. Please note that you will be contacted by Springer Nature Author Services to complete licensing and payment information.

You may qualify for financial assistance for your publication charges - either via a Springer Nature fully open access agreement or an EMBO initiative. Check your eligibility: https://link.springer.com/journal/44320/how-to-publish-with-us

Should you be planning a Press Release on your article, please notify the production team via this form in order to coordinate publication and release dates: https://forms.cloud.microsoft/e/eH4A8JVP6g

If you have any questions, please do not hesitate to contact the Editorial Office. Thank you for your contribution to Molecular Systems Biology.

Yours sincerely,

Poonam Bheda, PhD
Scientific Editor
Molecular Systems Biology

>>> Please note that it is Molecular Systems Biology policy for the transcript of the editorial process (containing referee reports and your response letter) to be published as an online supplement to each paper. If you do NOT want this, you will need to inform the Editorial Office via email immediately. More information is available here: https://link.springer.com/partners/embo-press/editorial-policies#Peer%20review

EMBO Press Author Checklist

Corresponding Author Name: Tara Sutherland	
Journal Submitted to: Moelcular Systems Biology	
Manuscript Number: MSB-2025-12944-T	

USEFUL LINKS FOR COMPLETING THIS FORM
The EMBO Journal - Author Guidelines
EMBO Reports - Author Guidelines
Molecular Systems Biology - Author Guidelines
EMBO Molecular Medicine - Author Guidelines

Reporting Checklist for Life Science Articles (updated January
This checklist is adapted from Materials Design Analysis Reporting (MDAR) Checklist for Authors. MDAR establishes a minimum set of requirements in transparent reporting in the life sciences (see Statement of Task: 10.31222/osf.io/9sm4x). Please follow the journal's guidelines in preparing your
Please note that a copy of this checklist will be published alongside your article.

Abridged guidelines for figures
1. Data
The data shown in figures should satisfy the following conditions:
- ➞ the data were obtained and processed according to the field's best practice and are presented to reflect the results of the experiments in an accurate and unbiased manner.
- ➞ ideally, figure panels should include only measurements that are directly comparable to each other and obtained with the same assay.
- ➞ plots include clearly labeled error bars for independent experiments and sample sizes. Unless justified, error bars should not be shown for technical
- ➞ if n<5, the individual data points from each experiment should be plotted. Any statistical test employed should be justified.
- ➞ Source Data should be included to report the data underlying figures according to the guidelines set out in the authorship guidelines on Data

2. Captions
Each figure caption should contain the following information, for each panel where they are relevant:
- ➞ a specification of the experimental system investigated (eg cell line, species name).
- ➞ the assay(s) and method(s) used to carry out the reported observations and measurements.
- ➞ an explicit mention of the biological and chemical entity(ies) that are being measured.
- ➞ an explicit mention of the biological and chemical entity(ies) that are altered/varied/perturbed in a controlled manner.
- ➞ the exact sample size (n) for each experimental group/condition, given as a number, not a range;
- ➞ a description of the sample collection allowing the reader to understand whether the samples represent technical or biological replicates (including how many animals, litters, cultures, etc.).
- ➞ a statement of how many times the experiment shown was independently replicated in the laboratory.
- ➞ definitions of statistical methods and measures:
  - common tests, such as t-test (please specify whether paired vs. unpaired), simple χ2 tests, Wilcoxon and Mann-Whitney tests, can be unambiguously identified by name only, but more complex techniques should be described in the methods section;
  - are tests one-sided or two-sided?
  - are there adjustments for multiple comparisons?
  - exact statistical test results, e.g., P values = x but not P values < x;
  - definition of 'center values' as median or average;
  - definition of error bars as s.d. or s.e.m.

Please complete ALL of the questions below.
Select "Not Applicable" only when the requested information is not relevant for your study.

Materials

Newly Created Materials	Information included in the manuscript?	In which section is the information available? (Reagents and Tools Table, Materials and Methods, Figures, Data Availability Section)
New materials and reagents need to be available; do any restrictions apply?	Not Applicable	

Antibodies	Information included in the manuscript?	In which section is the information available? (Reagents and Tools Table, Materials and Methods, Figures, Data Availability Section)
For **antibodies** provide the following information:<br>- Commercial antibodies: RRID (if possible) or supplier name, catalogue number and or/clone number<br>- Non-commercial: RRID or citation	Yes	Reagents and Tools Table; Tables 1, 2, 3 also provide concentations, clones etc.

DNA and RNA sequences	Information included in the manuscript?	In which section is the information available? (Reagents and Tools Table, Materials and Methods, Figures, Data Availability Section)
**Short novel DNA or RNA including primers, probes**: provide the sequences.	Not Applicable	

Cell materials	Information included in the manuscript?	In which section is the information available? (Reagents and Tools Table, Materials and Methods, Figures, Data Availability Section)
**Cell lines:** Provide species information, strain. Provide accession number in repository **OR** supplier name, catalog number, clone number, and/**OR** RRID.	Not Applicable	
**Primary cultures:** Provide species, strain, sex of origin, genetic modification status.	Not Applicable	
Report if the cell lines were recently **authenticated** (e.g., by STR profiling) and tested for mycoplasma contamination.	Not Applicable	

Experimental animals	Information included in the manuscript?	In which section is the information available? (Reagents and Tools Table, Materials and Methods, Figures, Data Availability Section)
**Laboratory animals or Model organisms:** Provide species, strain, sex, age, genetic modification status. Provide accession number in repository **OR** supplier name, catalog number, clone number, **OR** RRID.	Yes	Reagents and Tools Table; Materials and Methods
**Animal observed in or captured from the field:** Provide species, sex, and age where possible.	Not Applicable	
Please detail **housing and husbandry conditions**.	Yes	Materials and Methods

Plants and microbes	Information included in the manuscript?	In which section is the information available? (Reagents and Tools Table, Materials and Methods, Figures, Data Availability Section)
**Plants:** provide species and strain, ecotype and cultivar where relevant, unique accession number if available, and source (including location for collected wild specimens).	Not Applicable	
**Microbes:** provide species and strain, unique accession number if available, and source.	Not Applicable	

Human research participants	Information included in the manuscript?	In which section is the information available? (Reagents and Tools Table, Materials and Methods, Figures, Data Availability Section)
If collected and within the bounds of privacy constraints report on age, sex and gender or ethnicity for all study participants.	Not Applicable	

Core facilities	Information included in the manuscript?	In which section is the information available? (Reagents and Tools Table, Materials and Methods, Figures, Data Availability Section)
If your work benefited from core facilities, was their service mentioned in the acknowledgments section?	Yes	Acknowledgements

Design

Study protocol	Information included in the manuscript?	In which section is the information available? (Reagents and Tools Table, Materials and Methods, Figures, Data Availability Section)
If study protocol has been **pre-registered, provide DOI in the manuscript**. For clinical trials, provide the trial registration number **OR** cite DOI.	Not Applicable	
Report the **clinical trial registration number** (at ClinicalTrials.gov or equivalent), where applicable.	Not Applicable	

Laboratory protocol	Information included in the manuscript?	In which section is the information available? (Reagents and Tools Table, Materials and Methods, Figures, Data Availability Section)
Provide DOI OR other citation details if **external detailed step-by-step protocols** are available.	Not Applicable	

Experimental study design and statistics	Information included in the manuscript?	In which section is the information available? (Reagents and Tools Table, Materials and Methods, Figures, Data Availability Section)
Include a statement about **sample size** estimate even if no statistical methods were used.	Yes	Materials and Methods
Were any steps taken to minimize the effects of subjective bias when allocating animals/samples to treatment (e.g. **randomization procedure**)? If yes, have they been described?	Yes	Materials and Methods
Include a statement about **blinding** even if no blinding was done.	Yes	Materials and Methods
Describe **inclusion/exclusion criteria** if samples or animals were excluded from the analysis. Were the criteria pre-established?<br><br>If sample or data points were omitted from analysis, report if this was due to attrition or intentional exclusion and provide justification.	Not Applicable	
For every figure, are **statistical tests** justified as appropriate? Do the data meet the assumptions of the tests (e.g., normal distribution)? Describe any methods used to assess it. Is there an estimate of variation within each group of data? **Is** the variance similar between the groups that are being statistically compared?	Yes	Materials and Methods and figure legends

Sample definition and in-laboratory replication	Information included in the manuscript?	In which section is the information available? (Reagents and Tools Table, Materials and Methods, Figures, Data Availability Section)
In the figure legends: state number of times the experiment was **replicated** in laboratory.	Yes	Figure legnds and materials and methods
In the figure legends: define whether data describe **technical or biological replicates**.	Yes	Figure legnds and materials and methods

Ethics

Ethics	Information included in the manuscript?	In which section is the information available? (Reagents and Tools Table, Materials and Methods, Figures, Data Availability Section)
Studies involving **human participants**: State details of **authority granting ethics approval** (IRB or equivalent committee(s), provide reference number for approval.	Not Applicable	
Studies involving **human participants**: Include a statement confirming that **informed consent** was obtained from all subjects and that the experiments conformed to the principles set out in the WMA Declaration of Helsinki and the Department of Health and Human Services Belmont Report.	Not Applicable	
Studies involving **human participants:** For publication of **patient photos**, include a statement confirming that consent to publish was obtained.	Not Applicable	
Studies involving experimental **animals**: State details of **authority granting ethics approval** (IRB or equivalent committee(s), provide reference number for approval. Include a statement of compliance with ethical regulations.	Yes	Materials and Methods
Studies involving **specimen and field samples:** State if relevant **permits** obtained, provide details of authority approving study; if none were required, explain why.	Not Applicable	

Dual Use Research of Concern (DURC)	Information included in the manuscript?	In which section is the information available? (Reagents and Tools Table, Materials and Methods, Figures, Data Availability Section)
Could your study fall under dual use research restrictions? Please check biosecurity documents and list of **select agents and toxins** (CDC): https://www.selectagents.gov/sat/list.htm	Not Applicable	
If you used a select agent, is the security level of the lab appropriate and reported in the manuscript?	Not Applicable	
If a study is subject to dual use research of concern regulations, is the name of the **authority granting approval and reference number** for the regulatory approval provided in the manuscript?	Not Applicable	

Reporting

The MDAR framework recommends adoption of discipline-specific guidelines, established and endorsed through community initiatives. Journals have their own policy about requiring specific guidelines and recommendations to complement MDAR.

Adherence to community standards	Information included in the manuscript?	In which section is the information available? (Reagents and Tools Table, Materials and Methods, Figures, Data Availability Section)
State if relevant guidelines or checklists (e.g., **ICMJE, MIBBI, ARRIVE, PRISMA**) have been followed or provided.	Yes	Materials and Methods
For **tumor marker prognostic studies,** we recommend that you follow the **REMARK** reporting guidelines (see link list at top right). See author guidelines, under 'Reporting Guidelines'. Please confirm you have followed these guidelines.	Not Applicable	
For **phase II and III randomized controlled trials**, please refer to the **CONSORT** flow diagram (see link list at top right) and submit the CONSORT checklist (see link list at top right) with your submission. See author guidelines, under 'Reporting Guidelines'. Please confirm you have submitted this list.	Not Applicable	

Data Availability

Data availability	Information included in the manuscript?	In which section is the information available? (Reagents and Tools Table, Materials and Methods, Figures, Data Availability Section)
Have **primary datasets** been deposited according to the journal's guidelines (see 'Data Deposition' section) and the respective accession numbers provided in the Data Availability Section?	Yes	Data Availability Section
Were **human clinical and genomic datasets** deposited in a public access-controlled repository in accordance to ethical obligations to the patients and to the applicable consent agreement?	Not Applicable	
Are **computational models** that are central and integral to a study available without restrictions in a machine-readable form? Were the relevant accession numbers or links provided?	Yes	Materials and Methods
If publicly available data were reused, provide the respective **data citations in the reference list**.	Not Applicable	
